# C_3_ cotyledons are followed by C_4_ leaves: intra-individual transcriptome analysis of *Salsola soda* (Chenopodiaceae)

**DOI:** 10.1093/jxb/erw343

**Published:** 2016-09-22

**Authors:** Maximilian Lauterbach, Kumari Billakurthi, Gudrun Kadereit, Martha Ludwig, Peter Westhoff, Udo Gowik

**Affiliations:** 1 Institut für Allgemeine und Spezielle Botanik und Botanischer Garten der Johannes Gutenberg-Universität Mainz, Mainz, Germany; 2 Institut für Entwicklungs- und Molekularbiologie der Pflanzen, Düsseldorf, Germany; 3 Cluster of Excellence on Plant Sciences (CEPLAS), Düsseldorf, Germany; 4 School of Chemistry and Biochemistry, University of Western Australia, Crawley, WA, Australia

**Keywords:** C_4_ photosynthesis, Caryophyllales, Chenopodiaceae, cotyledon, development, leaf, RNA seq, *Salsola*, transcriptome

## Abstract

Some species of Salsoleae (Chenopodiaceae) convert from C_3_ photosynthesis during the seedling stage to the C_4_ pathway in adult leaves. This unique developmental transition of photosynthetic pathways offers the exceptional opportunity to follow the development of the derived C_4_ syndrome from the C_3_ condition within individual plants, avoiding phylogenetic noise. Here we investigate *Salsola soda*, a little-studied species from tribe Salsoleae, using an ontogenetic approach. Anatomical sections, carbon isotope (δ^13^C) values, transcriptome analysis by means of mRNA sequencing, and protein levels of the key C_4_ enzyme phospho*enol*pyruvate carboxylase (PEPC) were examined from seed to adult plant stages. Despite a previous report, our results based on δ^13^C values, anatomy and transcriptomics clearly indicate a C_3_ phase during the cotyledon stage. During this stage, the entire transcriptional repertoire of the C_4_ NADP-malic enzyme type is detected at low levels compared to a significant increase in true leaves. In contrast, abundance of transcripts encoding most of the major photorespiratory enzymes is not significantly decreased in leaves compared to cotyledons. PEPC polypeptide was detected only in leaves, correlating with increased PEPC transcript abundance from the cotyledon to leaf stage.

## Teaser

The genome of *Salsola soda* allows a transition from C_3_ to C_4_ photosynthesis. A developmental transcriptome series revealed novel genes showing expression patterns similar to those encoding C_4_ proteins.

## Introduction

Our knowledge of the biochemistry of C_4_ photosynthesis (C_4_) has broadened enormously in the 50 years since its discovery (Hatch and Slack, 1966), as recent molecular and analytical tools offer new possibilities to also expand our understanding of the molecular genetics of C_4_. C_4_ is a carbon concentration mechanism that is thought to have evolved from C_3_ photosynthesis (C_3_) in response to conditions favouring photorespiration, for example hot and/or dry environments (Ehleringer *et al.*, 1991; Bauwe *et al.*, 2010). This mechanism is realized through a spatial division of labour between two compartments of the photosynthetically active organ, and in most C_4_ plants takes place between mesophyll (M) and bundle sheath (BS) tissues of leaves (Edwards *et al.*, 2004). This division of labour is accompanied by differential expression of genes encoding C_4_ proteins that function in the two compartments (Hibberd and Covshoff, 2010) and more transport processes are required in C_4_ than C_3_ (Weber and von Caemmerer, 2010). Additional changes are seen in leaf anatomy and gene regulation (reviewed in Gowik and Westhoff, 2011; Langdale, 2011). The polyphyletic origin of C_4_ – currently at least 60 independent origins are known, including both monocot and dicot lineages (Sage, 2016) – allows the investigation of C_4_ evolution in a wide range of plant groups.

During the last few years, our knowledge of the molecular genetics of C_4_ increased dramatically, as large-scale analysis of gene expression in non-model species (i.e. RNA sequencing) has become feasible and allowed extensive comparisons of gene expression between closely related C_3_ and C_4_ species. The understanding of C_4_ evolution has been significantly promoted by differential gene expression (DGE) analyses, for example in eudicots between C_3_ and C_4_ species of the genus *Flaveria* or from the Cleomaceae (e.g. Bräutigam *et al.*, 2011, 2014; Gowik *et al.*, 2011; Mallmann *et al.*, 2014; Külahoglu *et al.*, 2014; van den Bergh *et al.*, 2014; Aubry *et al.*, 2016). However, the greatest diversity of the C_4_ syndrome in eudicots is neither found in Asteraceae nor in Cleomaceae, but in the Amaranthaceae/Chenopodiaceae alliance (Pyankov *et al.*, 2001*a*; Kadereit *et al.*, 2003, 2012, 2014; Schütze *et al.*, 2003; Sage *et al.*, 2007; Kadereit and Freitag, 2011), where at least 15 independent C_4_ origins are proposed, ten of these within Chenopodiaceae *sensu stricto* (Sage, 2016). While several studies have highlighted a vast diversity in leaf anatomy (Edwards and Voznesenskaya, 2011) and biochemistry in different species of the Chenopodiaceae (Kadereit *et al.*, 2003), until now, only a single study compared transcriptomes between C_3_ and C_4_ types within this family (Li *et al.*, 2015). In that study, the transcriptomes of cotyledons conducting C_3_ and C_4_ assimilating shoots of *Haloxylon ammodendron* (C.A. Mey.) Bunge were compared. The differences detected are not caused by variation between species, but can be associated with differences between C_3_ and C_4_, or to organ-related variation. However, due to its tree-like growth form, *H. ammodendron* is, in practical terms, not ideal as a model species. On the other hand, it belongs to the tribe Salsoleae and within this tribe, several species are known that conduct C_3_ in cotyledons and C_4_ in assimilating shoots/leaves (Pyankov *et al.*, 1999, 2000, 2001*b*).

In the current study, we introduce *Salsola soda* L. from Salsoleae as a model system to study the developmental transition from C_3_ cotyledons to C_4_ leaves. *Salsola soda* offers several advantages for increasing our knowledge of C_4_. Firstly, the transition from C_3_ to C_4_ occurs naturally from cotyledons to leaves, as shown in this study by anatomical sections and carbon isotope data. Secondly, *S. soda* has an annual life cycle (Freitag, 1997; ML, personal observation) and it is easily cultivated in the greenhouse. *Salsola soda* seeds germinate easily and quickly, plants are self-compatible, and it produces many seeds each year (ML, personal observation). Finally, *S. soda* belongs to a tribe (Salsoleae) that is diverse in C_3_, C_3_-C_4_ intermediate and C_4_ species (Schüssler *et al.*, submitted).

We investigated the transition from a C_3_ to a C_4_ metabolism by conducting deep sequencing of transcriptomes (RNA-seq) of five different developmental stages, from young seedling to adult plant. We reveal detailed insights into gene expression patterns based on the different organs and/or stages. All the transcripts predicted for a NADP-malic enzyme (NADP-ME) type species were highly abundant in leaves while being mostly absent in cotyledons. Consequently, the results of this study present the opportunity to perform detailed targeted investigations of genes encoding potentially novel C_4_ proteins.

## Materials and methods

### Plant growth and sampled developmental stages

The *Salsola soda* seed sample used in this study was collected on a stony beach of the Adriatic coast south-west of Trieste, near Strunjan, in the Naravni nature reserve (Lagune Struzja Salinen; 45°31ʹ40.74ʹʹN, 13°36ʹ12.07ʹʹE) in Slovenia by M. Kaligaric s.n. (25 October 2012). A voucher of this collection is deposited at the herbarium of the University of Mainz (MJG no. 014562).

*Salsola soda* plants were grown in potting soil (custom mixed soil from the Botanical Garden, University of Mainz) in a glasshouse at the University of Mainz with supplementary light under a light intensity of ~300 µmol m^−2^ s^−1^. Samples were harvested during April and May 2014, between 10:30 and 13:00, and dried in silica, or immediately frozen in liquid nitrogen and stored at −80 °C for measurement of carbon isotope values and RNA extraction, respectively.

### Anatomy

Fresh plant material was fixed in AFE (acetic acid-formaldehyde-ethanol), dehydrated using an ethanol series, and embedded in Technovit 7100 (Heraeus Kulzer). Transverse sections, 5–10 µm thick, of the middle regions of fully developed cotyledons and leaves were made using a rotary microtome (Leitz) and stained in a 6:6:5:6 mixture of Azur II, Eosin Y, methylene blue and distilled water. Eukitt (O. Kindler) was used as the mounting medium and images of the sections were taken using a Leitz Diaplan light microscope combined with Leica Application Suite 2.8.1. For a more detailed description of the sectioning method, see Bohley *et al.* (2015) and Lauterbach *et al.* (2016).

### Stable carbon isotope measurements

Cotyledons and leaves of *S. soda* were harvested (see above) and dried for several days in silica gel. Dry leaf samples were pulverized using the mixer mill MM 301 (Retsch). Approximately 200mg of each sample were used to determine stable carbon isotope ratios (i.e. ^13^C/^12^C) relative to the Pee Dee belemnite standard (Craig, 1957) by the Institut für Geowissenschaften at the University of Mainz. Ten different developmental stages were sampled and measured (each stage with technical triplicates): (i) dry seed, (ii) imbibed seed, (iii) young cotyledon, (iv) mature cotyledon, (v) young first leaf pair, (vi) mature first leaf pair, (vii) young second leaf pair, (viii) mature second leaf pair, (ix) mature third leaf pair, and (x) mature leaf of an adult plant.

### RNA extraction, library preparation, mRNA sequencing

Total RNA was isolated from 18–120mg ground plant tissue with the RNeasy Plant Mini Kit (Qiagen) following the manufacturer’s protocol (January 2011), including DNase digestion with the RNase-Free DNase Set (Qiagen). RNA quality and quantity were determined with the 2100 Bioanalyzer (Agilent Technologies), NanoDrop (Thermo Fisher Scientific), and Qubit (Life Technologies). For each sample, 500ng of high-quality, total RNA (RNA Integrity Number between 8.8 and 9.6) were used for cDNA library preparation with the TruSeq RNA Sample Preparation Kit (Illumina Inc.), following the Low Sample Protocol (TruSeq RNA Sample Preparation v2 Guide, Illumina Proprietary, Part # 15026495 Rev. C, May 2012). Sequence determination of 100bp single reads was performed on an Illumina HiSeq2000 platform. Five different developmental stages of individual *S. soda* plants were included in the experiment (each with biological triplicates): stage 1 (yS), whole imbibed seed 2 d after germination; stage 2 (Cot), fully expanded, mature cotyledon, where the tip (±10mm) of the first leaf pair was visible (~6 d after germination); stage 3 (1L), mature first leaf pair and at this stage the tip of the second leaf pair was visible (~20 d after germination); stage 4 (2L), mature second leaf pair and again at this stage the tip of the third leaf was visible (~40 d after germination); and stage 5 (oL), mature leaf pair of an adult plant (~4 months after germinating).

### Mapping, statistics, data analysis, gene expression

Sequence reads were checked for quality with FASTQC tool (http://www.bioinformatics.babraham.ac.uk/projects/fastqc/), and filtered and trimmed using FASTX toolkit (http://hannonlab.cshl.edu/fastx_toolkit/). Raw reads were mapped onto coding sequences of the ‘minimal’ TAIR 9 transcriptome (i.e. genome duplications in the lineage of Brassicaceae and tandem duplicated genes were reduced to one representative for each; Bräutigam *et al.*, 2011) by BLAT (Kent, 2002), and the best hit for each read was counted. Gene expression was normalized to reads per kilobase coding sequence per million mappable reads (RPKM) and a threshold of 1 RPKM per coding sequence present in at least one stage was defined as true transcription. Differentially expressed transcripts were determined by edgeR (Robinson *et al.*, 2010) in R (R Core Team, 2016). Log_2_ expression ratios were calculated and a significance threshold of 0.01 was defined after Benjamini-Hochberg correction to account for multiple testing (Benjamini and Hochberg, 1995). A log_2_ fold change (FC) of ≥1 was applied to call differentially expressed genes. To identify and visualize expression patterns of interest, hierarchical clustering using Euclidean distance (Eisen *et al.*, 1998) of all samples was performed with the MeV 4.9 software (MultiExperiment Viewer, http://www.tm4.org/mev.html). To investigate which biological functions were active at each stage, transcriptional investment (defined as percentage of all transcripts with at least one RPKM in one stage belonging to a particular MapMan category) was analysed using MapMan (www.mapman.gabipd.org/;Thimm *et al.*, 2004). Gene ontology analyses were performed using GORILLA (Gene Ontology enRIchment anaLysis and visuaLizAtion tool, Eden *et al.*, 2007, 2009) online service. K-means clustering (K=20 clusters; Pearson correlation; Soukas *et al.*, 2000) of all expressed transcripts with ≥1 RPKM were performed using MeV 4.9 software.

### Immunoblot analysis

Proteins were extracted from eight developmental stages (young seedling, mature cotyledon, young first leaf pair, mature first leaf pair, young second leaf pair, mature second leaf pair, young third leaf pair, and an old leaf pair of an adult plant) in ground glass homogenizers following the method of Martinez *et al.* (1999), and protein concentration was determined using the Bio-Rad DC Protein Assay kit (Bio-Rad, Hercules, CA, USA) with bovine serum albumin as the standard. Equal amounts of total soluble protein were separated on 12% (w/v) resolving gels (Laemmli, 1970), and transferred to nitrocellulose membrane using a semi-dry transfer method (Ludwig *et al.*, 1998). After transfer, blots were rinsed briefly three times in 12.5mM Tris-HCl, pH 8.0, 137mM NaCl, and 2.7mM KCl (TBS) containing 1% (v/v) Tween 20 (TBST), and non-specific sites were blocked by incubation in TBST containing 5% (w/v) skim milk powder (blocking buffer) for at least 1h. The blots were then incubated in a 1:10 000 dilution of rabbit anti-maize PEPC antiserum (Chemicon) in blocking buffer for 1h at room temperature, washed three times for 5min each, in TBST, and labelled with a 1:3000 dilution of horseradish peroxidase coupled anti-rabbit IgG secondary antibody (GE Healthcare) in blocking buffer. After three 5-min washes in TBS, immunoreactive polypeptides were visualized and imaged using chemiluminescence (ECL Western Blotting Substrate; Pierce) and a Bio-Rad Chemi-Doc MP.

## Results

### Anatomical survey shows C_3_ anatomy in cotyledons and C_4_ anatomy in leaves of *Salsola soda*

**Fig. 1.**
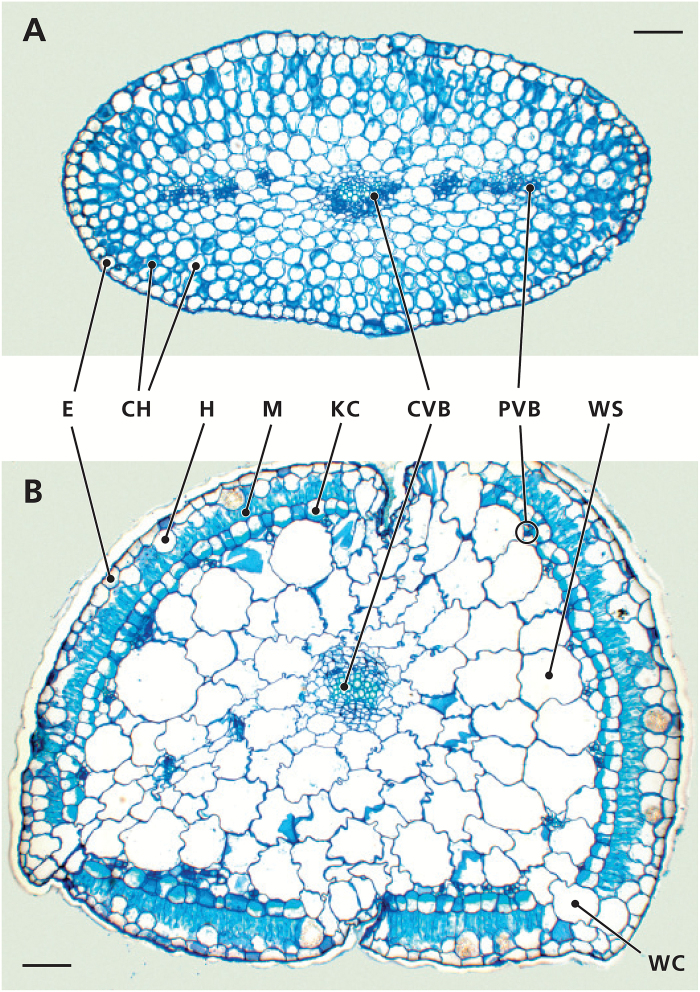
Anatomy of *Salsola soda* photosynthetic organs. Transverse sections of (A) cotyledon and (B) leaf of *S. soda*. CH, chlorenchyma; CVB, central vascular bundle; E, epidermis; H, hypodermis; KC, Kranz cells; M, mesophyll; PVB, peripheral vascular bundle; WC, window cells; WS, water storage tissue. Scale bars, 100 µm.

Cotyledons of *S. soda* are slightly flattened to biconvex and moderately succulent. Transverse sections of cotyledons show an isobilateral C_3_ anatomy, with one plane of vascular bundles (VB), including one distinct, main VB in the centre and up to six smaller VB surrounded by weakly developed water storage (WS) tissue (Fig. 1A). Towards the outside of the organ, the WS tissue is gradually replaced by the chlorenchyma, which consists of two to three layers of round to oval M cells (Fig. 1A). In contrast, *S. soda* leaves are terete, succulent and exhibit C_4_ anatomy. Transverse sections show a large central zone of WS tissue with one distinct main VB in the centre, and many (>20) smaller VB at the periphery, most in close contact with Kranz cells (KC; Fig. 1B). Chloroplasts of the KC layer are centripetally positioned. On the outer side of the KC layer is one layer of M cells, containing numerous chloroplasts. Surrounding the M is subepidermal non-chlorenchymatous tissue, called the hypodermis, and external to the hypodermis, a one-layered epidermis. Additionally, leaves of *S. soda* have three to four ‘windows’, which are gaps in the wreath composed of M and KC tissues, filled by two to three cells of WS tissue (Fig. 1B). These windows are visible at the leaf surface with the naked eye, and appear as whitish stripes with proximal-distal orientation.

### Carbon isotope values confirm C_3_ metabolism in cotyledons and C_4_ metabolism in leaves

**Fig. 2.**
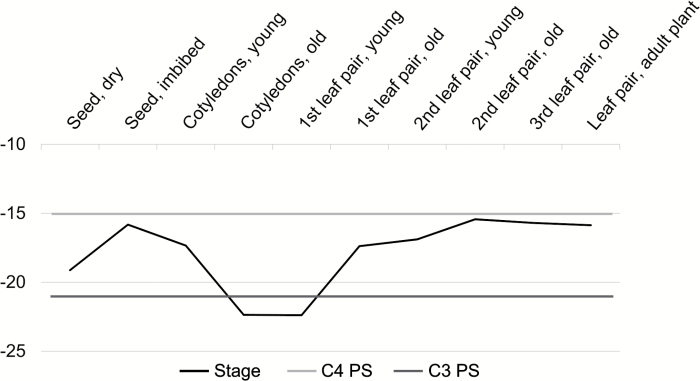
Stable carbon isotopes of ten developmental stages of *Salsola soda*. Typical C_4_ values are −15 and above (less negative, indicated as light grey line) and typical C_3_ values are −22 and below (more negative, indicated as dark grey line); measurements are shown as black line (actual values can be found in Supplementary Table S1).

Stable carbon isotope discrimination can be used as an indicator of C_4_ metabolism, since C_4_ plants discriminate less than C_3_ plants against ^13^C (O’Leary, 1981; Cernusak *et al.*, 2013). Carbon isotope values (δ ^13^C) of the ten developmental stages from dry seed to the mature leaf of an adult plant confirm the anatomical observations: cotyledons of *S. soda* appear to perform C_3_ whereas leaves perform C_4_ (Fig. 2, Supplementary Table S1 at *JXB* online). Starting with C_4_-like δ ^13^C values in imbibed seeds and young cotyledons (−15.83 and −17.34), the δ ^13^C value clearly decreases and falls into a C_3_-like range during the mature cotyledon and young first leaf pair stages (−22.37 and −22.39). After this C_3_ phase of the development, the δ ^13^C value increases again, and C_4_ values are exhibited from the mature first leaf pair stage onwards (Fig. 2, Supplementary Table S1).

### Transcriptomes of five *Salsola soda* developmental stages have comparable quality

We looked at DGE by means of mRNA sequencing in five developmental stages of *S. soda*, including (i) imbibed seed (yS), (ii) mature cotyledons (Cot), (iii) mature primary leaves (1L), (iv) mature secondary leaves (2L), (v) leaves of a 4-month-old plant (oL).

mRNA sequencing yielded between 25.5 and 38.8 million single-end raw reads per replicate, with at least 97.3% or more high-quality reads remaining after trimming and quality filtering (Table 1). Data are available at http://www.ncbi.nlm.nih.gov/sra/ (Supplementary Table S2). Since read quality was found to be very high, we decided to map all reads (i.e. raw reads) against protein-coding sequences of Arabidopsis. We were able to map between 9.6 and 17.9 million of the raw reads (30.7–47.8%). The mapping specificity between all samples remained the same (always reads of the same species, *S. soda*, were mapped onto coding sequences of Arabidopsis) and thus mapping was comparable between all five stages (Supplementary Table S3). Between 12252 and 13189 genes were expressed with at least one RPKM per stage, and altogether we identified 14119 different genes with at least one RPKM.

**Table 1.** Summary of sequencing information: number of raw and clean reads, total and % of mapped reads onto minimal TAIR9 transcriptome, and reads with >1 RPKM, >500 RPKM, and >1000 RPKM of the five developmental stages: imbibed seed (yS), mature cotyledons (Cot), mature primary leaves (1L), mature secondary leaves (2L), and leaves of a 4-month-old plant (oL)

Mean >1 RPKM	yS			Cot			1L			2L			oL		
	12252			13189			12832			12508			12849		
	yS_1	yS_2	yS_3	Cot_1	Cot_2	Cot_3	1L_1	1L_2	1L_3	2L_1	2L_2	2L_3	oL_1	oL_2	oL_3
No. of raw reads (in m.)	30.55	33.71	31.25	30.43	38.26	34.46	37.41	37.83	25.54	30.46	37.89	36.38	29.87	35.74	35.47
No. of clean reads (in m.)	29.93	32.82	30.48	29.88	37.47	33.78	36.70	37.12	24.96	29.92	37.17	35.72	29.33	35.05	34.80
No. of clean reads (in %)	98.0	97.3	97.5	98.2	97.9	98.0	98.1	98.1	97.7	98.2	98.1	98.2	98.2	98.1	98.1
No. of mapped reads (in m.)	11.42	11.44	9.58	12.74	16.44	13.40	16.95	17.42	11.29	14.57	17.87	17.23	11.88	15.38	14.09
No. of mapped reads (in %)	37.4	33.9	30.7	41.9	43.0	38.9	45.3	46.0	44.2	47.8	47.2	47.4	39.8	43.0	39.7
No. of genes with >1 RPKM	11793	12498	11168	13161	13196	12974	12691	12902	12720	12423	12534	12535	12941	12608	12852
No. of genes with >500 RPKM	257	206	252	257	274	221	223	267	239	237	232	233	201	197	215
No. of genes with >1000 RPKM	109	82	118	81	90	86	84	94	80	86	86	88	83	84	83

### Large-scale comparison of expression patterns of the *Salsola soda* developmental stages show quantitative differences

**Fig. 3.**
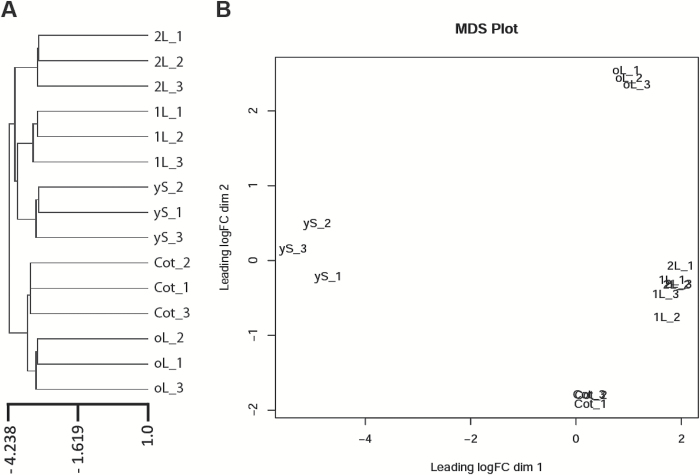
Transcript similarity of the five sampled *Salsola soda* developmental stages. Triplicate values for each stage are represented as (A) hierarchical clustering of all transcripts and (B) multidimensional scaling (MDS) plot of all transcripts. yS_1-3, young seedling; Cot_1-3, cotyledon; 1L_1-3, first leaf pair; 2L_1-3, second leaf pair; oL_1-3, leaf pair old plant.

According to their transcript profiles, replicates of each *S. soda* developmental stage group closely together in hierarchical clustering (Fig. 3A) and in a multidimensional scaling plot (Fig. 3B). In hierarchical clustering, the oL samples group more closely with Cot samples, while 1L and 2L samples differ from the oL replicates (Fig. 3A). In the multidimensional scaling plot, yS and oL samples are clearly distinct to all other stages (Fig. 3B). The 1L and 2L samples group closely together, and the Cot samples are nearest to the 1L-2L cluster (Fig. 3B).

**Fig. 4.**
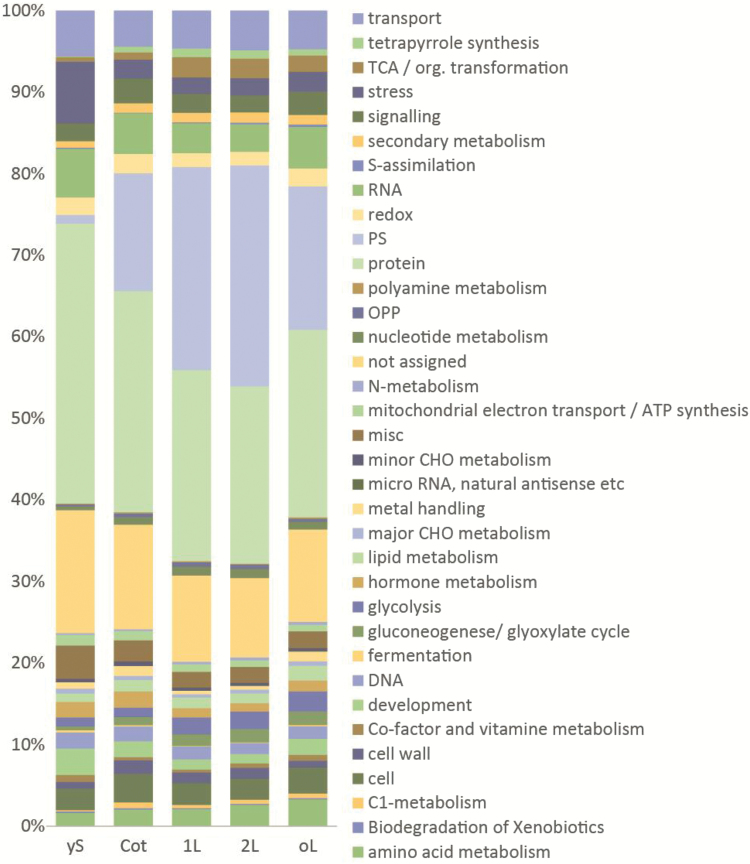
Distribution of transcriptional investment. Transcriptional investment is defined as the percentage of all transcripts belonging to a particular MapMan category (for category definitions see www.mapman.gabipd.org/).

In all five stages of *S. soda* development that were investigated, transcriptional investment is highest in the ‘protein’ MapMan bin (Fig. 4). In the earliest stage of imbibed seeds (yS), this category includes up to one third of all transcripts. Other MapMan bins containing abundant transcripts at all developmental stages (Fig. 4) are ‘not assigned’ (9.74–15.07%), ‘RNA’ (3.41–5.98%), and ‘transport’ (4.42–5.63%). MapMan bin ‘PS’ (photosynthesis) contains a high percentage of transcripts (14.44–27.08%) at all stages except yS (1.07%), with the highest percentage at stages 1L and 2L (24.86% and 27.08%, respectively). In contrast to ‘PS’, the ‘stress’ bin has the highest transcript pool at the yS stage (7.54%) compared to other stages (2.03–2.40%).

Enriched gene ontology (GO) terms that are significantly (FDR q-value ≤0.01) more abundant at a particular stage compared with the other stages correlate with results from the MapMan bin analysis. Enriched GO terms within the 3311 transcripts showing increased abundance in yS compared to Cot encode proteins involved in processes related to transport, especially ‘nuclear transport’, ‘protein transport’ and ‘RNA transport’ (Table 2). The 3630 transcripts showing increased abundance in Cot compared to yS are enriched in GO terms related to biosynthetic processes, lipid metabolism, pyruvate metabolism, glycolysis and photosynthesis (Table 2, Supplementary Table S4).

**Table 2.** Gene Ontology (GO) terms enriched in *Salsola soda* developmental transcriptomes. Top ten GO terms enriched in one stage (first column) compared to a certain other stage (second column) ranked by *P*-value (see Supplementary Table S4 for all GO terms with *P*-value ≤0.01 and FDR q-value ≤0.01)

Stage	Upregulated compared to	Enriched GO term	*P*-value	FDR q-value	Description
yS	Cot	GO:0006913	2.11E-13	6.47E-10	nucleocytoplasmic transport
		GO:0051169	2.11E-13	3.24E-10	nuclear transport
		GO:0034504	7.30E-12	7.48E-09	protein localization to nucleus
		GO:0006606	7.30E-12	5.61E-09	protein import into nucleus
		GO:1902593	7.30E-12	4.49E-09	single-organism nuclear import
		GO:0051170	7.30E-12	3.74E-09	nuclear import
		GO:0051028	1.29E-08	5.68E-06	mRNA transport
		GO:0050658	5.96E-08	2.29E-05	RNA transport
		GO:0050657	5.96E-08	2.03E-05	nucleic acid transport
		GO:0051236	5.96E-08	1.83E-05	establishment of RNA localization
Cot	yS	GO:0019682	4.20E-23	1.29E-19	glyceraldehyde-3-phosphate metabolic process
		GO:0006081	4.89E-18	7.51E-15	cellular aldehyde metabolic process
		GO:0044710	2.18E-17	2.23E-14	single-organism metabolic process
		GO:0006629	3.24E-17	2.49E-14	lipid metabolic process
		GO:0019288	9.31E-16	5.72E-13	isopentenyl diphosphate biosynthetic process, methylerythritol 4-phosphate pathway
		GO:0009240	9.31E-16	4.77E-13	isopentenyl diphosphate biosynthetic process
		GO:0046490	9.31E-16	4.08E-13	isopentenyl diphosphate metabolic process
		GO:0008610	1.94E-15	7.43E-13	lipid biosynthetic process
		GO:0044711	2.67E-15	9.11E-13	single-organism biosynthetic process
		GO:0044699	9.08E-14	2.79E-11	single-organism process
1L	Cot	GO:0016556	2.81E-08	8.63E-05	mRNA modification
		GO:0009653	6.62E-08	1.02E-04	anatomical structure morphogenesis
		GO:0005984	2.99E-07	3.07E-04	disaccharide metabolic process
		GO:0019682	1.01E-06	7.74E-04	glyceraldehyde-3-phosphate metabolic process
		GO:0044550	1.23E-06	7.53E-04	secondary metabolite biosynthetic process
		GO:0010103	1.92E-06	9.83E-04	stomatal complex morphogenesis
		GO:0090626	1.92E-06	8.42E-04	plant epidermis morphogenesis
		GO:0009311	3.77E-06	1.45E-03	oligosaccharide metabolic process
		GO:0007169	4.16E-06	1.42E-03	transmembrane receptor protein tyrosine kinase signaling pathway
		GO:0007167	4.16E-06	1.28E-03	enzyme linked receptor protein signalling pathway
	2L	none	---	---	---
2L	1L	none	---	---	---
oL	1L+2L	GO:0031347	6.78E-09	2.08E-05	regulation of defence response
		GO:0080134	1.50E-08	2.31E-05	regulation of response to stress
		GO:0006811	1.06E-07	1.08E-04	ion transport
		GO:1901698	2.86E-07	2.20E-04	response to nitrogen compound
		GO:0042537	3.33E-07	2.05E-04	benzene-containing compound metabolic process
		GO:0048583	4.38E-07	2.24E-04	regulation of response to stimulus
		GO:0043207	5.94E-07	2.61E-04	response to external biotic stimulus
		GO:0009607	7.64E-07	2.93E-04	response to biotic stimulus
		GO:0045088	1.21E-06	4.14E-04	regulation of innate immune response
		GO:0050896	1.37E-06	4.19E-04	response to stimulus

When the 1L stage is compared to Cot, mRNA metabolism, including modification and editing of mRNA, anatomical structure organization and development, stomata and epidermis morphogenesis, carbohydrate metabolism, and secondary metabolite biosynthesis are enriched processes among the 1046 transcripts (Table 2, Supplementary Table S4). While leaf development in 1L stage might not be entirely complete, processes not absolutely required for growth like secondary metabolism are already present. In contrast, we found no enriched GO term when comparing 1L stage and 2L stage (Table 2), which is consistent with both the hierarchical clustering and multidimensional scaling plot analyses, indicating the similarity of the 1L and 2L samples (Fig. 3).

Comparing stage oL to stages 1L and 2L, we found the 786 transcripts showing increased abundance were enriched in GO terms that reflect regulatory functions in response and/or defence to stress and biotic stimuli, for example ‘regulation of defence response’, ‘regulation of response to stress’, ‘ion transport’ and ‘response to stimulus’ (Table 2).

### Differentially expressed marker/signature genes among stages

**Fig. 5.**
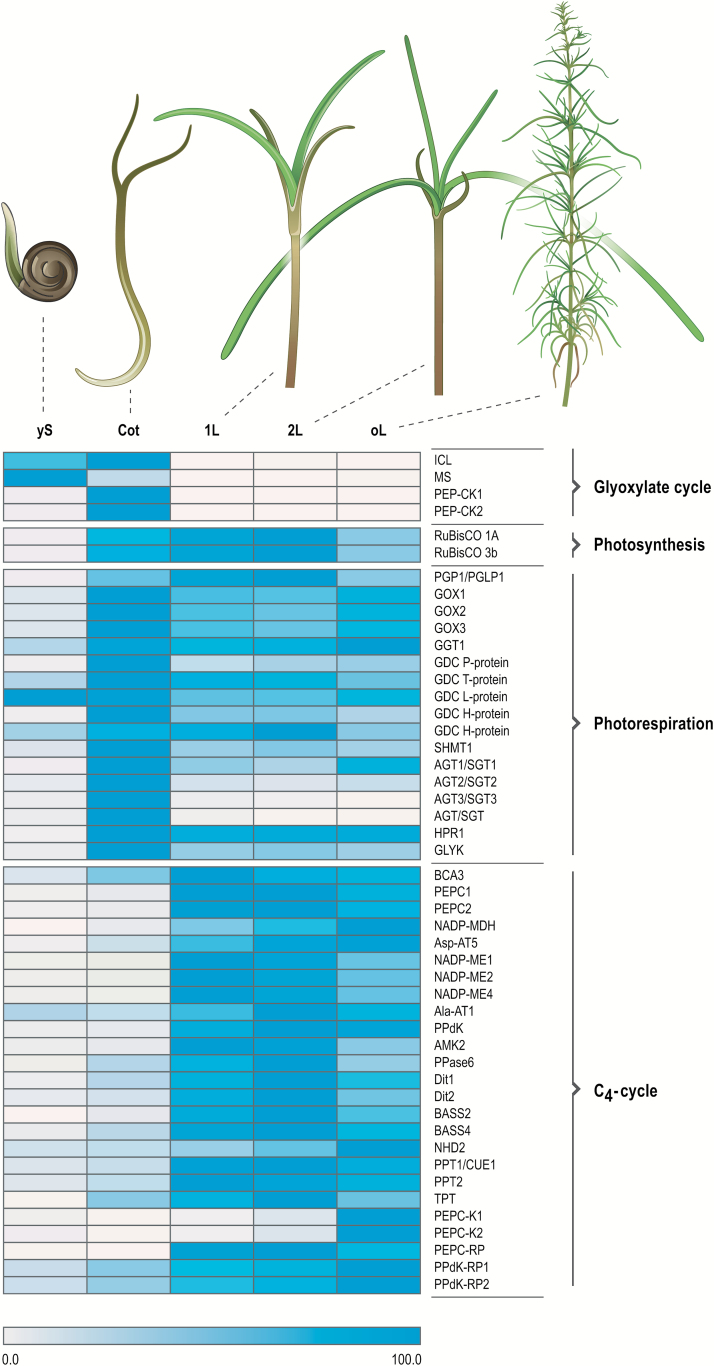
Relative transcript abundance of selected genes during development of Salsola soda. Transcript abundances are plotted as a heat map, which includes four genes coding for proteins of the glyoxylate cycle, two RuBisCO subunit genes, 17 genes encoding photorespiratory proteins, and 25 genes coding for C4-associated proteins at the five developmental stages: imbibed seed (yS), mature cotyledons (Cot), mature primary leaves (1L), mature secondary leaves (2L), and leaves of a four-month old plant (oL). ICL, isocitrate lyase; MS, malate synthase; PEP-CK, PEP carboxykinase; RuBisCO small subunit 1A; RuBisCO small subunit 3b; PGP1/PGLP1, 2-phosphoglycolate phosphatase 1; GOX, glycolate oxidase; GGT1, glutamate:glyoxylate aminotransferase 1; GDC, glycine decarboxylase; SHMT1, serine hydroxymethyltransferase; AGT/SGT, serine:glyoxylate aminotransferase; HPR1, NADH-dependent hydroxypyruvate reductase 1; GLYK, glycerate 3-kinase; BCA3, β-carbonic anhydrase 3; PEPC, PEP carboxylase; NADP-MDH, NADP-dependent malate dehydrogenase; Asp-AT5, aspartate aminotransferase; NADP-ME, NADP-dependent malate enzyme; Ala-AT1, alanine aminotransferase 1; PPdK, pyruvate orthophosphate dikinase; AMK2, adenosine monophosphate kinase; PPase6, pyrophosphatase; Dit, dicarboxylate transporter; BASS, bile acid:sodium symporter family protein; NHD2, sodium:hydrogen antiporter; PPT/CUE, PEP/phosphate translocator; TPT, triose phosphate translocator; PEPC-K, PEP carboxylase kinase; PEPC-RP, PEP carboxylase-related pro ein; PPdK-RP, PPdK-related protein.(see Supplementary Table S5 for transcript details).

Genes coding for proteins involved in post-germination seedling growth/early developmental stages are preferentially expressed in yS and Cot but not in the leaf stages. These genes include those encoding two key enzymes of the glyoxylate cycle, isocitrate lyase and malate synthase (Fig. 5, Supplementary Table S5). The glyoxylate cycle functions in the mobilization of sugars from storage lipids during developing seed and seedling stages, and takes place in the glyoxysomes. Specifically, acetyl-CoA arising from beta oxidation gets converted to organic compounds, which then enter the citric acid cycle in the mitochondria, resulting in the formation of oxaloacetate, which in turn is decarboxylated by phospho*enol*pyruvate carboxykinase (PEP-CK) to form CO_2_ and PEP, with the latter entering gluconeogenesis. In *Cucurbita pepo*, PEP-CK activity is low in the first 2 d of seed germination and dramatically increases between days 2 and 4, followed by no detectable activity in young leaves (Leegood and Ap Rees, 1978). Our findings are consistent with this scheme: *S. soda* sequences mapping to one of the two Arabidopsis PEP-CK genes encoding PEP-CK 1 and PEP-CK 2, are low in the yS stage, highly abundant in Cot, and are essentially absent in all three leaf stages (Fig. 5, Supplementary Table S5). In contrast to the yS stage, many transcripts coding for proteins involved in photosynthesis are abundant in the Cot sample, including those coding for RuBisCO (AT1G67090, AT5G38410), several chlorophyll a/b binding proteins (e.g. AT1G61520, AT2G05070, AT3G47470, AT5G54270), photosystem I (PSI) subunits (e.g. AT1G30380, AT2G20260, AT4G12800, AT5G64040) and photosystem II (PSII) subunits (e.g. AT1G51400, AT1G79040, AT2G06520) (Supplementary Table S5). In the 1L and 2L stages, 30 out of 130 genes assigned to the category ‘photosynthesis’ show significant increase in transcript abundance compared to the Cot stage, and transcripts from 81 genes are increased in abundance compared to yS (Supplementary Table S5). These results indicate that while photosynthesis is functional at the Cot stage, but not at yS, it is more fully engaged in leaves.

### Transcripts encoding proteins of C_4_ photosynthesis are highly abundant in leaf stages

Since a major focus of this study was the developmental transition from C_3_ to C_4_ in *S. soda*, we examined the abundance of transcripts coding for known core C_4_ cycle genes, C_4_-related transporters and C_4_-related regulatory proteins, with emphasis on stages Cot (representing C_3_), and 1L and 2L (representing C_4_).

Transcripts encoding most of the known proteins of the NADP-ME subtype of C_4_ were significantly (FDR q-value ≤0.01, and log_2_ fold-change of ≥1) more abundant in leaves compared to Cot (Fig. 5, Supplementary Table S5). These included phospho*enol*pyruvate carboxylase (PEPC) 1 and PEPC2, NADP-dependent malate dehydrogenase (NADP-MDH), aspartate aminotransferase 5 (Asp-AT5), NADP-dependent malic enzyme (NADP-ME) 1, NADP-ME2 and NADP-ME4, alanine aminotransferase 1 (Ala-AT1) and pyruvate orthophosphate dikinase (PPDK).

**Fig. 6.**
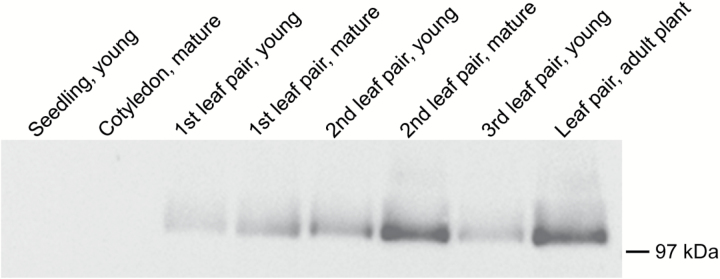
Immunoblot labelling of phospho*enol*pyruvate carboxylase. Total protein (12 µg) extracted from eight different developmental stages of *Salsola soda* were separated on a denaturing gel, blotted to nitrocellulose, and labelled with a rabbit anti-maize phospho*enol*pyruvate carboxylase antiserum. The position of the 97kDa molecular mass marker is shown on the right.

The detection of transcripts encoding two PEPC isoforms, PEPC1 and PEPC2, might be an artefact due to erroneous read mappings on the closest orthologue in Arabidopsis, and was observed in previous studies (Gowik *et al.*, 2011; Külahoglu *et al.*, 2014). As PEPC plays a pivotal role in the C_4_ pathway, and transcript abundance does not always correlate with protein levels (Vélez-Bermúdez and Schmidt, 2014), the abundance of PEPC protein was also investigated in cotyledon and leaves of *S. soda*. When blots of cotyledon and leaf soluble protein extracts were labelled with an anti-PEPC antiserum, a single immunoreactive polypeptide of ~100kDa (which is consistent with the mass of other plant PEPC proteins, O’Leary *et al.*, 2011), was only detected in the leaf extracts. The older leaves demonstrated the more intense labelling (Fig. 6).

Increased abundance of transcripts encoding C_4_-related transporters was found for dicarboxylate transporter (DiT) 1, DiT2, bile acid:sodium symporter family protein (BASS) 2, BASS4, sodium:hydrogen antiporter 2 (NHD2), phospho*enol*pyruvate/phosphate translocator (PPT) 1 (also known as CUE1) and PPT2 (Fig. 5, Supplementary Table S5). Increased levels of mRNA were detected for C_4_-related regulatory and auxiliary proteins, including adenosine monophosphate kinase 2 (AMK2), pyrophosphatase 6 (PPase6), phospho*enol*pyruvate carboxylase kinase 1 (PEPC-K1), PEPC-related protein (PEPC-RP) and PPDK regulatory protein 2 (PPdK-RP2).

The levels of transcripts coding for some proteins typically associated with C_4_, however, were not increased in one, two or all three leaf stages. These exceptions include: beta carbonic anhydrase 3 (β-CA3), which shows higher transcript abundance in 1L and 2L, than in oL; triose phosphate translocator (TPT) mRNA levels, which are increased in 2L, but not in 1L or oL; transcripts encoding PPDK-RP1 are higher in 2L and oL, but not in 1L; PEPC-K1 mRNA abundance is higher in all leaf stages than in earlier developmental stages, but still very low in 1L; transcripts coding for PEPC-K2 are higher in 2L and oL, than in 1L; PPase6 transcript abundance is increased in 1L and 2L, but not in oL (Fig. 5, Supplementary Table S5).

Some transcripts gradually increase in abundance from stage 1L to stage 2L and peak in oL stage, for example, those encoding NHD2, PEPC-K1 and PEPC-K2, and PPDK-RP1 and PPDK-RP2. In contrast, transcripts encoding enzymes functioning in the NAD-malic enzyme (NAD-ME) or PEP-CK C_4_ subtypes such as the decarboxylation enzymes NAD-ME or PEP-CK did not show increased levels in leaves.

Taken together, these results indicate that most of the genes coding for known proteins of NADP-ME C_4_ subtype behave as predicted if C_3_ and C_4_ conducting tissues are compared (cotyledons and leaves of *S. soda*, respectively).

### The levels of most transcripts encoding proteins involved in photorespiration are not significantly decreased in leaves

The core photorespiratory cycle requires the eight enzymes (i) 2-phosphoglycolate phosphatase (PGLP), (ii) glycolate oxidase (GOX), (iii) glutamate:glyoxylate aminotransferase (GGT), (iv) the multienzyme glycine cleavage system (GDC) comprising three enzymes P-, T- and L-protein and the substrate H-protein, (v) serine hydroxymethyltransferase (SHMT), (vi) serine:glyoxylate aminotransferase (SGT), (vii) hydroxypyruvate reductase (HPR1) and (viii) glycerate 3-kinase (GLYK) (Bauwe *et al.*, 2010; Dellero *et al.*, 2016). Transcriptome and protein comparisons of C_3_ and C_4_ species found the components of photorespiration to be significantly higher in C_3_ than in C_4_ species (e.g. *Cleome*, Bräutigam *et al.*, 2011; *Flaveria*, Gowik *et al.*, 2011; Mallmann *et al.*, 2014). In contrast, in the C_4_ leaves of *S. soda*, the levels of transcripts coding for only two photorespiratory enzymes were significantly decreased compared with Cot: glycine decarboxylase P-Protein 2 and three isoforms of serine:glyoxylate aminotransferase (Fig. 5, Supplementary Table S5). Although the abundance of transcripts encoding most other photorespiratory proteins appears to decrease in leaves compared to Cot, these changes are not significant (Supplementary Table S5).

### K-means clustering reveals possible allies of known C_4_ pathway components

**Fig. 7.**
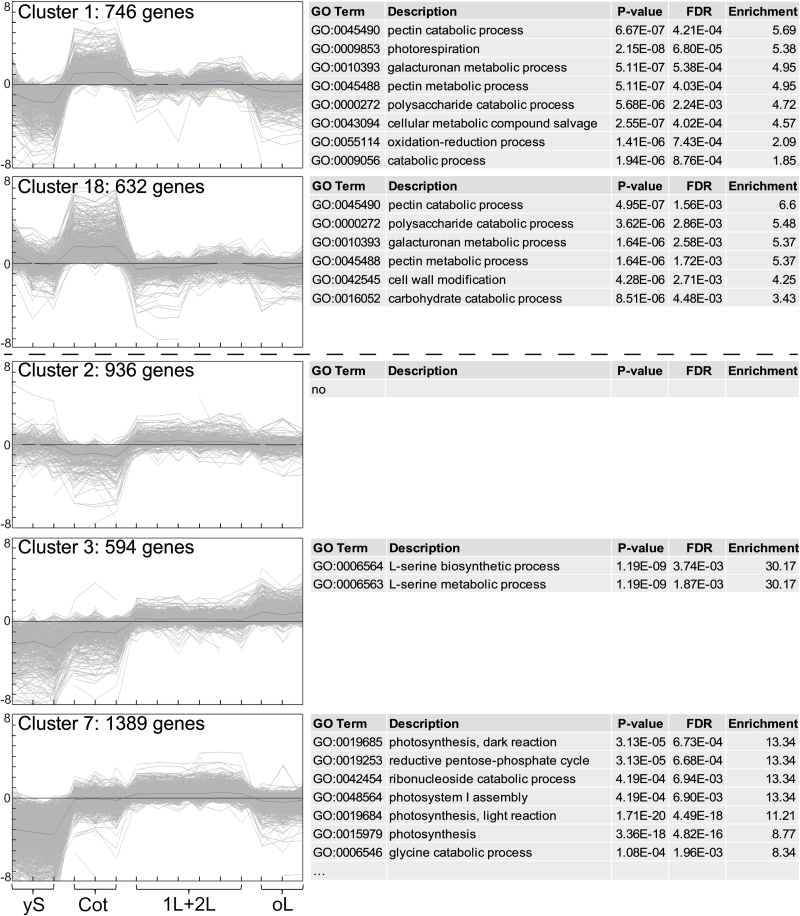
Five of 20 clusters from k-means analysis with C_4_ related expression pattern. All 20 clusters are shown in Supplementary Fig. S1.

To discover additional processes and genes that might be C_4_-related, transcriptome data of all five developmental stages of *S. soda* were clustered via K-means clustering. From a total of 20 clusters (Supplementary Fig. S1), some show no clear pattern (clusters 5, 9, 14, 15 and 16). Three clusters show a yS stage-related pattern (i.e. high expression in yS compared to other stages): cluster 8 enriched with GO processes ‘ribosomal small subunit biogenesis’, ‘ribonucleoprotein complex biogenesis’ and ‘translation’, cluster 11 enriched with GO processes ‘protein deneddylation’, ‘cullin deneddylation’ and ‘nucleocytoplasmic transport’ and cluster 20 with no enriched GO terms (Supplementary Table S6). Cluster 19 shows an oL stage-related pattern (i.e. high expression in oL compared to other stages), enriched in GO terms such as ‘cellular response to biotic stimulus’, ‘cellular response to molecule of bacterial origin’ and ‘programmed cell death’ (Supplementary Table S7). Five clusters show a possible C_4_-related pattern either with lower transcript levels in stages 1L and 2L compared to Cot (cluster 1 and 18) or higher levels in stages 1L and 2L compared to Cot (clusters 2, 3 and 7) (Fig. 7). Enriched GO processes in the Cot clusters 1 (including 746 genes) and 18 (including 632 genes) are related to cell wall synthesis and modification (e.g. ‘pectin catabolic process’, ‘galacturonan metabolic process’ and ‘pectin metabolic process’) and photorespiration (Supplementary Table S8). Cluster 2 (including 936 genes) shows no significantly enriched GO process. Cluster 3 (including 594 genes) shows two enriched GO terms, ‘L-serine metabolic process’ and ‘L-serine biosynthetic process’ (Fig. 7, Supplementary Table S9). Cluster 7 (including 1380 genes) shows many enriched GO terms, with the top five terms (ranked according to highest enrichment) related to photosynthesis, i.e. ‘photosynthesis, dark reaction’, ‘reductive pentose-phosphate cycle’, ‘ribonucleoside catabolic process’, ‘photosystem I assembly’ and ‘photosynthesis, light reaction’ (Supplementary Table S9).

Cluster 3 includes many genes encoding C_4_ cycle enzymes such as PEPC1 and PEPC2, NADP-MDH, Asp-AT5, NADP-ME isoforms 1, 2 and 4, Ala-AT1, PPdK, DiT2, BASS2 and BASS4, PPT1/CUE1 and PPT2, AMK2, and PPdK-RP1 and PPdK-RP2. Consequently, we carefully investigated the other genes included in cluster 3 and found two promising genes that code for transporters/transport-related proteins that might be involved in C_4_. The transcript encoding triose phosphate/phosphate translocator-related protein (TPT-related; AT1G43310) is significantly more abundant (log_2_FC between 2.4 and 2.8) in all leaf stages compared to Cot. Transcripts coding for two other proteins with transport activity are also significantly increased (log_2_FC between 1.6 and 2.6) in leaves compared to Cot: phosphate transporter4.1 (PHT4.1; AT2G29650) and PHT4.4 (AT4G00370), also known as anion transporter (ANTR) 1 and ANTR2, respectively.

Increased abundance of transcripts encoding pyruvate dehydrogenase kinase (PDH kinase; AT3G06483) is also significant (log_2_FC between 1.9 and 2.2) in all leaf stages compared to Cot. PDH kinase is thought to control metabolite exit from the C_4_ pathway via pyruvate decarboxylation for entry into citric acid cycle, and transcripts encoding the protein were increased in *Megathyrsus maximus*, a NAD-ME subtype C_4_ species (Bräutigam *et al.*, 2014). Our data indicate this is not to be limited to NAD-ME species, and PDH kinase may be required for the C_4_ pathway in general. Similarly, levels of mRNA coding for the enzyme asparagine synthetase 3 (ASN3; AT5G10240) are also significantly increased in all leaf stages compared to Cot (log_2_FC between 3.7 and 5.7).

To find possible candidates responsible for the regulatory mechanism(s) behind the transition from C_3_ to C_4_ in *S. soda* photosynthetic organs, we looked at the abundance of transcripts encoding transcription factors in the C_4_-related clusters 1, 2, 3, 7, and 18 (Supplementary Table S10). In total, mRNA levels of 87 transcription factors were either significantly decreased (32) or increased (55) between Cot and 1L plus 2L stages (Supplementary Table S10).

## Discussion

### Anatomy and carbon isotope ratios give first hints to C_3_ cotyledons and C_4_ leaves in *Salsola soda*

When we initially looked at cross sections of cotyledons and leaves of *S. soda*, we expected to find a salsoloid type C_4_ anatomy in both organs, since this was previously reported in the literature (Pyankov *et al.*, 2001*b*). In contrast, we found an undifferentiated C_3_ chlorenchyma surrounding a weakly developed central water storage tissue in cotyledons, while leaves showed salsoloid C_4_ anatomy typical of the C_4_-rich lineage Salsoleae to which *S. soda* belongs (Carolin *et al.*, 1975; Carraro *et al.*, 1993; Pyankov *et al.*, 2001*b*; Voznesenskaya *et al.*, 2013; Schüssler *et al.*, submitted).

Consistent with our anatomical findings, stable carbon isotope discrimination measurements of ten different developmental stages showed young cotyledons have a C_4_-like δ ^13^C even though no C_4_ pathway is functional. This is because they develop using carbon stored in the seed; carbon fixed by an adult plant via the C_4_ pathway. The dry seed probably has a more negative δ ^13^C value, compared to the imbibed seeds, because of its large lipid fraction, which was shown to have lower δ ^13^C than other fractions/parts of the plant (Park and Epstein, 1961). A C_3_-like δ ^13^C value cannot be measured in cotyledons until they are mature, when carbon assimilation is fully functional, and stored lipids are nearly depleted. The young first leaf pair, even if performing C_4_, has a C_3_ carbon isotope signature, since it was built with carbon fixed by cotyledon C_3_. A C_4_-like δ ^13^C value can be measured at the stage when the first leaf is fully expanded and mature. These results clearly show that the type of photosynthesis being carried out at a particular developmental stage is not necessarily reflected in concurrent carbon isotope measurements.

### *Salsola soda* developmental transcriptomes show stage-specific gene expression

Transcriptome sequencing of five different developmental stages of *S. soda* revealed insights into gene expression patterns of the different photosynthetic organs and/or stages including information on DGE of C_3_ and C_4_ in cotyledons and leaves. While the transcriptomes of the first and second leaves were very similar, all other stages were clearly distinct from each other (Fig. 3A, B). The young seedling stage was distinguished by transcripts encoding proteins involved in the glyoxylate cycle and mobilization of storage lipids, processes characteristic of seed germination and post-germination seedling stages. In cotyledons, transcripts coding for proteins of biosynthetic processes like pyruvate metabolism, glycolysis and photosynthesis were increased in abundance compared to young seedlings, indicating an active photoautotrophic mode of growth, mostly independent of lipids and proteins stored in the seed. While transcripts encoding RuBisCO were present in cotyledons, transcripts for all known enzymes and transporters of the C_4_ pathway were barely detectable. In contrast, the transcriptional programme of a C_4_ NADP-ME subtype was clearly represented in leaves. Importantly, we found that transcript abundance of the C_4_ key enzyme PEPC is consistent with PEPC protein amount. Thus, *S. soda* PEPC appears to be an example of where gene expression at the transcript and protein levels correlate (Ponnala *et al.*, 2014; Vélez-Bermúdez and Schmidt, 2014). Relative to the first and second leaf pairs, leaves of an adult plant demonstrated an increase in transcripts coding for proteins of biotic stress and stimulus response-related pathways. During the entire growth period, abiotic growth conditions were stable, and consequently, we can rule out possible abiotic stressors. Alternatively, increased abundance of transcripts encoding proteins involved in stimulus-related responses could be caused by the advanced age/maturity of oL stage; namely leaf senescence could have already been initiated. In Arabidopsis, leaf senescence can result in activation of signalling pathways also connected to stress responses (Lim *et al.*, 2007; Guo and Gan, 2012). During leaf senescence an activation of signalling pathways related to production of the plant hormones ABA, jasmonic acid, or salicylic acid and to programmed cell death was found (Morris *et al.*, 2000; Breeze *et al.*, 2011; Lim *et al.*, 2007). Interestingly, among the significantly enriched GO terms from the transcript pool of the oL stage were salicylic acid-mediated and also processes related to regulation of cell death (Supplementary Table S4).

Some C_4_ species such as maize, which have been historically considered as NADP-ME subtypes actually use two decarboxylation enzymes: NADP-ME, which produces CO_2_ and pyruvate, and PEP-CK, which decarboxylates oxaloacetate, releasing CO_2_ and PEP (Walker *et al.*, 1997; Pick *et al.*, 2011; Wang *et al.*, 2014). Furbank (2011) hypothesized that mixed decarboxylation pathways could be present in many C_4_ plants characterized as NADP-ME subtype. In *S. soda*, however, all three leaf stages had only high levels of NADP-ME transcripts. An increased abundance of PEP-CK transcripts was found in cotyledons, where the enzyme functions as part of gluconeogenesis. Taken together, our results suggest that *S. soda* exclusively uses NADP-ME as the primary decarboxylase. This is supported by results of enzyme assays and western blots of PEP-CK that also revealed NADP-ME-only decarboxylation in *S. soda* (Koteyeva *et al.*, 2015), and is in agreement with the situation in bona fide C_4_*Flaveria* species and *Sorghum bicolor* (Gowik *et al.*, 2011; Döring *et al.*, 2016) that also rely only on NADP-ME. Interestingly, it was recently reported that the Salsoleae species *H. ammodendron* not only relies on the NADP-ME pathway, but additionally performs NAD-ME type C_4_ (Li *et al.*, 2015), indicating the capability to develop different types of C_4_ within the Salsoleae.

Photorespiration is thought to be lower in C_4_ plants than C_3_ species, because of the CO_2_ concentration mechanism and the accompanying decrease in RuBisCO oxygenase activity. Comparisons of C_3_ with C_4_ species showed a significant decrease in transcripts coding for photorespiratory proteins as well as a decrease in the actual protein abundance in the C_4_ species (Mallmann *et al.*, 2014). We see the same tendency for most transcripts encoding photorespiratory proteins in *S. soda* although most of these changes are not significant, with the exception of transcripts encoding a GDC P-protein and three serine:glyoxylate aminotransferases (cluster 1, Fig. 7, Supplemental Table S5). A similar tendency was observed when the transcriptomes of cotyledons and assimilating shoots of *H. ammodendron* were compared—the vast majority of photorespiratory genes were down-regulated only moderately in the C_4_ compared to the C_3_ tissue (Li *et al.*, 2015). An explanation of these findings in *S. soda* could be that, at the time of sampling, the cotyledons were not fully developed, resulting in a lower photosynthetic and hence a lower photorespiratory activity. However, the C_3_-like δ ^13^C value of mature cotyledons as well as the levels of RubisCO and other photosynthetic transcripts indicate active C_3_ at this stage (Figs 2, 4).

### New possible C_4_ allies

Leaf transcriptomes from closely related species performing different types of photosynthetic biochemistry have greatly expanded our knowledge of the proteins needed for the efficient functioning of C_4_ (e.g. Bräutigam *et al.*, 2011; Gowik *et al.*, 2011; van den Bergh *et al.*, 2014; Külahoglu *et al.*, 2014; Ding *et al.*, 2015; Aubry *et al.*, 2016). However, gaps remain in our understanding of these processes. In the current study, we identified novel transcripts that could be related to the C_4_ pathway. These transcripts showed similar expression patterns to those coding for known C_4_ enzymes, C_4_-related transporters and C_4_-related regulatory proteins, i.e. they are more or less absent in C_3_ cotyledons, but highly abundant in C_4_ leaves.

Newly proposed C_4_ candidates include two proteins of the PHT4 family, PHT4.1 and PHT4.4, which were thought to be involved in the transport of inorganic phosphate between the cytosol and chloroplasts (Roth *et al.*, 2004; Guo *et al.*, 2008; Linka and Weber, 2010). Recently, however, Miyaji and colleagues (2015) showed that PHT4.4 functions as an ascorbate transporter in Arabidopsis, moving ascorbate from the cytosol into the stroma of chloroplast. They further proposed that PHT4.1 transports ascorbate from the stroma into the thylakoids, hence the transporters work together to control the dynamic state of ascorbate in chloroplasts. This is important as ascorbic acid is a non-enzymatic component that scavenges reactive oxygen species (ROS) (Yamane *et al.*, 2012), and high ascorbate levels are required to overcome photoinhibition/photostress (Miyaji *et al.*, 2015). It has been shown that some C_4_ plants of the NADP-ME type have a low PSII:PSI ratio in BS thylakoids, which mostly conduct cyclic electron transport, while M thylakoids exhibit a complete linear electron transport chain (Meierhoff and Westhoff, 1993; reviewed in Majeran and van Wijk, 2009). The presence of linear electron transfer in the M and the concurrent absence of most photorespiratory reactions in this compartment (Kubicki *et al.*, 1996; Döring *et al.*, 2016) could lead to the production of ROS in the M especially under stress conditions (Miller *et al.*, 2010). Apart from photorespiration, protection against ROS could be accomplished by antioxidant components like ascorbate, enriched in M chloroplasts by means of PHT4.4 and PHT4.1. While it is tempting to project a role for PHT4.4 and PHT4.1 in protection against photostress in the M cells of NADP-ME C_4_ species, further analysis is necessary to elucidate the significance of these transporters for the C_4_ metabolism, interestingly, PHT4.4 was found enriched in M chloroplast envelopes of the C_4_ plant maize (Majeran and van Wijk, 2009).

Additionally, we found a transcript of a TPT-related protein significantly more abundant in leaf stages compared to Cot. The TPT has been shown to be involved in C_4_ transport processes exchanging 3-phosphoglycerate and triose phosphate between BS and M chloroplasts (Flügge, 1999; Bräutigam *et al.*, 2008). However, whether the TPT-related protein is involved in the regulation of TPT or is a transport protein by itself needs to be further investigated.

Another gene showing a C_4_-like expression pattern in *S. soda* encodes the asparagine synthetase ASN3. In general, asparagine synthetases function in ammonium metabolism in the cytosol of leaf and root cells. Asparagine is a key compound for nitrogen transport and storage, because of its high nitrogen:carbon ratio (Lam *et al.*, 1996). It is well known that C_4_ plants have a better nitrogen use efficiency compared to C_3_ plants due to their reduced need for Calvin-Benson cycle and photorespiratory enzymes (Long, 1999). It was shown that this leads to a reduction of the protein synthesis machinery as well to the amino acid and overall nitrogen metabolism in the leaves of C_4_ plants (Bräutigam *et al.*, 2011; Gowik *et al.*, 2011). The changes in expression of genes related to asparagine and phosphoserine metabolism observed in our transcriptome analysis might be connected to adaptations in nitrogen metabolism after the switch from C_3_ to C_4_.

While some *cis*-regulatory elements involved in the expression of the core C_4_ genes were identified in the past (Marshall *et al.*, 1997, Gowik *et al.*, 2004, Williams *et al.*, 2016), our knowledge of C_4_ transcription factors is very limited. Since the co-expression analysis found all core C_4_ genes in one common cluster (cluster 3, Fig. 7), it is tempting to consider the transcription factors found in this cluster to be putative regulators of these C_4_ genes. Interestingly, several homeobox domain genes were found in cluster 3 and homeobox domain proteins were identified previously as potential regulators of the C_4_ PEPC gene in *Flaveria* (Windhövel *et al.*, 2001).

### Why C_3_ in cotyledons in the first place?

Our analyses clearly show that cotyledons of *S. soda* perform C_3_ while a switch to the C_4_ pathway occurs in leaves. This transition, observable in the same individual plant and enabled by the same genome, makes *S. soda* an excellent candidate to study the molecular developmental changes underlying the switch. A fundamental question is: why does *S. soda* use different pathways in the two photosynthetically active organs? The embryo within the seed of *S. soda* is very large (ML, personal observation), suggesting that cotyledons do not contribute to carbon assimilation and energy gain in *S. soda* at all, and the occurrence of C_3_ in cotyledons is an evolutionary relict. However, carbon isotope data refute this suggestion in that cotyledons assimilate carbon via C_3_ to such an extent that a C_3_-like δ^13^C signature is detected in the young first leaf (Fig. 2). Furthermore, we observed that cotyledons are long-lived organs in *S. soda*, appearing to be still photosynthetically active when the first leaf pair reaches the mature stage.

Our rationale for C_3_ metabolism in cotyledons of *S. soda* is that C_3_ provides a selective advantage through lower metabolic costs than C_4_. *Salsola soda* is found at the shores around the Mediterranean Sea to Central Asia (Freitag, 1997), and seeds of *S. soda* germinate in spring, in temperate environmental conditions, i.e. moderate temperature and precipitation, compared to the rest of the annual growing period. Under these conditions the costs of C_3_, even with the associated photorespiratory activity, are lower than operating a C_4_ cycle (Ehleringer *et al.*, 1991). Additionally, low or moderate temperatures result in a lower quantum yield in plants using C_4_ compared to C_3_ metabolism (Ehleringer, 1978). Hence *S. soda* conducts C_3_ in cotyledons because C_3_ outperforms C_4_ in the environmental conditions present at germination and during the first days/weeks of the seedling stages, whereas C_4_ becomes environmentally advantageous from the first leaf stage onwards, when temperatures and light intensity are higher. The results of the current study certainly support this hypothesis. Comparative developmental studies, including monitoring environmental conditions, between *S. soda* and closely related species in which both cotyledons and leaves use either C_3_ or C_4_ would further resolve the potential advantage(s) of the C_3_ to C_4_ transition demonstrated by *S. soda*.

In summary, we were able to provide evidence that *S. soda* conducts C_3_ in cotyledons before it switches to C_4_ in leaves. *Salsola soda* has ideal features to be an additional model plant for studying the genetic background and the biochemical assembly of setting up the C_4_ pathway: an annual life cycle, easy and fast to grow in the greenhouse, production of many seeds per plant each year, seeds with a high germination rate (in the first year), and the switch from C_3_ to C_4_ occurs within a single species and individual (i.e. identical genome).

## Supplementary data

Supplementary data are available at *JXB* online.

Figure S1. K-means clustering (K=20 clusters) of all transcripts in all five stages. 

Table S1. List of carbon isotope values of ten stages including technical triplicates (see Fig. 2).

Table S2. Data availability of raw short read data of all samples uploaded to National Center for Biotechnology Information Sequence Read Archive.

Table S3. Mapping of reads, transcript abundance and statistical tests of differential gene expression.

Table S4. Enriched GO (gene ontology) terms highly represented in one stage (first column) compared to a certain other stage (second column).

Table S5. Expression pattern of genes involved in glyoxylate cycle, photosynthesis, photorespiration and C_4_ photosynthesis in the five developmental stages.

Table S6. Enriched GO (gene ontology) terms in yS-related clusters 8 and 11.

Table S7. Enriched GO (gene ontology) terms in oL-related cluster 19.

Table S8. Enriched GO (gene ontology) terms in Cot-related clusters 1 and 18 (see Fig. 7).

Table S9. Enriched GO (gene ontology) terms in C_4_-related clusters 3 and 7 (see Fig. 7).

Table S10. Transcriptions factors (TF) included in k-means clusters 1, 2, 3, 7, and 18 that show decreased (clusters 1 and 18) or increased transcript levels (clusters 2, 3 and 7) in 1L and 2L compared to Cot (FDR q-value ≤0.01). 

## Supplementary Material

Supplementary_Figure_S1

Supplementary_Table_S1

Supplementary_Table_S2

Supplementary_Table_S3

Supplementary_Table_S4

Supplementary_Table_S5

Supplementary_Table_S6

Supplementary_Table_S7

Supplementary_Table_S8

Supplementary_Table_S9

Supplementary_Table_S10
