# Supplementary figures and images for: C3 cotyledons are followed by C4 leaves: intra-individual transcriptome analysis of Salsola soda (Chenopodiaceae)
Source: J Exp Bot. 2016 Sep 22;68(2):161–76. doi: 10.1093/jxb/erw343 (PMC5853821; doi:10.1093/jxb/erw343)

Supplementary Figure S1.

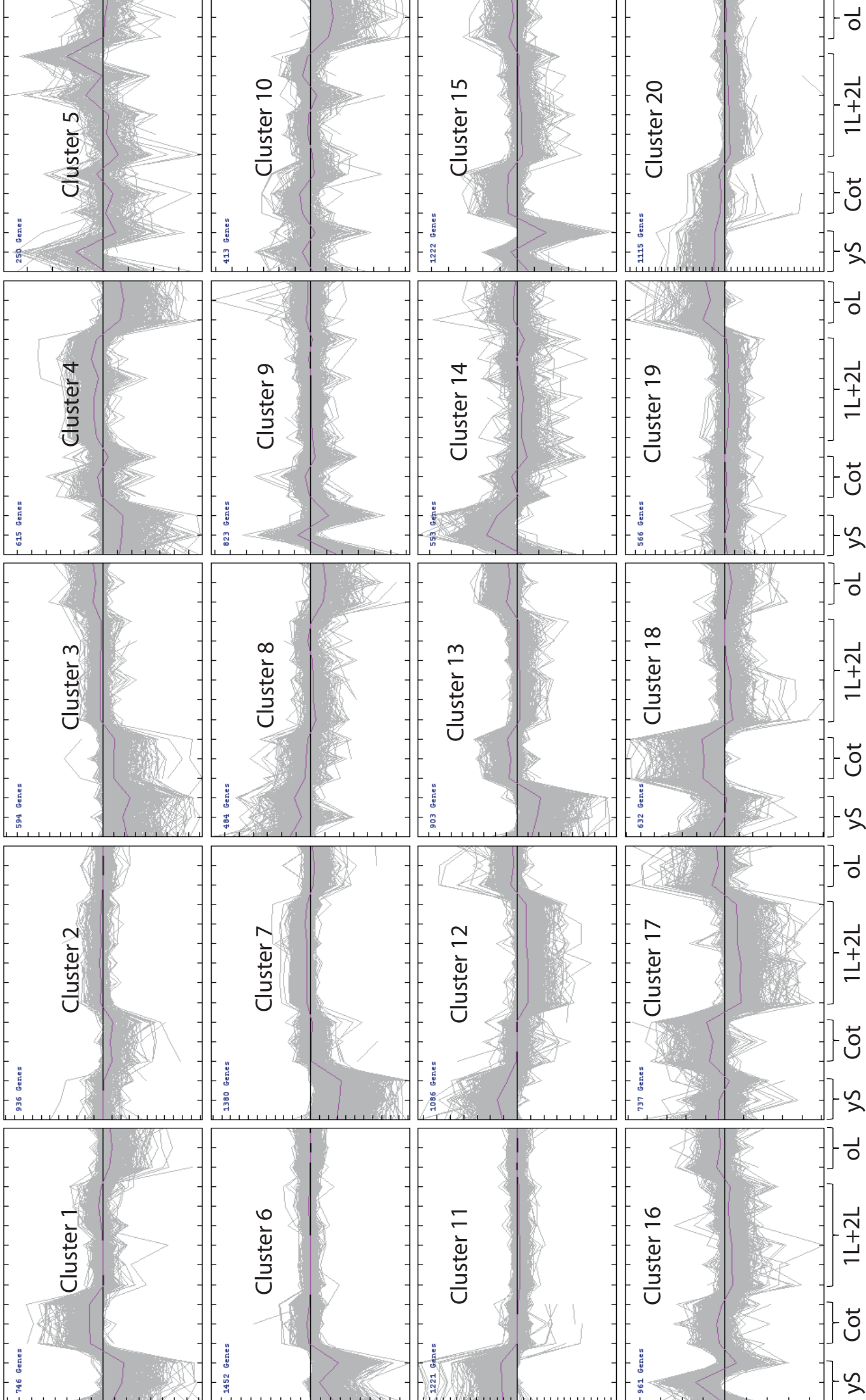

Supplement: Supplementary_Figure_S1 [file erw343_suppl_supplementary_figure_s1.pdf]
