# Supplementary material for: C3 cotyledons are followed by C4 leaves: intra-individual transcriptome analysis of Salsola soda (Chenopodiaceae)
Source: J Exp Bot. 2016 Sep 22;68(2):161–76. doi: 10.1093/jxb/erw343 (PMC5853821; doi:10.1093/jxb/erw343)
Supplement: Supplementary_Table_S1 [file erw343_suppl_supplementary_table_s1.pdf]

Supplementary Table S1.

Stage	mean $\delta^{13}C$ (‰)	Standard deviation
Seed, dry	-19.128	0.0402
Seed, imbibed	-15.826	0.08
Cotyledons, young	-17.341	0.1719
Cotyledons, mature	-22.367	0.6437
1st leaf pair, young	-22.387	0.1765
1st leaf pair, mature	-17.385	2.4631
2nd leaf pair, young	-16.889	0.0918
2nd leaf pair, mature	-15.434	0.1315
3rd leaf pair, mature	-15.699	0.1023
Leaf pair, adult plant	-15.866	0.2031
