# Supplementary material for: C3 cotyledons are followed by C4 leaves: intra-individual transcriptome analysis of Salsola soda (Chenopodiaceae)
Source: J Exp Bot. 2016 Sep 22;68(2):161–76. doi: 10.1093/jxb/erw343 (PMC5853821; doi:10.1093/jxb/erw343)
Supplement: Supplementary_Table_S2 [file erw343_suppl_supplementary_table_s2.pdf]

Supplementary Table S2.

Developmental Stage	ID	Accession number
Young seedling	yS_1	SRX1774515
	yS_2	SRX1774640
	yS_3	SRX1774643
Cotyledon	Cot_1	SRX1774539
	Cot_2	SRX1774648
	Cot_3	SRX1774661
First leaf pair	1L_1	SRX1774540
	1L_2	SRX1774663
	1L_3	SRX1774664
Second leaf pair	2L_1	SRX1774600
	2L_2	SRX1774665
	2L_3	SRX1774666
Old leaf pair	oL_1	SRX1774608
	oL_2	SRX1774667
	oL_3	SRX1774668
