# Supplementary material for: C3 cotyledons are followed by C4 leaves: intra-individual transcriptome analysis of Salsola soda (Chenopodiaceae)
Source: J Exp Bot. 2016 Sep 22;68(2):161–76. doi: 10.1093/jxb/erw343 (PMC5853821; doi:10.1093/jxb/erw343)
Supplement: Supplementary_Table_S4 [file erw343_suppl_supplementary_table_s4.pdf]

Stage	Up-regulated compared to:	Enriched GO term	P-value	FDR	Description
yS	Cot	GO:0006913	2.11E-13	6.47E-10	nucleocytoplasmic transport
		GO:0051169	2.11E-13	3.24E-10	nuclear transport
		GO:0034504	7.30E-12	7.48E-09	protein localization to nucleus
		GO:0006606	7.30E-12	5.61E-09	protein import into nucleus
		GO:1902593	7.30E-12	4.49E-09	single-organism nuclear import
		GO:0051170	7.30E-12	3.74E-09	nuclear import
		GO:0051028	1.29E-08	5.68E-06	mRNA transport
		GO:0050658	5.96E-08	2.29E-05	RNA transport
		GO:0050657	5.96E-08	2.03E-05	nucleic acid transport
		GO:0051236	5.96E-08	1.83E-05	establishment of RNA localization
		GO:0017038	6.66E-08	1.86E-05	protein import
		GO:0009628	1.24E-07	3.16E-05	response to abiotic stimulus
		GO:0051168	1.48E-07	3.49E-05	nuclear export
		GO:0009791	2.04E-07	4.47E-05	post-embryonic development
		GO:0006406	2.19E-07	4.48E-05	mRNA export from nucleus
		GO:0006413	2.34E-07	4.49E-05	translational initiation
		GO:0033365	9.61E-07	1.74E-04	protein localization to organelle
		GO:0072594	9.61E-07	1.64E-04	establishment of protein localization to organelle
		GO:0009640	1.16E-06	1.87E-04	photomorphogenesis
		GO:0006405	1.46E-06	2.25E-04	RNA export from nucleus
		GO:0016482	1.59E-06	2.32E-04	cytoplasmic transport
		GO:0022414	2.48E-06	3.46E-04	reproductive process
		GO:0019915	3.54E-06	4.73E-04	lipid storage
		GO:0032501	4.27E-06	5.46E-04	multicellular organismal process
		GO:0009314	7.55E-06	9.27E-04	response to radiation
		GO:0000338	9.14E-06	1.08E-03	protein deneddylation
		GO:0010388	9.14E-06	1.04E-03	cullin deneddylation
		GO:0044707	1.11E-05	1.22E-03	single-multicellular organism process
		GO:0070647	1.19E-05	1.26E-03	protein modification by small protein conjugation or removal
		GO:0009560	1.24E-05	1.26E-03	embryo sac egg cell differentiation
		GO:0009416	1.30E-05	1.28E-03	response to light stimulus
		GO:1902580	1.30E-05	1.25E-03	single-organism cellular localization
		GO:0034613	2.70E-05	2.51E-03	cellular protein localization
		GO:0070727	2.70E-05	2.44E-03	cellular macromolecule localization
		GO:0009639	2.76E-05	2.42E-03	response to red or far red light
		GO:0015931	4.12E-05	3.52E-03	nucleobase-containing compound transport
		GO:0003006	4.17E-05	3.46E-03	developmental process involved in reproduction
		GO:0008380	4.19E-05	3.39E-03	RNA splicing
		GO:0009751	5.04E-05	3.97E-03	response to salicylic acid
		GO:1902582	5.97E-05	4.58E-03	single-organism intracellular transport
		GO:0022412	6.03E-05	4.52E-03	cellular process involved in reproduction in multicellular organism
		GO:0006626	8.88E-05	6.49E-03	protein targeting to mitochondrion
		GO:0031323	9.86E-05	7.04E-03	regulation of cellular metabolic process
		GO:0016567	1.22E-04	8.53E-03	protein ubiquitination
		GO:0009266	1.46E-04	9.96E-03	response to temperature stimulus
	1L	GO:0006413	6.98E-10	2.14E-06	translational initiation
		GO:0006913	5.25E-08	8.06E-05	nucleocytoplasmic transport

		GO:0051169	5.25E-08	5.37E-05	nuclear transport
		GO:0051028	2.43E-07	1.86E-04	mRNA transport
		GO:0070647	5.01E-07	3.08E-04	protein modification by small protein conjugation or removal
		GO:0009628	8.91E-07	4.56E-04	response to abiotic stimulus
		GO:0050658	1.03E-06	4.52E-04	RNA transport
		GO:0050657	1.03E-06	3.95E-04	nucleic acid transport
		GO:0051236	1.03E-06	3.51E-04	establishment of RNA localization
		GO:0034504	1.03E-06	3.16E-04	protein localization to nucleus
		GO:0006606	1.03E-06	2.87E-04	protein import into nucleus
		GO:1902593	1.03E-06	2.63E-04	single-organism nuclear import
		GO:0051170	1.03E-06	2.43E-04	nuclear import
		GO:0010200	2.51E-06	5.51E-04	response to chitin
		GO:0010243	2.51E-06	5.15E-04	response to organonitrogen compound
		GO:0032446	3.32E-06	6.37E-04	protein modification by small protein conjugation
		GO:0009791	3.34E-06	6.03E-04	post-embryonic development
		GO:0051168	9.33E-06	1.59E-03	nuclear export
		GO:0006406	1.31E-05	2.12E-03	mRNA export from nucleus
		GO:0019915	1.50E-05	2.30E-03	lipid storage
		GO:0016567	1.53E-05	2.24E-03	protein ubiquitination
		GO:0009408	1.99E-05	2.78E-03	response to heat
		GO:0031146	4.79E-05	6.39E-03	SCF-dependent proteasomal ubiquitin-dependent protein catabolic process
		GO:0006397	5.29E-05	6.76E-03	mRNA processing
		GO:0044265	5.29E-05	6.49E-03	cellular macromolecule catabolic process
		GO:0008380	5.60E-05	6.61E-03	RNA splicing
		GO:1901698	5.80E-05	6.60E-03	response to nitrogen compound
		GO:0009640	5.84E-05	6.40E-03	photomorphogenesis
		GO:0006405	6.31E-05	6.68E-03	RNA export from nucleus
		GO:0022414	7.36E-05	7.54E-03	reproductive process
		GO:0009314	9.59E-05	9.50E-03	response to radiation
		GO:0090487	1.04E-04	1.00E-02	secondary metabolite catabolic process
		GO:0009407	1.04E-04	9.71E-03	toxin catabolic process
		GO:0009404	1.04E-04	9.42E-03	toxin metabolic process
		GO:0032501	1.09E-04	9.53E-03	multicellular organismal process
	2L	GO:0006413	1.11E-09	3.42E-06	translational initiation
		GO:0010200	1.43E-08	2.19E-05	response to chitin
		GO:0010243	4.43E-08	1.46E-05	response to organonitrogen compound
		GO:1901698	5.52E-06	1.17E-03	response to nitrogen compound
		GO:0009628	3.31E-06	2.04E-03	response to abiotic stimulus
		GO:1901700	7.31E-06	3.74E-03	response to oxygen-containing compound
		GO:0009791	1.49E-05	6.52E-03	post-embryonic development
		GO:0019915	1.81E-05	6.93E-03	lipid storage
	oL	GO:0022613	9.05E-07	2.78E-03	ribonucleoprotein complex biogenesis
Cot	yS	GO:0019682	4.20E-23	1.29E-19	glyceraldehyde-3-phosphate metabolic process
		GO:0006081	4.89E-18	7.51E-15	cellular aldehyde metabolic process
		GO:0044710	2.18E-17	2.23E-14	single-organism metabolic process
		GO:0006629	3.24E-17	2.49E-14	lipid metabolic process
		GO:0019288	9.31E-16	5.72E-13	isopentenyl diphosphate biosynthetic process, methylerythritol 4-phosphate pathway
		GO:0009240	9.31E-16	4.77E-13	isopentenyl diphosphate biosynthetic process

GO:0046490	9.31E-16	4.08E-13	isopentenyl diphosphate metabolic process
GO:0008610	1.94E-15	7.43E-13	lipid biosynthetic process
GO:0044711	2.67E-15	9.11E-13	single-organism biosynthetic process
GO:0044699	9.08E-14	2.79E-11	single-organism process
GO:0015979	9.17E-14	2.56E-11	photosynthesis
GO:0009657	7.48E-13	1.91E-10	plastid organization
GO:0010027	1.35E-12	3.20E-10	thylakoid membrane organization
GO:0009668	1.35E-12	2.97E-10	plastid membrane organization
GO:0044255	1.83E-12	3.74E-10	cellular lipid metabolic process
GO:0051186	2.87E-12	5.50E-10	cofactor metabolic process
GO:0044763	7.04E-12	1.27E-09	single-organism cellular process
GO:0006098	8.67E-12	1.48E-09	pentose-phosphate shunt
GO:0051156	8.67E-12	1.40E-09	glucose 6-phosphate metabolic process
GO:0044281	8.90E-12	1.37E-09	small molecule metabolic process
GO:0006739	1.76E-11	2.58E-09	NADP metabolic process
GO:0019684	2.80E-11	3.91E-09	photosynthesis, light reaction
GO:0010207	4.00E-11	5.35E-09	photosystem II assembly
GO:0043436	8.38E-11	1.07E-08	oxoacid metabolic process
GO:0006082	8.38E-11	1.03E-08	organic acid metabolic process
GO:0042440	1.74E-10	2.05E-08	pigment metabolic process
GO:0006090	2.32E-10	2.64E-08	pyruvate metabolic process
GO:0006644	4.51E-10	4.95E-08	phospholipid metabolic process
GO:0006733	4.83E-10	5.12E-08	oxidoreduction coenzyme metabolic process
GO:0019752	5.02E-10	5.14E-08	carboxylic acid metabolic process
GO:0016109	2.00E-09	1.98E-07	tetraterpenoid biosynthetic process
GO:0016108	2.00E-09	1.92E-07	tetraterpenoid metabolic process
GO:0016116	2.00E-09	1.86E-07	carotenoid metabolic process
GO:0016117	2.00E-09	1.80E-07	carotenoid biosynthetic process
GO:0006732	2.24E-09	1.96E-07	coenzyme metabolic process
GO:0046148	2.63E-09	2.24E-07	pigment biosynthetic process
GO:0006793	2.72E-09	2.26E-07	phosphorus metabolic process
GO:0006520	2.85E-09	2.30E-07	cellular amino acid metabolic process
GO:0008654	3.04E-09	2.39E-07	phospholipid biosynthetic process
GO:0009658	3.64E-09	2.80E-07	chloroplast organization
GO:0044283	5.55E-09	4.15E-07	small molecule biosynthetic process
GO:0006796	6.61E-09	4.83E-07	phosphate-containing compound metabolic process
GO:0006790	7.23E-09	5.16E-07	sulfur compound metabolic process
GO:0044802	7.23E-09	5.04E-07	single-organism membrane organization
GO:0006720	8.57E-09	5.85E-07	isoprenoid metabolic process
GO:0008299	1.17E-08	7.81E-07	isoprenoid biosynthetic process
GO:0009653	1.29E-08	8.44E-07	anatomical structure morphogenesis
GO:1901605	2.03E-08	1.30E-06	alpha-amino acid metabolic process
GO:0000023	2.21E-08	1.38E-06	maltose metabolic process
GO:0009902	2.23E-08	1.37E-06	chloroplast relocation
GO:0051644	2.23E-08	1.34E-06	plastid localization
GO:0051667	2.23E-08	1.32E-06	establishment of plastid localization
GO:0019750	2.23E-08	1.29E-06	chloroplast localization
GO:1901135	2.67E-08	1.52E-06	carbohydrate derivative metabolic process
GO:0006721	2.95E-08	1.65E-06	terpenoid metabolic process
GO:0005982	3.10E-08	1.70E-06	starch metabolic process

GO:0005984	3.48E-08	1.87E-06	disaccharide metabolic process
GO:0016114	4.01E-08	2.12E-06	terpenoid biosynthetic process
GO:0009893	4.83E-08	2.51E-06	positive regulation of metabolic process
GO:0005976	6.39E-08	3.27E-06	polysaccharide metabolic process
GO:0072524	8.34E-08	4.20E-06	pyridine-containing compound metabolic process
GO:0019252	1.14E-07	5.63E-06	starch biosynthetic process
GO:0019362	1.15E-07	5.61E-06	pyridine nucleotide metabolic process
GO:0046496	1.15E-07	5.52E-06	nicotinamide nucleotide metabolic process
GO:0044272	1.25E-07	5.92E-06	sulfur compound biosynthetic process
GO:0006534	1.81E-07	8.41E-06	cysteine metabolic process
GO:0008652	2.41E-07	1.10E-05	cellular amino acid biosynthetic process
GO:0019344	2.43E-07	1.10E-05	cysteine biosynthetic process
GO:0016556	2.89E-07	1.29E-05	mRNA modification
GO:0044093	3.17E-07	1.39E-05	positive regulation of molecular function
GO:0019637	5.74E-07	2.48E-05	organophosphate metabolic process
GO:0006066	6.15E-07	2.62E-05	alcohol metabolic process
GO:0043085	7.49E-07	3.15E-05	positive regulation of catalytic activity
GO:0009311	7.51E-07	3.12E-05	oligosaccharide metabolic process
GO:0010103	8.06E-07	3.30E-05	stomatal complex morphogenesis
GO:0090626	8.06E-07	3.26E-05	plant epidermis morphogenesis
GO:0000096	1.02E-06	4.06E-05	sulfur amino acid metabolic process
GO:0009965	1.03E-06	4.01E-05	leaf morphogenesis
GO:0051656	1.03E-06	3.96E-05	establishment of organelle localization
GO:0010075	1.03E-06	4.05E-05	regulation of meristem growth
GO:0008152	1.11E-06	4.21E-05	metabolic process
GO:0048518	1.33E-06	5.00E-05	positive regulation of biological process
GO:0009069	1.52E-06	5.63E-05	serine family amino acid metabolic process
GO:0046165	1.86E-06	6.80E-05	alcohol biosynthetic process
GO:0000097	1.94E-06	7.01E-05	sulfur amino acid biosynthetic process
GO:0055114	2.08E-06	7.42E-05	oxidation-reduction process
GO:0009637	2.22E-06	7.83E-05	response to blue light
GO:0009886	2.30E-06	8.01E-05	post-embryonic morphogenesis
GO:0009070	2.41E-06	8.31E-05	serine family amino acid biosynthetic process
GO:0015994	5.56E-06	1.90E-04	chlorophyll metabolic process
GO:0065008	5.78E-06	1.95E-04	regulation of biological quality
GO:1901617	6.28E-06	2.10E-04	organic hydroxy compound biosynthetic process
GO:0007169	8.53E-06	2.82E-04	transmembrane receptor protein tyrosine kinase signaling pathway
GO:0007167	8.53E-06	2.79E-04	enzyme linked receptor protein signaling pathway
GO:0061024	8.82E-06	2.85E-04	membrane organization
GO:0006778	9.46E-06	3.03E-04	porphyrin-containing compound metabolic process
GO:0033013	9.46E-06	2.99E-04	tetrapyrrole metabolic process
GO:0006073	9.48E-06	2.97E-04	cellular glucan metabolic process
GO:0044042	9.48E-06	2.94E-04	glucan metabolic process
GO:0071554	9.86E-06	3.03E-04	cell wall organization or biogenesis
GO:0048509	1.19E-05	3.60E-04	regulation of meristem development
GO:1901607	1.25E-05	3.75E-04	alpha-amino acid biosynthetic process
GO:0016053	1.27E-05	3.78E-04	organic acid biosynthetic process
GO:0046394	1.27E-05	3.75E-04	carboxylic acid biosynthetic process
GO:0006811	1.40E-05	4.08E-04	ion transport

GO:0009250	1.44E-05	4.18E-04	glucan biosynthetic process
GO:0000272	1.47E-05	4.21E-04	polysaccharide catabolic process
GO:0032787	1.62E-05	4.60E-04	monocarboxylic acid metabolic process
GO:0000271	1.90E-05	5.36E-04	polysaccharide biosynthetic process
GO:0010264	2.13E-05	5.94E-04	myo-inositol hexakisphosphate biosynthetic process
GO:0033517	2.13E-05	5.89E-04	myo-inositol hexakisphosphate metabolic process
GO:0042793	2.36E-05	6.46E-04	transcription from plastid promoter
GO:0051188	2.48E-05	6.75E-04	cofactor biosynthetic process
GO:1903825	2.78E-05	7.49E-04	organic acid transmembrane transport
GO:0042742	2.80E-05	7.48E-04	defense response to bacterium
GO:1901615	2.80E-05	7.42E-04	organic hydroxy compound metabolic process
GO:0009411	3.12E-05	8.18E-04	response to UV
GO:0042744	3.19E-05	8.29E-04	hydrogen peroxide catabolic process
GO:0007166	3.30E-05	8.53E-04	cell surface receptor signaling pathway
GO:0048878	3.53E-05	9.04E-04	chemical homeostasis
GO:0015995	3.61E-05	9.17E-04	chlorophyll biosynthetic process
GO:0035303	3.61E-05	9.09E-04	regulation of dephosphorylation
GO:0035304	3.61E-05	9.02E-04	regulation of protein dephosphorylation
GO:0032958	4.19E-05	1.04E-03	inositol phosphate biosynthetic process
GO:0009854	4.89E-05	1.20E-03	oxidative photosynthetic carbon pathway
GO:0046890	4.89E-05	1.19E-03	regulation of lipid biosynthetic process
GO:0005975	4.90E-05	1.18E-03	carbohydrate metabolic process
GO:0071555	5.66E-05	1.36E-03	cell wall organization
GO:0055080	6.34E-05	1.51E-03	cation homeostasis
GO:0050801	6.73E-05	1.59E-03	ion homeostasis
GO:0010817	6.93E-05	1.62E-03	regulation of hormone levels
GO:0044712	6.94E-05	1.61E-03	single-organism catabolic process
GO:0006091	1.02E-04	2.37E-03	generation of precursor metabolites and energy
GO:0030001	1.04E-04	2.37E-03	metal ion transport
GO:0009617	1.11E-04	2.52E-03	response to bacterium
GO:0044550	1.11E-04	2.50E-03	secondary metabolite biosynthetic process
GO:0006873	1.17E-04	2.62E-03	cellular ion homeostasis
GO:0030003	1.17E-04	2.60E-03	cellular cation homeostasis
GO:0051640	1.20E-04	2.65E-03	organelle localization
GO:1901565	1.23E-04	2.69E-03	organonitrogen compound catabolic process
GO:0006779	1.25E-04	2.72E-03	porphyrin-containing compound biosynthetic process
GO:0033014	1.25E-04	2.70E-03	tetrapyrrole biosynthetic process
GO:0072593	1.28E-04	2.74E-03	reactive oxygen species metabolic process
GO:0046173	1.40E-04	3.00E-03	polyol biosynthetic process
GO:0040008	1.62E-04	3.44E-03	regulation of growth
GO:0055082	1.68E-04	3.54E-03	cellular chemical homeostasis
GO:0009072	1.73E-04	3.62E-03	aromatic amino acid family metabolic process
GO:0016143	1.74E-04	3.60E-03	S-glycoside metabolic process
GO:0019760	1.74E-04	3.58E-03	glucosinolate metabolic process
GO:0019757	1.74E-04	3.55E-03	glycosinolate metabolic process
GO:0042743	1.76E-04	3.59E-03	hydrogen peroxide metabolic process
GO:0044264	2.56E-04	5.17E-03	cellular polysaccharide metabolic process
GO:0010015	2.58E-04	5.17E-03	root morphogenesis
GO:0016144	3.35E-04	6.68E-03	S-glycoside biosynthetic process
GO:0019758	3.35E-04	6.64E-03	glycosinolate biosynthetic process

		GO:0019761	3.35E-04	6.59E-03	glucosinolate biosynthetic process
		GO:0034637	3.58E-04	7.00E-03	cellular carbohydrate biosynthetic process
		GO:0045490	4.56E-04	8.86E-03	pectin catabolic process
	1L	GO:0009755	1.66E-06	5.11E-03	hormone-mediated signaling pathway
		GO:0055114	5.87E-06	9.02E-03	oxidation-reduction process
		GO:2000377	7.37E-06	7.55E-03	regulation of reactive oxygen species metabolic process
	2L	GO:0055114	5.08E-08	1.56E-04	oxidation-reduction process
		GO:0009723	3.74E-07	5.75E-04	response to ethylene
		GO:1901700	5.63E-07	5.76E-04	response to oxygen-containing compound
		GO:0042221	2.98E-06	2.29E-03	response to chemical
		GO:0001101	3.18E-06	1.96E-03	response to acid chemical
		GO:1901698	3.47E-06	1.78E-03	response to nitrogen compound
		GO:2000377	9.81E-06	4.30E-03	regulation of reactive oxygen species metabolic process
		GO:0010035	1.95E-05	7.49E-03	response to inorganic substance
	oL	GO:0045229	1.87E-12	5.74E-09	external encapsulating structure organization
		GO:0071555	2.59E-12	3.98E-09	cell wall organization
		GO:0045490	3.53E-12	3.61E-09	pectin catabolic process
		GO:0071554	9.24E-12	7.09E-09	cell wall organization or biogenesis
		GO:0000272	6.55E-11	4.03E-08	polysaccharide catabolic process
		GO:0010393	7.01E-10	3.59E-07	galacturonan metabolic process
		GO:0045488	7.01E-10	3.07E-07	pectin metabolic process
		GO:0016042	3.96E-06	1.52E-03	lipid catabolic process
		GO:0042545	5.43E-06	1.85E-03	cell wall modification
		GO:0010162	8.81E-06	2.70E-03	seed dormancy process
		GO:0022611	8.81E-06	2.46E-03	dormancy process
		GO:0007165	1.28E-05	3.27E-03	signal transduction
		GO:0048453	1.75E-05	4.12E-03	sepal formation
		GO:0048451	1.75E-05	3.83E-03	petal formation
		GO:0048609	1.75E-05	3.57E-03	multicellular organismal reproductive process
		GO:0007017	2.68E-05	5.14E-03	microtubule-based process
		GO:0008283	3.85E-05	6.96E-03	cell proliferation
1L	yS	GO:0019682	2.05E-32	6.29E-29	glyceraldehyde-3-phosphate metabolic process
		GO:0006081	9.42E-27	1.45E-23	cellular aldehyde metabolic process
		GO:0019288	2.68E-24	2.74E-21	isopentenyl diphosphate biosynthetic process, methylerythritol 4-phosphate pathway
		GO:0009240	2.68E-24	2.06E-21	isopentenyl diphosphate biosynthetic process
		GO:0046490	2.68E-24	1.65E-21	isopentenyl diphosphate metabolic process
		GO:0015979	4.28E-19	2.19E-16	photosynthesis
		GO:0009657	5.78E-19	2.54E-16	plastid organization
		GO:0006629	6.76E-19	2.60E-16	lipid metabolic process
		GO:0044710	6.58E-17	2.25E-14	single-organism metabolic process
		GO:0010027	2.59E-16	7.94E-14	thylakoid membrane organization
		GO:0009668	2.59E-16	7.22E-14	plastid membrane organization
		GO:0008610	6.18E-16	1.58E-13	lipid biosynthetic process
		GO:0043436	1.12E-15	2.65E-13	oxoacid metabolic process
		GO:0006082	1.12E-15	2.46E-13	organic acid metabolic process
		GO:0006090	1.63E-15	3.34E-13	pyruvate metabolic process
		GO:0051186	1.33E-14	2.56E-12	cofactor metabolic process
		GO:0019752	1.34E-14	2.41E-12	carboxylic acid metabolic process
		GO:0044711	1.44E-14	2.46E-12	single-organism biosynthetic process

GO:0009658	1.48E-14	2.39E-12	chloroplast organization
GO:0010207	2.05E-13	3.15E-11	photosystem II assembly
GO:0006098	2.09E-13	3.05E-11	pentose-phosphate shunt
GO:0051156	2.09E-13	2.91E-11	glucose 6-phosphate metabolic process
GO:0044699	2.87E-13	3.83E-11	single-organism process
GO:0006644	3.59E-13	4.60E-11	phospholipid metabolic process
GO:0006739	4.74E-13	5.82E-11	NADP metabolic process
GO:0042440	9.21E-13	1.09E-10	pigment metabolic process
GO:1901135	1.27E-12	1.45E-10	carbohydrate derivative metabolic process
GO:0044281	2.54E-12	2.79E-10	small molecule metabolic process
GO:0016556	2.58E-12	2.73E-10	mRNA modification
GO:0005984	2.77E-12	2.83E-10	disaccharide metabolic process
GO:0019684	2.95E-12	2.93E-10	photosynthesis, light reaction
GO:0016109	3.41E-12	3.28E-10	tetraterpenoid biosynthetic process
GO:0016108	3.41E-12	3.18E-10	tetraterpenoid metabolic process
GO:0016116	3.41E-12	3.08E-10	carotenoid metabolic process
GO:0016117	3.41E-12	2.99E-10	carotenoid biosynthetic process
GO:0046148	4.71E-12	4.02E-10	pigment biosynthetic process
GO:0008654	6.64E-12	5.51E-10	phospholipid biosynthetic process
GO:0044255	6.77E-12	5.47E-10	cellular lipid metabolic process
GO:0006520	1.45E-11	1.14E-09	cellular amino acid metabolic process
GO:1901605	1.99E-11	1.53E-09	alpha-amino acid metabolic process
GO:0044763	2.33E-11	1.74E-09	single-organism cellular process
GO:0009653	3.05E-11	2.23E-09	anatomical structure morphogenesis
GO:0005982	3.35E-11	2.39E-09	starch metabolic process
GO:0000023	1.14E-10	7.98E-09	maltose metabolic process
GO:0006732	1.32E-10	9.04E-09	coenzyme metabolic process
GO:0008652	2.16E-10	1.44E-08	cellular amino acid biosynthetic process
GO:0006790	2.64E-10	1.72E-08	sulfur compound metabolic process
GO:0019252	4.26E-10	2.72E-08	starch biosynthetic process
GO:0009070	5.47E-10	3.43E-08	serine family amino acid biosynthetic process
GO:0009069	6.30E-10	3.87E-08	serine family amino acid metabolic process
GO:0006534	6.56E-10	3.95E-08	cysteine metabolic process
GO:0019344	7.79E-10	4.60E-08	cysteine biosynthetic process
GO:0006733	1.05E-09	6.11E-08	oxidoreduction coenzyme metabolic process
GO:0009311	1.42E-09	8.07E-08	oligosaccharide metabolic process
GO:0044802	2.22E-09	1.24E-07	single-organism membrane organization
GO:0009902	4.53E-09	2.49E-07	chloroplast relocation
GO:0051644	4.53E-09	2.44E-07	plastid localization
GO:0051667	4.53E-09	2.40E-07	establishment of plastid localization
GO:0019750	4.53E-09	2.36E-07	chloroplast localization
GO:0008152	6.32E-09	3.23E-07	metabolic process
GO:0009893	9.49E-09	4.78E-07	positive regulation of metabolic process
GO:0044272	1.03E-08	5.08E-07	sulfur compound biosynthetic process
GO:0016053	1.29E-08	6.29E-07	organic acid biosynthetic process
GO:0046394	1.29E-08	6.19E-07	carboxylic acid biosynthetic process
GO:0044283	1.53E-08	7.24E-07	small molecule biosynthetic process
GO:0006793	2.80E-08	1.30E-06	phosphorus metabolic process
GO:1901607	2.94E-08	1.35E-06	alpha-amino acid biosynthetic process
GO:0006720	3.03E-08	1.37E-06	isoprenoid metabolic process

GO:0032787	3.61E-08	1.61E-06	monocarboxylic acid metabolic process
GO:0008299	4.02E-08	1.76E-06	isoprenoid biosynthetic process
GO:0072524	4.92E-08	2.13E-06	pyridine-containing compound metabolic process
GO:0006796	6.02E-08	2.57E-06	phosphate-containing compound metabolic process
GO:0019362	6.61E-08	2.78E-06	pyridine nucleotide metabolic process
GO:0046496	6.61E-08	2.74E-06	nicotinamide nucleotide metabolic process
GO:0010103	1.04E-07	4.27E-06	stomatal complex morphogenesis
GO:0090626	1.04E-07	4.22E-06	plant epidermis morphogenesis
GO:0000096	1.48E-07	5.91E-06	sulfur amino acid metabolic process
GO:0009886	1.55E-07	6.11E-06	post-embryonic morphogenesis
GO:0006778	1.62E-07	6.28E-06	porphyrin-containing compound metabolic process
GO:0033013	1.62E-07	6.20E-06	tetrapyrrole metabolic process
GO:0009637	1.81E-07	6.88E-06	response to blue light
GO:0019637	1.98E-07	7.42E-06	organophosphate metabolic process
GO:0051188	2.35E-07	8.69E-06	cofactor biosynthetic process
GO:0000097	2.68E-07	9.81E-06	sulfur amino acid biosynthetic process
GO:0009965	3.31E-07	1.20E-05	leaf morphogenesis
GO:0051656	3.31E-07	1.18E-05	establishment of organelle localization
GO:0006721	3.67E-07	1.30E-05	terpenoid metabolic process
GO:0042793	3.91E-07	1.36E-05	transcription from plastid promoter
GO:0006073	4.14E-07	1.43E-05	cellular glucan metabolic process
GO:0044042	4.14E-07	1.41E-05	glucan metabolic process
GO:0045036	4.51E-07	1.52E-05	protein targeting to chloroplast
GO:0072596	4.51E-07	1.50E-05	establishment of protein localization to chloroplast
GO:0072598	4.51E-07	1.49E-05	protein localization to chloroplast
GO:0016114	4.89E-07	1.60E-05	terpenoid biosynthetic process
GO:1901564	7.77E-07	2.51E-05	organonitrogen compound metabolic process
GO:0044093	8.63E-07	2.76E-05	positive regulation of molecular function
GO:0015994	8.63E-07	2.73E-05	chlorophyll metabolic process
GO:0009250	1.02E-06	3.19E-05	glucan biosynthetic process
GO:0005976	1.03E-06	3.20E-05	polysaccharide metabolic process
GO:0007169	1.82E-06	5.60E-05	transmembrane receptor protein tyrosine kinase signaling pathway
GO:0007167	1.82E-06	5.55E-05	enzyme linked receptor protein signaling pathway
GO:0043085	1.96E-06	5.90E-05	positive regulation of catalytic activity
GO:0006779	2.87E-06	8.54E-05	porphyrin-containing compound biosynthetic process
GO:0033014	2.87E-06	8.46E-05	tetrapyrrole biosynthetic process
GO:0007166	3.04E-06	8.90E-05	cell surface receptor signaling pathway
GO:0042742	3.34E-06	9.66E-05	defense response to bacterium
GO:0005975	4.06E-06	1.17E-04	carbohydrate metabolic process
GO:0071555	4.85E-06	1.38E-04	cell wall organization
GO:0071554	5.33E-06	1.50E-04	cell wall organization or biogenesis
GO:1901565	8.49E-06	2.37E-04	organonitrogen compound catabolic process
GO:0048518	9.03E-06	2.50E-04	positive regulation of biological process
GO:0010075	9.66E-06	2.65E-04	regulation of meristem growth
GO:0015995	1.00E-05	2.73E-04	chlorophyll biosynthetic process
GO:0008361	1.23E-05	3.31E-04	regulation of cell size
GO:0044712	1.47E-05	3.92E-04	single-organism catabolic process
GO:0044262	1.49E-05	3.95E-04	cellular carbohydrate metabolic process
GO:0009617	2.46E-05	6.45E-04	response to bacterium

GO:0006091	2.54E-05	6.61E-04	generation of precursor metabolites and energy
GO:0048509	2.82E-05	7.28E-04	regulation of meristem development
GO:0000271	3.92E-05	1.00E-03	polysaccharide biosynthetic process
GO:0061024	4.25E-05	1.08E-03	membrane organization
GO:0034637	5.04E-05	1.27E-03	cellular carbohydrate biosynthetic process
GO:0031328	5.33E-05	1.33E-03	positive regulation of cellular biosynthetic process
GO:0035303	6.17E-05	1.53E-03	regulation of dephosphorylation
GO:0035304	6.17E-05	1.52E-03	regulation of protein dephosphorylation
GO:0051640	6.91E-05	1.68E-03	organelle localization
GO:0045229	6.92E-05	1.67E-03	external encapsulating structure organization
GO:0006873	8.67E-05	2.08E-03	cellular ion homeostasis
GO:0030003	8.67E-05	2.06E-03	cellular cation homeostasis
GO:0055114	9.05E-05	2.14E-03	oxidation-reduction process
GO:0009891	1.01E-04	2.37E-03	positive regulation of biosynthetic process
GO:0071704	1.04E-04	2.41E-03	organic substance metabolic process
GO:0009073	1.05E-04	2.42E-03	aromatic amino acid family biosynthetic process
GO:0055082	1.28E-04	2.92E-03	cellular chemical homeostasis
GO:0031325	1.36E-04	3.10E-03	positive regulation of cellular metabolic process
GO:0009072	1.37E-04	3.10E-03	aromatic amino acid family metabolic process
GO:0055080	1.46E-04	3.26E-03	cation homeostasis
GO:0044264	1.52E-04	3.38E-03	cellular polysaccharide metabolic process
GO:1902680	1.65E-04	3.65E-03	positive regulation of RNA biosynthetic process
GO:0045893	1.65E-04	3.62E-03	positive regulation of transcription, DNA-templated
GO:1903508	1.65E-04	3.60E-03	positive regulation of nucleic acid-templated transcription
GO:0016226	1.69E-04	3.66E-03	iron-sulfur cluster assembly
GO:0031163	1.69E-04	3.63E-03	metallo-sulfur cluster assembly
GO:0065008	1.85E-04	3.95E-03	regulation of biological quality
GO:0042744	2.14E-04	4.53E-03	hydrogen peroxide catabolic process
GO:0051254	2.27E-04	4.77E-03	positive regulation of RNA metabolic process
GO:0048878	2.27E-04	4.74E-03	chemical homeostasis
GO:0045935	2.31E-04	4.80E-03	positive regulation of nucleobase-containing compound metabolic process
GO:0044550	2.33E-04	4.79E-03	secondary metabolite biosynthetic process
GO:0009411	2.45E-04	5.02E-03	response to UV
GO:0044767	2.77E-04	5.64E-03	single-organism developmental process
GO:0016143	3.08E-04	6.22E-03	S-glycoside metabolic process
GO:0019760	3.08E-04	6.18E-03	glucosinolate metabolic process
GO:0019757	3.08E-04	6.14E-03	glycosinolate metabolic process
GO:0019725	3.13E-04	6.19E-03	cellular homeostasis
GO:0090407	3.53E-04	6.95E-03	organophosphate biosynthetic process
GO:0000272	3.56E-04	6.96E-03	polysaccharide catabolic process
GO:0016043	3.70E-04	7.18E-03	cellular component organization
GO:0010015	3.75E-04	7.24E-03	root morphogenesis
GO:0050801	4.04E-04	7.75E-03	ion homeostasis
GO:0009767	4.10E-04	7.83E-03	photosynthetic electron transport chain
GO:0010817	4.14E-04	7.84E-03	regulation of hormone levels
GO:0010557	4.16E-04	7.83E-03	positive regulation of macromolecule biosynthetic process
GO:1903825	4.27E-04	7.99E-03	organic acid transmembrane transport
GO:0000038	4.27E-04	7.95E-03	very long-chain fatty acid metabolic process

	GO:0009733	4.82E-04	8.92E-03	response to auxin
	GO:0033692	4.85E-04	8.93E-03	cellular polysaccharide biosynthetic process
	GO:0051173	5.47E-04	9.99E-03	positive regulation of nitrogen compound metabolic process
	GO:0010628	5.50E-04	9.99E-03	positive regulation of gene expression
	GO:0019761	5.61E-04	1.00E-02	glucosinolate biosynthetic process
Cot	GO:0016556	2.81E-08	8.63E-05	mRNA modification
	GO:0009653	6.62E-08	1.02E-04	anatomical structure morphogenesis
	GO:0005984	2.99E-07	3.07E-04	disaccharide metabolic process
	GO:0019682	1.01E-06	7.74E-04	glyceraldehyde-3-phosphate metabolic process
	GO:0044550	1.23E-06	7.53E-04	secondary metabolite biosynthetic process
	GO:0010103	1.92E-06	9.83E-04	stomatal complex morphogenesis
	GO:0090626	1.92E-06	8.42E-04	plant epidermis morphogenesis
	GO:0009311	3.77E-06	1.45E-03	oligosaccharide metabolic process
	GO:0007169	4.16E-06	1.42E-03	transmembrane receptor protein tyrosine kinase signaling pathway
	GO:0007167	4.16E-06	1.28E-03	enzyme linked receptor protein signaling pathway
	GO:0019748	6.18E-06	1.73E-03	secondary metabolic process
	GO:0019288	7.69E-06	1.97E-03	isopentenyl diphosphate biosynthetic process, methylerythritol 4-phosphate pathway
	GO:0009240	7.69E-06	1.82E-03	isopentenyl diphosphate biosynthetic process
	GO:0046490	7.69E-06	1.69E-03	isopentenyl diphosphate metabolic process
	GO:0006081	7.85E-06	1.61E-03	cellular aldehyde metabolic process
	GO:0009657	9.25E-06	1.77E-03	plastid organization
	GO:0009893	1.04E-05	1.89E-03	positive regulation of metabolic process
	GO:0010557	1.11E-05	1.89E-03	positive regulation of macromolecule biosynthetic process
	GO:0046351	1.32E-05	2.03E-03	disaccharide biosynthetic process
	GO:0010628	1.32E-05	2.14E-03	positive regulation of gene expression
	GO:1902680	1.57E-05	2.29E-03	positive regulation of RNA biosynthetic process
	GO:0045893	1.57E-05	2.19E-03	positive regulation of transcription, DNA-templated
	GO:1903508	1.57E-05	2.09E-03	positive regulation of nucleic acid-templated transcription
	GO:0051254	1.88E-05	2.41E-03	positive regulation of RNA metabolic process
	GO:0051173	2.21E-05	2.71E-03	positive regulation of nitrogen compound metabolic process
	GO:0031328	2.60E-05	3.07E-03	positive regulation of cellular biosynthetic process
	GO:0007166	2.66E-05	3.02E-03	cell surface receptor signaling pathway
	GO:0009891	2.72E-05	2.99E-03	positive regulation of biosynthetic process
	GO:0071555	2.88E-05	3.05E-03	cell wall organization
	GO:0045935	3.18E-05	3.25E-03	positive regulation of nucleobase-containing compound metabolic process
	GO:0044802	7.48E-05	7.41E-03	single-organism membrane organization
	GO:0010604	7.60E-05	7.29E-03	positive regulation of macromolecule metabolic process
	GO:0009886	9.24E-05	8.60E-03	post-embryonic morphogenesis
	GO:0045229	9.50E-05	8.58E-03	external encapsulating structure organization
	GO:0071554	9.60E-05	8.42E-03	cell wall organization or biogenesis
2L	none	---	---	---
oL	GO:0071555	6.29E-18	1.93E-14	cell wall organization
	GO:0045229	1.92E-16	2.94E-13	external encapsulating structure organization
	GO:0071554	4.39E-16	4.50E-13	cell wall organization or biogenesis
	GO:0007169	2.70E-10	2.07E-07	transmembrane receptor protein tyrosine kinase signaling pathway

GO:0007167	2.70E-10	1.66E-07	enzyme linked receptor protein signaling pathway
GO:0007166	6.95E-09	3.56E-06	cell surface receptor signaling pathway
GO:0000272	9.75E-09	4.28E-06	polysaccharide catabolic process
GO:0019288	2.05E-08	7.85E-06	isopentenyl diphosphate biosynthetic process, methylerythritol 4-phosphate pathway
GO:0009240	2.05E-08	6.98E-06	isopentenyl diphosphate biosynthetic process
GO:0046490	2.05E-08	6.28E-06	isopentenyl diphosphate metabolic process
GO:0019682	4.14E-08	1.16E-05	glyceraldehyde-3-phosphate metabolic process
GO:0045490	8.03E-08	2.06E-05	pectin catabolic process
GO:0048509	1.87E-07	4.42E-05	regulation of meristem development
GO:0010075	2.05E-07	4.50E-05	regulation of meristem growth
GO:0010103	4.31E-07	8.83E-05	stomatal complex morphogenesis
GO:0010393	4.31E-07	8.28E-05	galacturonan metabolic process
GO:0045488	4.31E-07	7.79E-05	pectin metabolic process
GO:0090626	4.31E-07	7.36E-05	plant epidermis morphogenesis
GO:0009653	4.95E-07	8.00E-05	anatomical structure morphogenesis
GO:0005975	1.16E-06	1.78E-04	carbohydrate metabolic process
GO:2000026	2.95E-06	4.31E-04	regulation of multicellular organismal development
GO:0009886	3.04E-06	4.24E-04	post-embryonic morphogenesis
GO:0008361	3.05E-06	4.07E-04	regulation of cell size
GO:0048453	3.29E-06	4.21E-04	sepal formation
GO:0048451	3.29E-06	4.04E-04	petal formation
GO:0051239	4.68E-06	5.52E-04	regulation of multicellular organismal process
GO:0000911	5.73E-06	6.51E-04	cytokinesis by cell plate formation
GO:0048449	7.77E-06	8.52E-04	floral organ formation
GO:0006629	7.96E-06	8.43E-04	lipid metabolic process
GO:0040008	9.23E-06	9.45E-04	regulation of growth
GO:0032506	9.85E-06	9.76E-04	cytokinetic process
GO:1902410	9.85E-06	9.46E-04	mitotic cytokinetic process
GO:0010027	1.29E-05	1.20E-03	thylakoid membrane organization
GO:0009668	1.29E-05	1.17E-03	plastid membrane organization
GO:0006081	1.31E-05	1.15E-03	cellular aldehyde metabolic process
GO:0044767	1.53E-05	1.31E-03	single-organism developmental process
GO:0007017	1.67E-05	1.39E-03	microtubule-based process
GO:0016043	2.21E-05	1.79E-03	cellular component organization
GO:0016042	2.67E-05	2.10E-03	lipid catabolic process
GO:0071840	3.20E-05	2.46E-03	cellular component organization or biogenesis
GO:1903047	3.33E-05	2.49E-03	mitotic cell cycle process
GO:0006090	3.41E-05	2.49E-03	pyruvate metabolic process
GO:0008283	5.64E-05	4.03E-03	cell proliferation
GO:0009664	7.34E-05	5.12E-03	plant-type cell wall organization
GO:0032502	7.54E-05	5.14E-03	developmental process
GO:0048638	7.93E-05	5.29E-03	regulation of developmental growth
GO:0010015	7.98E-05	5.21E-03	root morphogenesis
GO:0015995	8.10E-05	5.18E-03	chlorophyll biosynthetic process
GO:0050793	8.61E-05	5.40E-03	regulation of developmental process
GO:0048653	1.11E-04	6.79E-03	anther development
GO:0008152	1.45E-04	8.73E-03	metabolic process
GO:0000226	1.55E-04	9.18E-03	microtubule cytoskeleton organization
GO:0071669	1.62E-04	9.39E-03	plant-type cell wall organization or biogenesis

		GO:0009926	1.70E-04	9.68E-03	auxin polar transport
2L	yS	GO:0019682	9.51E-33	2.92E-29	glyceraldehyde-3-phosphate metabolic process
		GO:0006081	4.22E-27	6.48E-24	cellular aldehyde metabolic process
		GO:0019288	1.59E-24	1.63E-21	isopentenyl diphosphate biosynthetic process, methylerythritol 4-phosphate pathway
		GO:0009240	1.59E-24	1.22E-21	isopentenyl diphosphate biosynthetic process
		GO:0046490	1.59E-24	9.77E-22	isopentenyl diphosphate metabolic process
		GO:0006629	3.65E-20	1.87E-17	lipid metabolic process
		GO:0044710	1.05E-19	4.60E-17	single-organism metabolic process
		GO:0015979	3.18E-19	1.22E-16	photosynthesis
		GO:0009657	2.59E-18	8.84E-16	plastid organization
		GO:0044711	7.02E-18	2.16E-15	single-organism biosynthetic process
		GO:0010027	1.78E-16	4.97E-14	thylakoid membrane organization
		GO:0009668	1.78E-16	4.56E-14	plastid membrane organization
		GO:0043436	3.59E-16	8.48E-14	oxoacid metabolic process
		GO:0006082	3.59E-16	7.88E-14	organic acid metabolic process
		GO:0006090	9.55E-16	1.96E-13	pyruvate metabolic process
		GO:0008610	1.95E-15	3.73E-13	lipid biosynthetic process
		GO:0019752	2.21E-15	3.99E-13	carboxylic acid metabolic process
		GO:0051186	6.87E-15	1.17E-12	cofactor metabolic process
		GO:0006098	2.23E-14	3.60E-12	pentose-phosphate shunt
		GO:0051156	2.23E-14	3.42E-12	glucose 6-phosphate metabolic process
		GO:0006739	5.30E-14	7.76E-12	NADP metabolic process
		GO:0006644	6.45E-14	9.00E-12	phospholipid metabolic process
		GO:0009658	7.30E-14	9.75E-12	chloroplast organization
		GO:0042440	1.34E-13	1.72E-11	pigment metabolic process
		GO:0010207	1.54E-13	1.89E-11	photosystem II assembly
		GO:0006520	1.64E-13	1.93E-11	cellular amino acid metabolic process
		GO:0005982	1.99E-13	2.26E-11	starch metabolic process
		GO:0044281	2.40E-13	2.64E-11	small molecule metabolic process
		GO:0000023	3.15E-13	3.34E-11	maltose metabolic process
		GO:0008652	4.21E-13	4.31E-11	cellular amino acid biosynthetic process
		GO:0046148	6.01E-13	5.95E-11	pigment biosynthetic process
		GO:0044699	7.83E-13	7.52E-11	single-organism process
		GO:0044763	9.05E-13	8.42E-11	single-organism cellular process
		GO:0008654	1.28E-12	1.16E-10	phospholipid biosynthetic process
		GO:0044255	1.43E-12	1.26E-10	cellular lipid metabolic process
		GO:0016556	1.88E-12	1.60E-10	mRNA modification
		GO:0005984	1.97E-12	1.64E-10	disaccharide metabolic process
		GO:0019684	2.41E-12	1.95E-10	photosynthesis, light reaction
		GO:0019252	2.66E-12	2.10E-10	starch biosynthetic process
		GO:0016109	2.71E-12	2.08E-10	tetraterpenoid biosynthetic process
		GO:0016108	2.71E-12	2.03E-10	tetraterpenoid metabolic process
		GO:0016116	2.71E-12	1.98E-10	carotenoid metabolic process
		GO:0016117	2.71E-12	1.94E-10	carotenoid biosynthetic process
		GO:1901605	3.98E-12	2.78E-10	alpha-amino acid metabolic process
		GO:0009653	6.45E-12	4.40E-10	anatomical structure morphogenesis
		GO:0009069	1.43E-11	9.55E-10	serine family amino acid metabolic process
		GO:0006732	2.85E-11	1.86E-09	coenzyme metabolic process
		GO:1901135	6.52E-11	4.17E-09	carbohydrate derivative metabolic process

GO:0009070	6.66E-11	4.17E-09	serine family amino acid biosynthetic process
GO:0006733	7.12E-11	4.38E-09	oxidoreduction coenzyme metabolic process
GO:0006790	1.70E-10	1.02E-08	sulfur compound metabolic process
GO:0009311	2.50E-10	1.48E-08	oligosaccharide metabolic process
GO:0044283	5.52E-10	3.20E-08	small molecule biosynthetic process
GO:0016053	6.81E-10	3.87E-08	organic acid biosynthetic process
GO:0046394	6.81E-10	3.80E-08	carboxylic acid biosynthetic process
GO:1901607	7.66E-10	4.20E-08	alpha-amino acid biosynthetic process
GO:0005976	1.24E-09	6.68E-08	polysaccharide metabolic process
GO:0009893	2.40E-09	1.27E-07	positive regulation of metabolic process
GO:0006534	3.05E-09	1.59E-07	cysteine metabolic process
GO:0019344	3.75E-09	1.92E-07	cysteine biosynthetic process
GO:0009902	3.80E-09	1.91E-07	chloroplast relocation
GO:0051644	3.80E-09	1.88E-07	plastid localization
GO:0051667	3.80E-09	1.85E-07	establishment of plastid localization
GO:0019750	3.80E-09	1.82E-07	chloroplast localization
GO:0044802	4.12E-09	1.95E-07	single-organism membrane organization
GO:0044272	7.10E-09	3.31E-07	sulfur compound biosynthetic process
GO:0044093	8.04E-09	3.68E-07	positive regulation of molecular function
GO:0009250	9.08E-09	4.10E-07	glucan biosynthetic process
GO:0072524	1.19E-08	5.30E-07	pyridine-containing compound metabolic process
GO:0006073	1.41E-08	6.20E-07	cellular glucan metabolic process
GO:0044042	1.41E-08	6.12E-07	glucan metabolic process
GO:0019362	1.61E-08	6.87E-07	pyridine nucleotide metabolic process
GO:0046496	1.61E-08	6.78E-07	nicotinamide nucleotide metabolic process
GO:0008152	1.66E-08	6.91E-07	metabolic process
GO:0010103	1.67E-08	6.83E-07	stomatal complex morphogenesis
GO:0090626	1.67E-08	6.74E-07	plant epidermis morphogenesis
GO:0043085	2.01E-08	8.03E-07	positive regulation of catalytic activity
GO:0006720	2.32E-08	9.15E-07	isoprenoid metabolic process
GO:0008299	3.10E-08	1.20E-06	isoprenoid biosynthetic process
GO:0009886	3.10E-08	1.19E-06	post-embryonic morphogenesis
GO:0000096	3.49E-08	1.32E-06	sulfur amino acid metabolic process
GO:0032787	3.75E-08	1.40E-06	monocarboxylic acid metabolic process
GO:0000271	4.91E-08	1.82E-06	polysaccharide biosynthetic process
GO:0000097	6.40E-08	2.34E-06	sulfur amino acid biosynthetic process
GO:0019637	1.07E-07	3.86E-06	organophosphate metabolic process
GO:0009965	2.81E-07	1.00E-05	leaf morphogenesis
GO:0051656	2.81E-07	9.92E-06	establishment of organelle localization
GO:0006721	2.92E-07	1.02E-05	terpenoid metabolic process
GO:0042793	3.44E-07	1.19E-05	transcription from plastid promoter
GO:0016114	3.91E-07	1.33E-05	terpenoid biosynthetic process
GO:0006793	4.24E-07	1.43E-05	phosphorus metabolic process
GO:0006778	4.91E-07	1.64E-05	porphyrin-containing compound metabolic process
GO:0033013	4.91E-07	1.62E-05	tetrapyrrole metabolic process
GO:0051188	6.27E-07	2.05E-05	cofactor biosynthetic process
GO:0044712	6.48E-07	2.10E-05	single-organism catabolic process
GO:0015994	7.05E-07	2.26E-05	chlorophyll metabolic process
GO:0044264	7.95E-07	2.52E-05	cellular polysaccharide metabolic process
GO:0034637	8.51E-07	2.67E-05	cellular carbohydrate biosynthetic process

GO:0033692	1.15E-06	3.55E-05	cellular polysaccharide biosynthetic process
GO:0044262	1.31E-06	4.02E-05	cellular carbohydrate metabolic process
GO:0006796	1.34E-06	4.09E-05	phosphate-containing compound metabolic process
GO:0005975	1.35E-06	4.07E-05	carbohydrate metabolic process
GO:0071554	1.77E-06	5.27E-05	cell wall organization or biogenesis
GO:1901564	1.81E-06	5.35E-05	organonitrogen compound metabolic process
GO:0010075	2.15E-06	6.28E-05	regulation of meristem growth
GO:0045036	3.10E-06	8.97E-05	protein targeting to chloroplast
GO:0072596	3.10E-06	8.89E-05	establishment of protein localization to chloroplast
GO:0072598	3.10E-06	8.81E-05	protein localization to chloroplast
GO:0009637	3.25E-06	9.15E-05	response to blue light
GO:0009072	3.86E-06	1.08E-04	aromatic amino acid family metabolic process
GO:0009073	3.93E-06	1.09E-04	aromatic amino acid family biosynthetic process
GO:0048509	7.07E-06	1.94E-04	regulation of meristem development
GO:0015995	8.86E-06	2.41E-04	chlorophyll biosynthetic process
GO:0008361	1.12E-05	3.02E-04	regulation of cell size
GO:0048518	1.13E-05	3.03E-04	positive regulation of biological process
GO:0006779	1.19E-05	3.14E-04	porphyrin-containing compound biosynthetic process
GO:0033014	1.19E-05	3.11E-04	tetrapyrrole biosynthetic process
GO:0042742	1.23E-05	3.20E-04	defense response to bacterium
GO:0098656	1.55E-05	4.01E-04	anion transmembrane transport
GO:0071555	1.72E-05	4.41E-04	cell wall organization
GO:0042744	1.76E-05	4.46E-04	hydrogen peroxide catabolic process
GO:0006091	1.98E-05	4.98E-04	generation of precursor metabolites and energy
GO:0061024	3.07E-05	7.66E-04	membrane organization
GO:0000038	3.81E-05	9.44E-04	very long-chain fatty acid metabolic process
GO:0016226	3.85E-05	9.46E-04	iron-sulfur cluster assembly
GO:0031163	3.85E-05	9.39E-04	metallo-sulfur cluster assembly
GO:0016043	4.23E-05	1.02E-03	cellular component organization
GO:0016051	4.62E-05	1.11E-03	carbohydrate biosynthetic process
GO:0007169	5.38E-05	1.28E-03	transmembrane receptor protein tyrosine kinase signaling pathway
GO:0007167	5.38E-05	1.27E-03	enzyme linked receptor protein signaling pathway
GO:0035303	5.51E-05	1.29E-03	regulation of dephosphorylation
GO:0035304	5.51E-05	1.28E-03	regulation of protein dephosphorylation
GO:0055114	5.71E-05	1.32E-03	oxidation-reduction process
GO:0009617	6.63E-05	1.52E-03	response to bacterium
GO:0071840	7.19E-05	1.64E-03	cellular component organization or biogenesis
GO:1901576	7.93E-05	1.79E-03	organic substance biosynthetic process
GO:0009891	8.01E-05	1.80E-03	positive regulation of biosynthetic process
GO:0000272	9.51E-05	2.12E-03	polysaccharide catabolic process
GO:0072593	1.08E-04	2.40E-03	reactive oxygen species metabolic process
GO:0031328	1.08E-04	2.38E-03	positive regulation of cellular biosynthetic process
GO:1901565	1.10E-04	2.40E-03	organonitrogen compound catabolic process
GO:0042335	1.28E-04	2.76E-03	cuticle development
GO:1902680	1.37E-04	2.95E-03	positive regulation of RNA biosynthetic process
GO:0045893	1.37E-04	2.93E-03	positive regulation of transcription, DNA-templated
GO:1903508	1.37E-04	2.91E-03	positive regulation of nucleic acid-templated transcription
GO:0042743	1.43E-04	3.01E-03	hydrogen peroxide metabolic process

	GO:0032535	1.62E-04	3.38E-03	regulation of cellular component size
	GO:0090066	1.62E-04	3.35E-03	regulation of anatomical structure size
	GO:0051254	1.89E-04	3.90E-03	positive regulation of RNA metabolic process
	GO:0045935	1.92E-04	3.93E-03	positive regulation of nucleobase-containing compound metabolic process
	GO:0050790	1.94E-04	3.94E-03	regulation of catalytic activity
	GO:0044550	1.99E-04	4.02E-03	secondary metabolite biosynthetic process
	GO:0045229	2.01E-04	4.03E-03	external encapsulating structure organization
	GO:0051640	2.03E-04	4.04E-03	organelle localization
	GO:0009411	2.18E-04	4.32E-03	response to UV
	GO:0065009	2.31E-04	4.56E-03	regulation of molecular function
	GO:0071704	2.56E-04	5.00E-03	organic substance metabolic process
	GO:0016143	2.73E-04	5.30E-03	S-glycoside metabolic process
	GO:0019760	2.73E-04	5.27E-03	glucosinolate metabolic process
	GO:0019757	2.73E-04	5.24E-03	glycosinolate metabolic process
	GO:0009832	2.93E-04	5.60E-03	plant-type cell wall biogenesis
	GO:0042546	3.10E-04	5.88E-03	cell wall biogenesis
	GO:0009058	3.12E-04	5.87E-03	biosynthetic process
	GO:0040008	3.36E-04	6.30E-03	regulation of growth
	GO:0010015	3.46E-04	6.45E-03	root morphogenesis
	GO:0010557	3.47E-04	6.43E-03	positive regulation of macromolecule biosynthetic process
	GO:0009767	3.86E-04	7.10E-03	photosynthetic electron transport chain
	GO:0090407	3.88E-04	7.09E-03	organophosphate biosynthetic process
	GO:0051173	4.57E-04	8.30E-03	positive regulation of nitrogen compound metabolic process
	GO:0007166	4.57E-04	8.26E-03	cell surface receptor signaling pathway
	GO:0010628	4.61E-04	8.27E-03	positive regulation of gene expression
	GO:0016144	5.03E-04	8.98E-03	S-glycoside biosynthetic process
	GO:0019758	5.03E-04	8.93E-03	glycosinolate biosynthetic process
	GO:0019761	5.03E-04	8.88E-03	glucosinolate biosynthetic process
Cot	GO:0007169	6.83E-08	2.10E-04	transmembrane receptor protein tyrosine kinase signaling pathway
	GO:0007167	6.83E-08	1.05E-04	enzyme linked receptor protein signaling pathway
	GO:0016556	5.19E-07	5.32E-04	mRNA modification
	GO:0007166	7.33E-07	5.63E-04	cell surface receptor signaling pathway
	GO:0005984	9.28E-07	5.70E-04	disaccharide metabolic process
	GO:0019682	1.12E-06	5.71E-04	glyceraldehyde-3-phosphate metabolic process
	GO:0009653	1.26E-06	5.52E-04	anatomical structure morphogenesis
	GO:0009069	8.44E-06	3.24E-03	serine family amino acid metabolic process
	GO:0009311	1.10E-05	3.75E-03	oligosaccharide metabolic process
	GO:0006081	1.11E-05	3.41E-03	cellular aldehyde metabolic process
	GO:0044550	1.63E-05	4.56E-03	secondary metabolite biosynthetic process
	GO:0019288	2.10E-05	5.39E-03	isopentenyl diphosphate biosynthetic process, methylerythritol 4-phosphate pathway
	GO:0009240	2.10E-05	4.97E-03	isopentenyl diphosphate biosynthetic process
	GO:0046490	2.10E-05	4.62E-03	isopentenyl diphosphate metabolic process
	GO:0006564	2.53E-05	5.17E-03	L-serine biosynthetic process
	GO:0006563	2.53E-05	4.85E-03	L-serine metabolic process
	GO:0044712	3.95E-05	7.14E-03	single-organism catabolic process
	GO:0042744	3.98E-05	6.79E-03	hydrogen peroxide catabolic process
	GO:0009070	3.98E-05	6.43E-03	serine family amino acid biosynthetic process

	GO:0019748	6.50E-05	9.98E-03	secondary metabolic process
1L	none	---	---	---
oL	GO:0071554	2.34E-22	7.19E-19	cell wall organization or biogenesis
	GO:0071555	6.51E-19	1.00E-15	cell wall organization
	GO:0045229	8.00E-18	8.19E-15	external encapsulating structure organization
	GO:0019682	1.67E-14	1.29E-11	glyceraldehyde-3-phosphate metabolic process
	GO:0019288	1.73E-14	1.06E-11	isopentenyl diphosphate biosynthetic process, methylerythritol 4-phosphate pathway
	GO:0009240	1.73E-14	8.86E-12	isopentenyl diphosphate biosynthetic process
	GO:0046490	1.73E-14	7.59E-12	isopentenyl diphosphate metabolic process
	GO:0007169	1.96E-14	7.54E-12	transmembrane receptor protein tyrosine kinase signaling pathway
	GO:0007167	1.96E-14	6.70E-12	enzyme linked receptor protein signaling pathway
	GO:0006629	8.39E-13	2.58E-10	lipid metabolic process
	GO:0007166	2.71E-12	7.58E-10	cell surface receptor signaling pathway
	GO:0071840	3.56E-11	9.10E-09	cellular component organization or biogenesis
	GO:0006081	6.90E-11	1.63E-08	cellular aldehyde metabolic process
	GO:0005976	1.91E-10	4.18E-08	polysaccharide metabolic process
	GO:0016043	7.07E-10	1.45E-07	cellular component organization
	GO:0005975	8.83E-10	1.70E-07	carbohydrate metabolic process
	GO:0000272	3.33E-09	6.01E-07	polysaccharide catabolic process
	GO:0010075	3.82E-09	6.51E-07	regulation of meristem growth
	GO:0009832	8.92E-09	1.44E-06	plant-type cell wall biogenesis
	GO:0008152	1.09E-08	1.68E-06	metabolic process
	GO:0006090	1.11E-08	1.63E-06	pyruvate metabolic process
	GO:0042546	2.26E-08	3.15E-06	cell wall biogenesis
	GO:0048509	3.21E-08	4.29E-06	regulation of meristem development
	GO:0009653	3.39E-08	4.33E-06	anatomical structure morphogenesis
	GO:0010393	3.61E-08	4.44E-06	galacturonan metabolic process
	GO:0045488	3.61E-08	4.27E-06	pectin metabolic process
	GO:0006468	5.86E-08	6.67E-06	protein phosphorylation
	GO:0045490	7.68E-08	8.43E-06	pectin catabolic process
	GO:0007165	1.34E-07	1.42E-05	signal transduction
	GO:0010027	1.43E-07	1.46E-05	thylakoid membrane organization
	GO:0009668	1.43E-07	1.41E-05	plastid membrane organization
	GO:0006793	1.67E-07	1.60E-05	phosphorus metabolic process
	GO:0040008	2.49E-07	2.32E-05	regulation of growth
	GO:0071669	3.19E-07	2.88E-05	plant-type cell wall organization or biogenesis
	GO:0008654	3.53E-07	3.10E-05	phospholipid biosynthetic process
	GO:0071704	3.91E-07	3.33E-05	organic substance metabolic process
	GO:0016042	5.16E-07	4.28E-05	lipid catabolic process
	GO:0048638	8.59E-07	6.94E-05	regulation of developmental growth
	GO:0006644	1.04E-06	8.19E-05	phospholipid metabolic process
	GO:0010103	1.06E-06	8.10E-05	stomatal complex morphogenesis
	GO:0090626	1.06E-06	7.91E-05	plant epidermis morphogenesis
	GO:0010207	1.18E-06	8.66E-05	photosystem II assembly
	GO:0044238	1.36E-06	9.68E-05	primary metabolic process
	GO:0006796	1.37E-06	9.58E-05	phosphate-containing compound metabolic process
	GO:0008610	1.84E-06	1.25E-04	lipid biosynthetic process
	GO:0008361	1.88E-06	1.26E-04	regulation of cell size

		GO:0009926	3.39E-06	2.22E-04	auxin polar transport
		GO:0010015	4.72E-06	3.02E-04	root morphogenesis
		GO:0044711	4.76E-06	2.98E-04	single-organism biosynthetic process
		GO:0009914	6.37E-06	3.91E-04	hormone transport
		GO:0060918	6.37E-06	3.84E-04	auxin transport
		GO:0044699	7.03E-06	4.15E-04	single-organism process
		GO:2000026	7.41E-06	4.29E-04	regulation of multicellular organismal development
		GO:0019684	7.73E-06	4.39E-04	photosynthesis, light reaction
		GO:0044802	9.17E-06	5.12E-04	single-organism membrane organization
		GO:0009056	1.10E-05	6.04E-04	catabolic process
		GO:0044710	1.13E-05	6.07E-04	single-organism metabolic process
		GO:0051239	1.23E-05	6.51E-04	regulation of multicellular organismal process
		GO:0009886	1.50E-05	7.81E-04	post-embryonic morphogenesis
		GO:0044767	2.06E-05	1.05E-03	single-organism developmental process
		GO:0007017	2.57E-05	1.29E-03	microtubule-based process
		GO:1901135	3.69E-05	1.83E-03	carbohydrate derivative metabolic process
		GO:0046148	5.02E-05	2.45E-03	pigment biosynthetic process
		GO:0048453	5.15E-05	2.47E-03	sepal formation
		GO:0048451	5.15E-05	2.43E-03	petal formation
		GO:0044255	5.59E-05	2.60E-03	cellular lipid metabolic process
		GO:0050793	5.69E-05	2.61E-03	regulation of developmental process
		GO:0044712	5.82E-05	2.63E-03	single-organism catabolic process
		GO:0009825	5.85E-05	2.61E-03	multidimensional cell growth
		GO:0043436	5.96E-05	2.62E-03	oxoacid metabolic process
		GO:0006082	5.96E-05	2.58E-03	organic acid metabolic process
		GO:0032502	6.37E-05	2.72E-03	developmental process
		GO:0016310	6.65E-05	2.80E-03	phosphorylation
		GO:0051186	8.06E-05	3.35E-03	cofactor metabolic process
		GO:0009664	8.32E-05	3.41E-03	plant-type cell wall organization
		GO:0009834	1.20E-04	4.83E-03	plant-type secondary cell wall biogenesis
		GO:0008283	1.21E-04	4.81E-03	cell proliferation
		GO:0048767	1.21E-04	4.75E-03	root hair elongation
		GO:0015995	1.29E-04	5.00E-03	chlorophyll biosynthetic process
		GO:0006790	1.36E-04	5.22E-03	sulfur compound metabolic process
		GO:0061024	1.59E-04	6.01E-03	membrane organization
		GO:0048449	1.90E-04	7.13E-03	floral organ formation
		GO:0000226	1.95E-04	7.23E-03	microtubule cytoskeleton organization
		GO:0000911	1.96E-04	7.17E-03	cytokinesis by cell plate formation
		GO:0006098	2.12E-04	7.66E-03	pentose-phosphate shunt
		GO:0051156	2.12E-04	7.57E-03	glucose 6-phosphate metabolic process
		GO:0000271	2.15E-04	7.59E-03	polysaccharide biosynthetic process
		GO:0040007	2.30E-04	8.04E-03	growth
		GO:0009069	2.41E-04	8.31E-03	serine family amino acid metabolic process
		GO:0006739	2.61E-04	8.91E-03	NADP metabolic process
oL	yS	GO:0019682	1.72E-17	5.27E-14	glyceraldehyde-3-phosphate metabolic process
		GO:0006081	9.91E-15	1.52E-11	cellular aldehyde metabolic process
		GO:0044711	7.03E-13	7.20E-10	single-organism biosynthetic process
		GO:0006629	1.83E-12	1.41E-09	lipid metabolic process
		GO:0000023	3.30E-12	2.03E-09	maltose metabolic process
		GO:0051186	5.70E-12	2.92E-09	cofactor metabolic process

GO:0005984	8.84E-12	3.88E-09	disaccharide metabolic process
GO:0005982	1.07E-11	4.12E-09	starch metabolic process
GO:0042440	1.14E-11	3.90E-09	pigment metabolic process
GO:0019288	1.18E-11	3.61E-09	isopentenyl diphosphate biosynthetic process, methylerythritol 4-phosphate pathway
GO:0009240	1.18E-11	3.28E-09	isopentenyl diphosphate biosynthetic process
GO:0046490	1.18E-11	3.01E-09	isopentenyl diphosphate metabolic process
GO:0043436	1.50E-11	3.55E-09	oxoacid metabolic process
GO:0006082	1.50E-11	3.30E-09	organic acid metabolic process
GO:0016556	1.55E-11	3.17E-09	mRNA modification
GO:0044710	1.90E-11	3.65E-09	single-organism metabolic process
GO:0008610	2.01E-11	3.63E-09	lipid biosynthetic process
GO:0009657	2.40E-11	4.09E-09	plastid organization
GO:0044699	3.06E-11	4.94E-09	single-organism process
GO:0019252	4.39E-11	6.75E-09	starch biosynthetic process
GO:0019752	1.24E-10	1.81E-08	carboxylic acid metabolic process
GO:0019684	1.29E-10	1.79E-08	photosynthesis, light reaction
GO:0010027	1.79E-10	2.39E-08	thylakoid membrane organization
GO:0009668	1.79E-10	2.29E-08	plastid membrane organization
GO:0010207	3.42E-10	4.20E-08	photosystem II assembly
GO:0006090	3.51E-10	4.15E-08	pyruvate metabolic process
GO:0044802	3.58E-10	4.08E-08	single-organism membrane organization
GO:0016109	4.57E-10	5.02E-08	tetraterpenoid biosynthetic process
GO:0016108	4.57E-10	4.84E-08	tetraterpenoid metabolic process
GO:0016116	4.57E-10	4.68E-08	carotenoid metabolic process
GO:0016117	4.57E-10	4.53E-08	carotenoid biosynthetic process
GO:0009311	5.34E-10	5.13E-08	oligosaccharide metabolic process
GO:0044763	1.20E-09	1.12E-07	single-organism cellular process
GO:0046148	1.34E-09	1.21E-07	pigment biosynthetic process
GO:0044283	1.43E-09	1.25E-07	small molecule biosynthetic process
GO:0009658	2.23E-09	1.90E-07	chloroplast organization
GO:0006733	2.27E-09	1.89E-07	oxidoreduction coenzyme metabolic process
GO:0044255	2.58E-09	2.08E-07	cellular lipid metabolic process
GO:0006811	3.85E-09	3.03E-07	ion transport
GO:0006732	4.35E-09	3.34E-07	coenzyme metabolic process
GO:1901135	4.70E-09	3.52E-07	carbohydrate derivative metabolic process
GO:0044281	5.48E-09	4.01E-07	small molecule metabolic process
GO:0006098	1.20E-08	8.54E-07	pentose-phosphate shunt
GO:0051156	1.20E-08	8.35E-07	glucose 6-phosphate metabolic process
GO:0006644	1.34E-08	9.16E-07	phospholipid metabolic process
GO:0048878	1.59E-08	1.06E-06	chemical homeostasis
GO:0006739	2.02E-08	1.32E-06	NADP metabolic process
GO:0006720	2.02E-08	1.29E-06	isoprenoid metabolic process
GO:0015979	2.70E-08	1.69E-06	photosynthesis
GO:0008299	2.93E-08	1.80E-06	isoprenoid biosynthetic process
GO:0050801	3.36E-08	2.02E-06	ion homeostasis
GO:0016053	7.09E-08	4.19E-06	organic acid biosynthetic process
GO:0046394	7.09E-08	4.11E-06	carboxylic acid biosynthetic process
GO:0009250	9.07E-08	5.16E-06	glucan biosynthetic process
GO:0006520	1.02E-07	5.68E-06	cellular amino acid metabolic process

GO:0055080	1.03E-07	5.63E-06	cation homeostasis
GO:0008652	1.07E-07	5.79E-06	cellular amino acid biosynthetic process
GO:0009070	1.27E-07	6.71E-06	serine family amino acid biosynthetic process
GO:0006793	1.62E-07	8.42E-06	phosphorus metabolic process
GO:0006073	1.63E-07	8.32E-06	cellular glucan metabolic process
GO:0044042	1.63E-07	8.19E-06	glucan metabolic process
GO:0072524	1.73E-07	8.58E-06	pyridine-containing compound metabolic process
GO:0032787	2.31E-07	1.13E-05	monocarboxylic acid metabolic process
GO:0019362	2.48E-07	1.19E-05	pyridine nucleotide metabolic process
GO:0046496	2.48E-07	1.17E-05	nicotinamide nucleotide metabolic process
GO:1901605	2.54E-07	1.18E-05	alpha-amino acid metabolic process
GO:0006796	2.67E-07	1.22E-05	phosphate-containing compound metabolic process
GO:0009069	3.14E-07	1.42E-05	serine family amino acid metabolic process
GO:0006721	3.57E-07	1.59E-05	terpenoid metabolic process
GO:0009653	4.34E-07	1.90E-05	anatomical structure morphogenesis
GO:0009893	4.74E-07	2.05E-05	positive regulation of metabolic process
GO:0016114	5.18E-07	2.21E-05	terpenoid biosynthetic process
GO:0008654	5.96E-07	2.51E-05	phospholipid biosynthetic process
GO:0061024	5.97E-07	2.48E-05	membrane organization
GO:0006873	7.54E-07	3.09E-05	cellular ion homeostasis
GO:0030003	7.54E-07	3.05E-05	cellular cation homeostasis
GO:0006790	9.86E-07	3.93E-05	sulfur compound metabolic process
GO:0006091	1.05E-06	4.12E-05	generation of precursor metabolites and energy
GO:0019344	1.18E-06	4.57E-05	cysteine biosynthetic process
GO:1901565	1.20E-06	4.60E-05	organonitrogen compound catabolic process
GO:0055082	1.23E-06	4.65E-05	cellular chemical homeostasis
GO:0042742	1.97E-06	7.39E-05	defense response to bacterium
GO:0006534	3.37E-06	1.25E-04	cysteine metabolic process
GO:1901607	3.80E-06	1.39E-04	alpha-amino acid biosynthetic process
GO:0044272	3.80E-06	1.37E-04	sulfur compound biosynthetic process
GO:0009902	3.81E-06	1.36E-04	chloroplast relocation
GO:0051644	3.81E-06	1.35E-04	plastid localization
GO:0051667	3.81E-06	1.33E-04	establishment of plastid localization
GO:0019750	3.81E-06	1.32E-04	chloroplast localization
GO:0015994	5.52E-06	1.88E-04	chlorophyll metabolic process
GO:0065008	5.68E-06	1.92E-04	regulation of biological quality
GO:1901617	8.77E-06	2.93E-04	organic hydroxy compound biosynthetic process
GO:0043085	9.53E-06	3.15E-04	positive regulation of catalytic activity
GO:0009617	9.53E-06	3.11E-04	response to bacterium
GO:0009965	1.16E-05	3.74E-04	leaf morphogenesis
GO:0009637	1.44E-05	4.62E-04	response to blue light
GO:0044093	1.47E-05	4.66E-04	positive regulation of molecular function
GO:0019637	1.50E-05	4.70E-04	organophosphate metabolic process
GO:0006820	1.55E-05	4.82E-04	anion transport
GO:0009812	2.29E-05	7.04E-04	flavonoid metabolic process
GO:0006778	2.32E-05	7.06E-04	porphyrin-containing compound metabolic process
GO:0033013	2.32E-05	7.00E-04	tetrapyrrole metabolic process
GO:1901615	3.29E-05	9.81E-04	organic hydroxy compound metabolic process
GO:0042592	3.49E-05	1.03E-03	homeostatic process
GO:0008152	4.34E-05	1.27E-03	metabolic process

	GO:0010103	4.58E-05	1.33E-03	stomatal complex morphogenesis
	GO:0090626	4.58E-05	1.31E-03	plant epidermis morphogenesis
	GO:0015718	4.95E-05	1.41E-03	monocarboxylic acid transport
	GO:0015711	5.36E-05	1.51E-03	organic anion transport
	GO:0009411	5.67E-05	1.58E-03	response to UV
	GO:0051656	5.67E-05	1.57E-03	establishment of organelle localization
	GO:0009813	6.33E-05	1.74E-03	flavonoid biosynthetic process
	GO:0046942	6.52E-05	1.77E-03	carboxylic acid transport
	GO:0019725	6.52E-05	1.76E-03	cellular homeostasis
	GO:0016143	6.81E-05	1.82E-03	S-glycoside metabolic process
	GO:0019760	6.81E-05	1.80E-03	glucosinolate metabolic process
	GO:0019757	6.81E-05	1.79E-03	glycosinolate metabolic process
	GO:0000271	7.26E-05	1.89E-03	polysaccharide biosynthetic process
	GO:0034637	9.71E-05	2.50E-03	cellular carbohydrate biosynthetic process
	GO:0015849	1.13E-04	2.90E-03	organic acid transport
	GO:0000097	1.13E-04	2.87E-03	sulfur amino acid biosynthetic process
	GO:0044264	1.15E-04	2.90E-03	cellular polysaccharide metabolic process
	GO:0009886	1.34E-04	3.35E-03	post-embryonic morphogenesis
	GO:0000096	1.37E-04	3.39E-03	sulfur amino acid metabolic process
	GO:0009072	1.43E-04	3.53E-03	aromatic amino acid family metabolic process
	GO:0016144	1.45E-04	3.52E-03	S-glycoside biosynthetic process
	GO:0019758	1.45E-04	3.50E-03	glycosinolate biosynthetic process
	GO:0019761	1.45E-04	3.47E-03	glucosinolate biosynthetic process
	GO:0044262	1.46E-04	3.47E-03	cellular carbohydrate metabolic process
	GO:0033692	1.57E-04	3.72E-03	cellular polysaccharide biosynthetic process
	GO:0005976	1.85E-04	4.33E-03	polysaccharide metabolic process
	GO:0007169	2.07E-04	4.81E-03	transmembrane receptor protein tyrosine kinase signaling pathway
	GO:0007167	2.07E-04	4.78E-03	enzyme linked receptor protein signaling pathway
	GO:0098771	2.19E-04	5.02E-03	inorganic ion homeostasis
	GO:0048518	2.37E-04	5.39E-03	positive regulation of biological process
	GO:0006812	2.91E-04	6.58E-03	cation transport
	GO:0006952	2.91E-04	6.53E-03	defense response
	GO:0044550	3.07E-04	6.84E-03	secondary metabolite biosynthetic process
	GO:0051188	3.14E-04	6.95E-03	cofactor biosynthetic process
	GO:1901564	3.53E-04	7.74E-03	organonitrogen compound metabolic process
	GO:0051234	3.68E-04	8.02E-03	establishment of localization
	GO:0045088	3.75E-04	8.10E-03	regulation of innate immune response
	GO:0070887	4.01E-04	8.60E-03	cellular response to chemical stimulus
	GO:0016310	4.19E-04	8.93E-03	phosphorylation
	GO:0009743	4.44E-04	9.41E-03	response to carbohydrate
	GO:0035303	4.48E-04	9.43E-03	regulation of dephosphorylation
	GO:0035304	4.48E-04	9.36E-03	regulation of protein dephosphorylation
	GO:0016226	4.51E-04	9.36E-03	iron-sulfur cluster assembly
	GO:0031163	4.51E-04	9.30E-03	metallo-sulfur cluster assembly
	GO:0031347	4.60E-04	9.42E-03	regulation of defense response
	GO:0007166	4.74E-04	9.65E-03	cell surface receptor signaling pathway
Cot	GO:0031347	6.20E-10	1.90E-06	regulation of defense response
	GO:0080134	1.11E-08	1.70E-05	regulation of response to stress
	GO:0048583	4.33E-08	4.43E-05	regulation of response to stimulus

	GO:0048585	6.74E-08	5.18E-05	negative regulation of response to stimulus
	GO:0006811	5.53E-07	3.40E-04	ion transport
	GO:0031348	5.86E-07	3.00E-04	negative regulation of defense response
	GO:0045088	6.84E-07	3.00E-04	regulation of innate immune response
	GO:0009863	6.84E-07	2.62E-04	salicylic acid mediated signaling pathway
	GO:0002682	8.58E-07	2.93E-04	regulation of immune system process
	GO:0050776	8.58E-07	2.63E-04	regulation of immune response
	GO:0019748	1.36E-06	3.79E-04	secondary metabolic process
	GO:0043207	2.14E-06	5.46E-04	response to external biotic stimulus
	GO:0009607	2.78E-06	6.56E-04	response to biotic stimulus
	GO:0051707	5.61E-06	1.23E-03	response to other organism
	GO:0009605	6.88E-06	1.41E-03	response to external stimulus
	GO:0010363	8.19E-06	1.57E-03	regulation of plant-type hypersensitive response
	GO:0050896	8.48E-06	1.53E-03	response to stimulus
	GO:0051704	1.06E-05	1.81E-03	multi-organism process
	GO:0010033	1.15E-05	1.86E-03	response to organic substance
	GO:0006564	1.25E-05	1.92E-03	L-serine biosynthetic process
	GO:0006563	1.25E-05	1.83E-03	L-serine metabolic process
	GO:0010941	2.62E-05	3.66E-03	regulation of cell death
	GO:0043067	2.62E-05	3.50E-03	regulation of programmed cell death
	GO:0080135	3.14E-05	4.02E-03	regulation of cellular response to stress
	GO:0006820	3.65E-05	4.48E-03	anion transport
	GO:0009627	4.01E-05	4.74E-03	systemic acquired resistance
	GO:0002376	4.88E-05	5.55E-03	immune system process
	GO:0044765	5.22E-05	5.72E-03	single-organism transport
	GO:0006040	5.23E-05	5.54E-03	amino sugar metabolic process
	GO:0042221	6.96E-05	7.12E-03	response to chemical
	GO:0046942	7.09E-05	7.03E-03	carboxylic acid transport
	GO:1902578	7.71E-05	7.40E-03	single-organism localization
	GO:0071705	9.29E-05	8.64E-03	nitrogen compound transport
	GO:0015849	9.33E-05	8.42E-03	organic acid transport
	GO:0006612	1.13E-04	9.89E-03	protein targeting to membrane
	GO:0072657	1.13E-04	9.62E-03	protein localization to membrane
	GO:0009814	1.16E-04	9.61E-03	defense response, incompatible interaction
1L	GO:0031347	3.75E-10	1.15E-06	regulation of defense response
	GO:0080134	1.60E-09	2.45E-06	regulation of response to stress
	GO:0048583	9.66E-09	9.89E-06	regulation of response to stimulus
	GO:0045088	2.64E-07	2.03E-04	regulation of innate immune response
	GO:0002682	3.37E-07	2.07E-04	regulation of immune system process
	GO:0050776	3.37E-07	1.72E-04	regulation of immune response
	GO:0010941	4.28E-07	1.88E-04	regulation of cell death
	GO:0043067	4.28E-07	1.64E-04	regulation of programmed cell death
	GO:0006811	4.58E-07	1.56E-04	ion transport
	GO:0010363	5.61E-07	1.72E-04	regulation of plant-type hypersensitive response
	GO:0080135	2.79E-06	7.79E-04	regulation of cellular response to stress
	GO:0050896	3.95E-06	1.01E-03	response to stimulus
	GO:1901698	8.34E-06	1.97E-03	response to nitrogen compound
	GO:0042537	1.02E-05	2.25E-03	benzene-containing compound metabolic process
	GO:0006612	1.50E-05	3.07E-03	protein targeting to membrane
	GO:0072657	1.50E-05	2.88E-03	protein localization to membrane

	GO:0043207	1.95E-05	3.52E-03	response to external biotic stimulus
	GO:0009607	2.47E-05	4.22E-03	response to biotic stimulus
	GO:0045087	2.53E-05	4.10E-03	innate immune response
	GO:1901700	2.63E-05	4.03E-03	response to oxygen-containing compound
	GO:0044765	2.64E-05	3.86E-03	single-organism transport
	GO:0090150	2.84E-05	3.97E-03	establishment of protein localization to membrane
	GO:0006955	3.29E-05	4.40E-03	immune response
	GO:0042221	3.30E-05	4.22E-03	response to chemical
	GO:1902578	4.01E-05	4.93E-03	single-organism localization
	GO:0010035	4.64E-05	5.48E-03	response to inorganic substance
	GO:0009814	5.48E-05	6.23E-03	defense response, incompatible interaction
	GO:0009605	6.12E-05	6.71E-03	response to external stimulus
	GO:0048585	8.20E-05	8.68E-03	negative regulation of response to stimulus
2L	GO:1901698	8.71E-10	2.67E-06	response to nitrogen compound
	GO:0031347	8.41E-08	1.29E-04	regulation of defense response
	GO:0080134	2.55E-07	2.61E-04	regulation of response to stress
	GO:0043207	3.58E-07	2.75E-04	response to external biotic stimulus
	GO:0009607	4.83E-07	2.96E-04	response to biotic stimulus
	GO:0042537	5.05E-07	2.59E-04	benzene-containing compound metabolic process
	GO:0006811	1.09E-06	4.80E-04	ion transport
	GO:0071705	1.41E-06	5.41E-04	nitrogen compound transport
	GO:0015706	1.42E-06	4.85E-04	nitrate transport
	GO:0015698	1.65E-06	5.07E-04	inorganic anion transport
	GO:0051707	1.77E-06	4.95E-04	response to other organism
	GO:0009863	2.14E-06	5.48E-04	salicylic acid mediated signaling pathway
	GO:0051704	3.58E-06	8.45E-04	multi-organism process
	GO:0050896	4.05E-06	8.88E-04	response to stimulus
	GO:0001101	4.89E-06	1.00E-03	response to acid chemical
	GO:0044765	5.00E-06	9.60E-04	single-organism transport
	GO:1901700	5.16E-06	9.32E-04	response to oxygen-containing compound
	GO:0010167	5.73E-06	9.77E-04	response to nitrate
	GO:0009605	5.97E-06	9.65E-04	response to external stimulus
	GO:1902578	7.95E-06	1.22E-03	single-organism localization
	GO:0048583	9.71E-06	1.42E-03	regulation of response to stimulus
	GO:0045088	1.05E-05	1.46E-03	regulation of innate immune response
	GO:0045087	1.15E-05	1.54E-03	innate immune response
	GO:0018958	1.20E-05	1.54E-03	phenol-containing compound metabolic process
	GO:0002682	1.28E-05	1.58E-03	regulation of immune system process
	GO:0050776	1.28E-05	1.52E-03	regulation of immune response
	GO:0006955	1.52E-05	1.73E-03	immune response
	GO:0006820	1.84E-05	2.02E-03	anion transport
	GO:0002376	2.08E-05	2.20E-03	immune system process
	GO:0042221	2.48E-05	2.53E-03	response to chemical
	GO:0006952	3.22E-05	3.19E-03	defense response
	GO:0046189	4.17E-05	4.00E-03	phenol-containing compound biosynthetic process
	GO:0009697	4.17E-05	3.88E-03	salicylic acid biosynthetic process
	GO:0009696	4.17E-05	3.76E-03	salicylic acid metabolic process
	GO:0098542	5.78E-05	5.07E-03	defense response to other organism
	GO:0010941	6.77E-05	5.77E-03	regulation of cell death
	GO:0043067	6.77E-05	5.62E-03	regulation of programmed cell death

		GO:0009814	7.70E-05	6.22E-03	defense response, incompatible interaction
		GO:1901699	8.38E-05	6.60E-03	cellular response to nitrogen compound
		GO:0010035	8.41E-05	6.46E-03	response to inorganic substance
		GO:0006810	9.16E-05	6.86E-03	transport
		GO:0010363	9.74E-05	7.12E-03	regulation of plant-type hypersensitive response
		GO:0009627	9.74E-05	6.96E-03	systemic acquired resistance
		GO:0007165	1.11E-04	7.75E-03	signal transduction
		GO:0071495	1.15E-04	7.87E-03	cellular response to endogenous stimulus
		GO:0009723	1.41E-04	9.39E-03	response to ethylene
		GO:0010200	1.48E-04	9.65E-03	response to chitin
		GO:0010243	1.48E-04	9.45E-03	response to organonitrogen compound
oL	1L+2L	GO:0031347	6.78E-09	2.08E-05	regulation of defense response
		GO:0080134	1.50E-08	2.31E-05	regulation of response to stress
		GO:0006811	1.06E-07	1.08E-04	ion transport
		GO:1901698	2.86E-07	2.20E-04	response to nitrogen compound
		GO:0042537	3.33E-07	2.05E-04	benzene-containing compound metabolic process
		GO:0048583	4.38E-07	2.24E-04	regulation of response to stimulus
		GO:0043207	5.94E-07	2.61E-04	response to external biotic stimulus
		GO:0009607	7.64E-07	2.93E-04	response to biotic stimulus
		GO:0045088	1.21E-06	4.14E-04	regulation of innate immune response
		GO:0050896	1.37E-06	4.19E-04	response to stimulus
		GO:0002682	1.48E-06	4.13E-04	regulation of immune system process
		GO:0050776	1.48E-06	3.79E-04	regulation of immune response
		GO:0042221	1.63E-06	3.85E-04	response to chemical
		GO:0010941	1.80E-06	3.95E-04	regulation of cell death
		GO:0043067	1.80E-06	3.68E-04	regulation of programmed cell death
		GO:0010363	3.28E-06	6.29E-04	regulation of plant-type hypersensitive response
		GO:0009605	3.59E-06	6.48E-04	response to external stimulus
		GO:0044765	4.98E-06	8.50E-04	single-organism transport
		GO:0045087	5.75E-06	9.29E-04	innate immune response
		GO:0001101	6.94E-06	1.06E-03	response to acid chemical
		GO:0006955	7.39E-06	1.08E-03	immune response
		GO:1902578	7.40E-06	1.03E-03	single-organism localization
		GO:1901700	9.77E-06	1.30E-03	response to oxygen-containing compound
		GO:0051707	1.05E-05	1.34E-03	response to other organism
		GO:0071705	1.15E-05	1.42E-03	nitrogen compound transport
		GO:0080135	1.19E-05	1.41E-03	regulation of cellular response to stress
		GO:0002376	1.59E-05	1.81E-03	immune system process
		GO:0010035	1.67E-05	1.83E-03	response to inorganic substance
		GO:0051704	1.82E-05	1.93E-03	multi-organism process
		GO:0006820	2.79E-05	2.86E-03	anion transport
		GO:0006952	2.99E-05	2.96E-03	defense response
		GO:0009863	3.70E-05	3.55E-03	salicylic acid mediated signaling pathway
		GO:0009814	4.21E-05	3.92E-03	defense response, incompatible interaction
		GO:0006810	6.10E-05	5.51E-03	transport
		GO:0009415	6.99E-05	6.13E-03	response to water
		GO:0015698	7.78E-05	6.64E-03	inorganic anion transport
		GO:0098542	9.47E-05	7.86E-03	defense response to other organism
		GO:0009627	9.66E-05	7.80E-03	systemic acquired resistance
		GO:0046189	9.77E-05	7.69E-03	phenol-containing compound biosynthetic process

GO:0009697	9.77E-05	7.50E-03	salicylic acid biosynthetic process
GO:0009696	9.77E-05	7.32E-03	salicylic acid metabolic process
GO:0010033	1.01E-04	7.37E-03	response to organic substance
GO:0009755	1.18E-04	8.46E-03	hormone-mediated signaling pathway
GO:0015706	1.19E-04	8.32E-03	nitrate transport
GO:0006612	1.41E-04	9.65E-03	protein targeting to membrane
GO:0072657	1.41E-04	9.44E-03	protein localization to membrane
GO:0018958	1.45E-04	9.45E-03	phenol-containing compound metabolic process
GO:0051234	1.45E-04	9.29E-03	establishment of localization
