# Supplementary material for: C3 cotyledons are followed by C4 leaves: intra-individual transcriptome analysis of Salsola soda (Chenopodiaceae)
Source: J Exp Bot. 2016 Sep 22;68(2):161–76. doi: 10.1093/jxb/erw343 (PMC5853821; doi:10.1093/jxb/erw343)
Supplement: Supplementary_Table_S6 [file erw343_suppl_supplementary_table_s6.pdf]

Supplementary Table S6.

Cluster 8:				
**GO Term**	**Description**	**P-value**	**FDR**	**Enrichment**
GO:0000461	endonucleolytic cleavage to generate mature 3'-end of SSU-rRNA from (SSU-rRNA, 5.8S rRNA, LSU-rRNA)	8.92E-04	1.76E-01	33.42
GO:0042274	ribosomal small subunit biogenesis	3.84E-06	2.02E-03	26.74
GO:0046685	response to arsenic-containing substance	3.21E-04	8.46E-02	11.14
GO:0022613	ribonucleoprotein complex biogenesis	2.19E-05	9.90E-03	4.27
GO:0042254	ribosome biogenesis	2.34E-04	6.72E-02	3.89
GO:0006412	translation	3.19E-09	1.01E-05	3.73
GO:0043043	peptide biosynthetic process	3.83E-09	6.04E-06	3.7
GO:0001510	RNA methylation	4.84E-04	1.18E-01	3.56
GO:0006518	peptide metabolic process	9.19E-09	9.67E-06	3.55
GO:0043604	amide biosynthetic process	1.40E-08	1.10E-05	3.48
GO:0043603	cellular amide metabolic process	9.05E-08	5.72E-05	3.19
GO:0044085	cellular component biogenesis	1.68E-04	5.32E-02	3.1
GO:1901566	organonitrogen compound biosynthetic process	5.70E-04	1.29E-01	1.87
GO:0034645	cellular macromolecule biosynthetic process	8.28E-04	1.74E-01	1.67
GO:0044267	cellular protein metabolic process	9.05E-05	3.57E-02	1.62
GO:0019538	protein metabolic process	1.48E-04	5.21E-02	1.53
**Cluster 11:**				
**GO Term**	**Description**	**P-value**	**FDR**	**Enrichment**
GO:0071426	ribonucleoprotein complex export from nucleus	4.06E-04	9.87E-02	13.49
GO:0071428	rRNA-containing ribonucleoprotein complex export from nucleus	4.06E-04	9.16E-02	13.49
GO:0033750	ribosome localization	4.06E-04	8.55E-02	13.49
GO:0033753	establishment of ribosome localization	4.06E-04	8.02E-02	13.49
GO:0000054	ribosomal subunit export from nucleus	4.06E-04	7.54E-02	13.49
GO:0000055	ribosomal large subunit export from nucleus	4.06E-04	7.13E-02	13.49
GO:0051181	cofactor transport	8.75E-04	1.45E-01	7.71
GO:0000338	protein deneddylation	5.99E-06	4.73E-03	4.5
GO:0010388	cullin deneddylation	5.99E-06	3.79E-03	4.5
GO:0051168	nuclear export	8.94E-05	4.03E-02	4.5
GO:0009640	photomorphogenesis	7.40E-05	3.90E-02	3.6
GO:0006913	nucleocytoplasmic transport	2.26E-06	7.15E-03	3.33
GO:0051169	nuclear transport	2.26E-06	3.57E-03	3.33
GO:0009791	post-embryonic development	5.38E-06	5.67E-03	2.95
GO:0034504	protein localization to nucleus	3.64E-04	1.28E-01	2.92
GO:0006606	protein import into nucleus	3.64E-04	1.15E-01	2.92
GO:1902593	single-organism nuclear import	3.64E-04	1.05E-01	2.92
GO:0051170	nuclear import	3.64E-04	9.59E-02	2.92
GO:0009408	response to heat	9.37E-04	1.48E-01	2.55
GO:0009266	response to temperature stimulus	2.49E-04	9.85E-02	2.06
