# Supplementary material for: C3 cotyledons are followed by C4 leaves: intra-individual transcriptome analysis of Salsola soda (Chenopodiaceae)
Source: J Exp Bot. 2016 Sep 22;68(2):161–76. doi: 10.1093/jxb/erw343 (PMC5853821; doi:10.1093/jxb/erw343)
Supplement: Supplementary_Table_S7 [file erw343_suppl_supplementary_table_s7.pdf]

Supplementary Table S7.

GO Term	Description	P-value	FDR	Enrichment
GO:0071216	cellular response to biotic stimulus	2.79E-05	6.29E-03	32.87
GO:0071219	cellular response to molecule of bacterial origin	2.79E-05	5.87E-03	32.87
GO:0048574	long-day photoperiodism, flowering	9.23E-04	7.47E-02	32.87
GO:0012501	programmed cell death	2.73E-05	6.64E-03	18.78
GO:0009581	detection of external stimulus	8.91E-04	7.41E-02	14.09
GO:0008219	cell death	6.60E-04	6.32E-02	9.39
GO:0016265	death	6.60E-04	6.13E-02	9.39
GO:0002237	response to molecule of bacterial origin	1.30E-07	4.11E-04	8.65
GO:0031348	negative regulation of defense response	1.15E-05	3.01E-03	5.48
GO:0048585	negative regulation of response to stimulus	7.46E-07	1.18E-03	5.34
GO:0009863	salicylic acid mediated signaling pathway	2.74E-06	1.24E-03	5.19
GO:0009862	systemic acquired resistance, salicylic acid mediated signaling pathway	4.97E-04	5.61E-02	4.9
GO:0009627	systemic acquired resistance	3.56E-05	7.03E-03	4.83
GO:0009697	salicylic acid biosynthetic process	5.67E-04	5.97E-02	4.79
GO:0009696	salicylic acid metabolic process	5.67E-04	5.78E-02	4.79
GO:0046189	phenol-containing compound biosynthetic process	6.45E-04	6.37E-02	4.7
GO:0018958	phenol-containing compound metabolic process	8.25E-04	7.45E-02	4.51
GO:0009814	defense response, incompatible interaction	4.42E-05	7.76E-03	4.3
GO:0031347	regulation of defense response	1.13E-06	8.92E-04	4.21
GO:0045087	innate immune response	1.73E-06	1.09E-03	4.08
GO:0006955	immune response	2.36E-06	1.24E-03	3.98
GO:0042537	benzene-containing compound metabolic process	8.28E-04	7.07E-02	3.98
GO:0002682	regulation of immune system process	2.20E-04	3.02E-02	3.91
GO:0050776	regulation of immune response	2.20E-04	2.90E-02	3.91
GO:0080134	regulation of response to stress	4.67E-06	1.64E-03	3.78
GO:0048583	regulation of response to stimulus	1.12E-06	1.18E-03	3.63
GO:0045088	regulation of innate immune response	8.27E-04	7.25E-02	3.61
GO:0002376	immune system process	3.35E-06	1.32E-03	3.52
GO:1901698	response to nitrogen compound	3.89E-04	4.73E-02	2.86
GO:0006952	defense response	1.02E-05	2.92E-03	2.68
GO:0006812	cation transport	3.85E-05	7.16E-03	2.68
GO:0043207	response to external biotic stimulus	6.71E-05	1.06E-02	2.45
GO:0006811	ion transport	5.88E-06	1.86E-03	2.43
GO:0048519	negative regulation of biological process	2.25E-04	2.84E-02	2.43
GO:0009607	response to biotic stimulus	7.78E-05	1.17E-02	2.42
GO:0009605	response to external stimulus	5.77E-05	9.59E-03	2.19
GO:0007165	signal transduction	1.33E-04	1.91E-02	2.09
GO:0044765	single-organism transport	4.05E-04	4.74E-02	1.63
GO:1902578	single-organism localization	5.09E-04	5.55E-02	1.61
