# Supplementary material for: C3 cotyledons are followed by C4 leaves: intra-individual transcriptome analysis of Salsola soda (Chenopodiaceae)
Source: J Exp Bot. 2016 Sep 22;68(2):161–76. doi: 10.1093/jxb/erw343 (PMC5853821; doi:10.1093/jxb/erw343)
Supplement: Supplementary_Table_S8 [file erw343_suppl_supplementary_table_s8.pdf]

Supplementary Table S8.

Cluster 1:				
**GO Term**	**Description**	**P-value**	**FDR**	**Enrichment**
GO:0006817	phosphate ion transport	5.42E-04	1.07E-01	9.27
GO:0045490	pectin catabolic process	6.67E-07	4.21E-04	5.69
GO:0009853	photorespiration	2.15E-08	6.80E-05	5.38
GO:1902600	hydrogen ion transmembrane transport	3.46E-04	9.12E-02	5.03
GO:0010393	galacturonan metabolic process	5.11E-07	5.38E-04	4.95
GO:0045488	pectin metabolic process	5.11E-07	4.03E-04	4.95
GO:0000272	polysaccharide catabolic process	5.68E-06	2.24E-03	4.72
GO:0080129	proteasome core complex assembly	2.43E-04	6.97E-02	4.63
GO:0043094	cellular metabolic compound salvage	2.55E-07	4.02E-04	4.57
GO:0006818	hydrogen transport	6.59E-04	1.23E-01	4.56
GO:0015992	proton transport	6.59E-04	1.16E-01	4.56
GO:0042546	cell wall biogenesis	3.91E-04	8.83E-02	3.91
GO:0042744	hydrogen peroxide catabolic process	8.36E-04	1.39E-01	3.54
GO:0016052	carbohydrate catabolic process	5.04E-05	1.59E-02	2.87
GO:0071554	cell wall organization or biogenesis	4.58E-04	9.65E-02	2.14
GO:0055114	oxidation-reduction process	1.41E-06	7.43E-04	2.09
GO:0009056	catabolic process	1.94E-06	8.76E-04	1.85
GO:0044712	single-organism catabolic process	3.65E-04	8.86E-02	1.8
GO:1901575	organic substance catabolic process	4.59E-05	1.61E-02	1.79
GO:0044699	single-organism process	9.09E-04	1.44E-01	1.2
**Cluster 18:**				
**GO Term**	**Description**	**P-value**	**FDR**	**Enrichment**
GO:0001944	vasculature development	5.39E-05	2.43E-02	26.4
GO:0045490	pectin catabolic process	4.95E-07	1.56E-03	6.6
GO:0000272	polysaccharide catabolic process	3.62E-06	2.86E-03	5.48
GO:0010393	galacturonan metabolic process	1.64E-06	2.58E-03	5.37
GO:0045488	pectin metabolic process	1.64E-06	1.72E-03	5.37
GO:0042545	cell wall modification	4.28E-06	2.71E-03	4.25
GO:0016052	carbohydrate catabolic process	8.51E-06	4.48E-03	3.43
