# Supplementary material for: C3 cotyledons are followed by C4 leaves: intra-individual transcriptome analysis of Salsola soda (Chenopodiaceae)
Source: J Exp Bot. 2016 Sep 22;68(2):161–76. doi: 10.1093/jxb/erw343 (PMC5853821; doi:10.1093/jxb/erw343)
Supplement: Supplementary_Table_S9 [file erw343_suppl_supplementary_table_s9.pdf]

**Supplementary Table S9.**

| Cluster 3: | | | | |
|---|---|---|---|---|
| **GO Term** | **Description** | **P-value** | **FDR** | **Enrichment** |
| GO:0006564 | L-serine biosynthetic process | 1.19E-06 | 3.74E-03 | 30.17 |
| GO:0006563 | L-serine metabolic process | 1.19E-06 | 1.87E-03 | 30.17 |
| GO:1901565 | organonitrogen compound catabolic process | 5.97E-05 | 6.29E-02 | 3.85 |
| GO:0006040 | amino sugar metabolic process | 6.07E-05 | 4.79E-02 | 10.78 |
| GO:0046916 | cellular transition metal ion homeostasis | 8.35E-05 | 5.28E-02 | 5.49 |
| GO:0006875 | cellular metal ion homeostasis | 1.16E-04 | 6.11E-02 | 5.25 |
| GO:0006032 | chitin catabolic process | 1.41E-04 | 6.35E-02 | 22.63 |
| GO:0006030 | chitin metabolic process | 1.41E-04 | 5.56E-02 | 22.63 |
| GO:0006026 | aminoglycan catabolic process | 1.41E-04 | 4.94E-02 | 22.63 |
| GO:1901072 | glucosamine-containing compound catabolic process | 1.41E-04 | 4.45E-02 | 22.63 |
| GO:0046348 | amino sugar catabolic process | 1.41E-04 | 4.04E-02 | 22.63 |
| GO:0055076 | transition metal ion homeostasis | 2.45E-04 | 6.45E-02 | 4.73 |
| GO:1901071 | glucosamine-containing compound metabolic process | 3.43E-04 | 8.34E-02 | 18.1 |
| GO:0055065 | metal ion homeostasis | 3.67E-04 | 8.29E-02 | 4.47 |
| GO:0098771 | inorganic ion homeostasis | 6.04E-04 | 1.27E-01 | 4.16 |
| GO:1901136 | carbohydrate derivative catabolic process | 6.41E-04 | 1.27E-01 | 6.86 |
| GO:0031347 | regulation of defense response | 8.81E-04 | 1.64E-01 | 2.9 |
| **Cluster 7:** | | | | |
| **GO Term** | **Description** | **P-value** | **FDR** | **Enrichment** |
| GO:0019685 | photosynthesis, dark reaction | 3.13E-05 | 6.73E-04 | 13.34 |
| GO:0019253 | reductive pentose-phosphate cycle | 3.13E-05 | 6.68E-04 | 13.34 |
| GO:0042454 | ribonucleoside catabolic process | 4.19E-04 | 6.94E-03 | 13.34 |
| GO:0048564 | photosystem I assembly | 4.19E-04 | 6.90E-03 | 13.34 |
| GO:0019684 | photosynthesis, light reaction | 1.71E-20 | 4.49E-18 | 11.21 |
| GO:0015979 | photosynthesis | 3.36E-18 | 4.82E-16 | 8.77 |
| GO:0006546 | glycine catabolic process | 1.08E-04 | 1.96E-03 | 8.34 |
| GO:0016109 | tetraterpenoid biosynthetic process | 4.59E-14 | 3.81E-12 | 7.92 |
| GO:0016108 | tetraterpenoid metabolic process | 4.59E-14 | 3.71E-12 | 7.92 |
| GO:0016116 | carotenoid metabolic process | 4.59E-14 | 3.62E-12 | 7.92 |
| GO:0016117 | carotenoid biosynthetic process | 4.59E-14 | 3.53E-12 | 7.92 |
| GO:0009902 | chloroplast relocation | 5.06E-12 | 3.40E-10 | 7.91 |
| GO:0051644 | plastid localization | 5.06E-12 | 3.33E-10 | 7.91 |
| GO:0051667 | establishment of plastid localization | 5.06E-12 | 3.26E-10 | 7.91 |
| GO:0019750 | chloroplast localization | 5.06E-12 | 3.20E-10 | 7.91 |
| GO:0010027 | thylakoid membrane organization | 4.48E-25 | 2.36E-22 | 7.75 |
| GO:0009668 | plastid membrane organization | 4.48E-25 | 2.02E-22 | 7.75 |
| GO:0010207 | photosystem II assembly | 2.27E-18 | 3.41E-16 | 7.71 |
| GO:0006544 | glycine metabolic process | 2.28E-04 | 3.96E-03 | 7.41 |
| GO:0015988 | energy coupled proton transmembrane transport, against electrochemical gradient | 2.28E-04 | 3.93E-03 | 7.41 |
| GO:0015991 | ATP hydrolysis coupled proton transport | 2.28E-04 | 3.91E-03 | 7.41 |
| GO:0008361 | regulation of cell size | 1.49E-05 | 3.38E-04 | 7.18 |
| GO:0009637 | response to blue light | 3.84E-06 | 9.48E-05 | 7.12 |
| GO:0019288 | isopentenyl diphosphate biosynthetic process, methylerythritol 4-phosphate pathway | 5.94E-27 | 6.26E-24 | 7.09 |

| | | | | |
|---|---|---|---|---|
| GO:0009240 | isopentenyl diphosphate biosynthetic process | 5.94E-27 | 4.69E-24 | 7.09 |
| GO:0046490 | isopentenyl diphosphate metabolic process | 5.94E-27 | 3.76E-24 | 7.09 |
| GO:0015995 | chlorophyll biosynthetic process | 1.80E-08 | 7.03E-07 | 6.96 |
| GO:0051656 | establishment of organelle localization | 8.76E-11 | 4.54E-09 | 6.89 |
| GO:0019682 | glyceraldehyde-3-phosphate metabolic process | 4.00E-38 | 1.26E-34 | 6.72 |
| GO:0009071 | serine family amino acid catabolic process | 4.28E-04 | 7.00E-03 | 6.67 |
| GO:0045036 | protein targeting to chloroplast | 6.02E-08 | 2.07E-06 | 6.4 |
| GO:0072596 | establishment of protein localization to chloroplast | 6.02E-08 | 2.05E-06 | 6.4 |
| GO:0072598 | protein localization to chloroplast | 6.02E-08 | 2.02E-06 | 6.4 |
| GO:0006098 | pentose-phosphate shunt | 1.48E-19 | 3.12E-17 | 6.38 |
| GO:0051156 | glucose 6-phosphate metabolic process | 1.48E-19 | 2.92E-17 | 6.38 |
| GO:0006739 | NADP metabolic process | 2.60E-19 | 4.84E-17 | 6.29 |
| GO:0009965 | leaf morphogenesis | 1.25E-08 | 5.05E-07 | 6.03 |
| GO:0042793 | transcription from plastid promoter | 2.13E-05 | 4.74E-04 | 5.93 |
| GO:0016556 | mRNA modification | 4.29E-16 | 4.84E-14 | 5.86 |
| GO:0010015 | root morphogenesis | 3.09E-04 | 5.25E-03 | 5.72 |
| GO:0006655 | phosphatidylglycerol biosynthetic process | 3.44E-05 | 7.25E-04 | 5.62 |
| GO:0046471 | phosphatidylglycerol metabolic process | 3.44E-05 | 7.20E-04 | 5.62 |
| GO:0009657 | plastid organization | 2.01E-18 | 3.18E-16 | 5.46 |
| GO:0009658 | chloroplast organization | 3.89E-14 | 3.32E-12 | 5.46 |
| GO:0046148 | pigment biosynthetic process | 7.13E-15 | 6.82E-13 | 5.37 |
| GO:0006081 | cellular aldehyde metabolic process | 9.22E-33 | 1.46E-29 | 5.35 |
| GO:0035303 | regulation of dephosphorylation | 6.10E-06 | 1.48E-04 | 5.34 |
| GO:0035304 | regulation of protein dephosphorylation | 6.10E-06 | 1.47E-04 | 5.34 |
| GO:0010103 | stomatal complex morphogenesis | 1.10E-09 | 5.04E-08 | 5.07 |
| GO:0090626 | plant epidermis morphogenesis | 1.10E-09 | 4.97E-08 | 5.07 |
| GO:0006779 | porphyrin-containing compound biosynthetic process | 6.66E-07 | 1.84E-05 | 4.96 |
| GO:0033014 | tetrapyrrole biosynthetic process | 6.66E-07 | 1.83E-05 | 4.96 |
| GO:0051640 | organelle localization | 4.81E-08 | 1.69E-06 | 4.85 |
| GO:0019344 | cysteine biosynthetic process | 2.00E-08 | 7.53E-07 | 4.83 |
| GO:0006090 | pyruvate metabolic process | 6.68E-21 | 2.11E-18 | 4.71 |
| GO:0016114 | terpenoid biosynthetic process | 1.49E-10 | 7.61E-09 | 4.65 |
| GO:0006534 | cysteine metabolic process | 4.11E-08 | 1.46E-06 | 4.63 |
| GO:0006633 | fatty acid biosynthetic process | 6.94E-05 | 1.34E-03 | 4.62 |
| GO:0006721 | terpenoid metabolic process | 2.98E-10 | 1.42E-08 | 4.51 |
| GO:0005984 | disaccharide metabolic process | 2.39E-10 | 1.20E-08 | 4.39 |
| GO:0009070 | serine family amino acid biosynthetic process | 1.12E-07 | 3.61E-06 | 4.36 |
| GO:0016226 | iron-sulfur cluster assembly | 9.25E-06 | 2.20E-04 | 4.33 |
| GO:0031163 | metallo-sulfur cluster assembly | 9.25E-06 | 2.18E-04 | 4.33 |
| GO:0000023 | maltose metabolic process | 1.10E-08 | 4.58E-07 | 4.3 |
| GO:0009069 | serine family amino acid metabolic process | 2.65E-08 | 9.74E-07 | 4.3 |
| GO:0009073 | aromatic amino acid family biosynthetic process | 5.48E-05 | 1.07E-03 | 4.3 |
| GO:0006733 | oxidoreduction coenzyme metabolic process | 4.55E-17 | 5.75E-15 | 4.28 |
| GO:0019362 | pyridine nucleotide metabolic process | 1.24E-14 | 1.15E-12 | 4.22 |
| GO:0046496 | nicotinamide nucleotide metabolic process | 1.24E-14 | 1.12E-12 | 4.22 |
| GO:0008299 | isoprenoid biosynthetic process | 6.12E-10 | 2.85E-08 | 4.21 |
| GO:0005982 | starch metabolic process | 5.99E-11 | 3.26E-09 | 4.19 |
| GO:0006818 | hydrogen transport | 7.43E-05 | 1.43E-03 | 4.17 |

| | | | | |
|---|---|---|---|---|
| GO:0015992 | proton transport | 7.43E-05 | 1.42E-03 | 4.17 |
| GO:0072524 | pyridine-containing compound metabolic process | 2.27E-14 | 1.99E-12 | 4.15 |
| GO:1902600 | hydrogen ion transmembrane transport | 1.81E-04 | 3.25E-03 | 4.14 |
| GO:0016144 | S-glycoside biosynthetic process | 1.81E-04 | 3.24E-03 | 4.14 |
| GO:0019758 | glycosinolate biosynthetic process | 1.81E-04 | 3.22E-03 | 4.14 |
| GO:0019761 | glucosinolate biosynthetic process | 1.81E-04 | 3.20E-03 | 4.14 |
| GO:0006720 | isoprenoid metabolic process | 1.11E-09 | 4.95E-08 | 4.11 |
| GO:0016143 | S-glycoside metabolic process | 9.96E-05 | 1.87E-03 | 4.04 |
| GO:0019760 | glucosinolate metabolic process | 9.96E-05 | 1.86E-03 | 4.04 |
| GO:0019757 | glycosinolate metabolic process | 9.96E-05 | 1.85E-03 | 4.04 |
| GO:0006644 | phospholipid metabolic process | 2.83E-20 | 6.40E-18 | 4 |
| GO:0008654 | phospholipid biosynthetic process | 7.39E-19 | 1.23E-16 | 3.99 |
| GO:0044550 | secondary metabolite biosynthetic process | 2.76E-07 | 8.38E-06 | 3.94 |
| GO:0019252 | starch biosynthetic process | 6.22E-09 | 2.66E-07 | 3.93 |
| GO:0006091 | generation of precursor metabolites and energy | 7.53E-12 | 4.67E-10 | 3.92 |
| GO:0042440 | pigment metabolic process | 7.76E-11 | 4.15E-09 | 3.91 |
| GO:0000272 | polysaccharide catabolic process | 4.86E-06 | 1.19E-04 | 3.78 |
| GO:0010075 | regulation of meristem growth | 2.65E-06 | 6.59E-05 | 3.75 |
| GO:0009311 | oligosaccharide metabolic process | 9.80E-09 | 4.13E-07 | 3.72 |
| GO:0006732 | coenzyme metabolic process | 2.50E-16 | 2.93E-14 | 3.69 |
| GO:0048509 | regulation of meristem development | 1.85E-06 | 4.68E-05 | 3.66 |
| GO:0009886 | post-embryonic morphogenesis | 1.27E-07 | 4.06E-06 | 3.64 |
| GO:0009699 | phenylpropanoid biosynthetic process | 5.32E-04 | 8.58E-03 | 3.64 |
| GO:0051188 | cofactor biosynthetic process | 3.78E-07 | 1.11E-05 | 3.56 |
| GO:0043085 | positive regulation of catalytic activity | 3.76E-05 | 7.76E-04 | 3.4 |
| GO:0070838 | divalent metal ion transport | 4.67E-05 | 9.53E-04 | 3.34 |
| GO:0072511 | divalent inorganic cation transport | 4.67E-05 | 9.46E-04 | 3.34 |
| GO:0044093 | positive regulation of molecular function | 4.67E-05 | 9.40E-04 | 3.34 |
| GO:0051186 | cofactor metabolic process | 1.73E-16 | 2.10E-14 | 3.32 |
| GO:0043623 | cellular protein complex assembly | 1.14E-09 | 4.98E-08 | 3.28 |
| GO:0009072 | aromatic amino acid family metabolic process | 1.68E-05 | 3.79E-04 | 3.28 |
| GO:0042744 | hydrogen peroxide catabolic process | 1.07E-04 | 1.96E-03 | 3.27 |
| GO:0044802 | single-organism membrane organization | 2.31E-11 | 1.30E-09 | 3.2 |
| GO:0015994 | chlorophyll metabolic process | 2.99E-04 | 5.11E-03 | 3.14 |
| GO:0009887 | organ morphogenesis | 5.70E-05 | 1.11E-03 | 3.13 |
| GO:0008610 | lipid biosynthetic process | 2.57E-20 | 6.24E-18 | 3.11 |
| GO:0006073 | cellular glucan metabolic process | 1.89E-08 | 7.30E-07 | 3.08 |
| GO:0044042 | glucan metabolic process | 1.89E-08 | 7.21E-07 | 3.08 |
| GO:0006364 | rRNA processing | 3.29E-07 | 9.80E-06 | 3.06 |
| GO:0016072 | rRNA metabolic process | 3.29E-07 | 9.71E-06 | 3.06 |
| GO:1901135 | carbohydrate derivative metabolic process | 1.64E-21 | 5.77E-19 | 3.05 |
| GO:0009250 | glucan biosynthetic process | 6.04E-07 | 1.70E-05 | 3.05 |
| GO:0007169 | transmembrane receptor protein tyrosine kinase signaling pathway | 1.00E-04 | 1.85E-03 | 2.99 |
| GO:0007167 | enzyme linked receptor protein signaling pathway | 1.00E-04 | 1.84E-03 | 2.99 |
| GO:0009653 | anatomical structure morphogenesis | 2.99E-12 | 2.05E-10 | 2.91 |
| GO:0006790 | sulfur compound metabolic process | 4.01E-08 | 1.44E-06 | 2.87 |
| GO:0019693 | ribose phosphate metabolic process | 2.46E-10 | 1.21E-08 | 2.84 |
| GO:0006461 | protein complex assembly | 9.12E-08 | 3.00E-06 | 2.77 |

| | | | | |
|---|---|---|---|---|
| GO:0044272 | sulfur compound biosynthetic process | 1.68E-06 | 4.39E-05 | 2.75 |
| GO:0000096 | sulfur amino acid metabolic process | 2.66E-05 | 5.87E-04 | 2.75 |
| GO:0000097 | sulfur amino acid biosynthetic process | 4.80E-05 | 9.60E-04 | 2.73 |
| GO:0019637 | organophosphate metabolic process | 3.28E-19 | 5.75E-17 | 2.69 |
| GO:0008652 | cellular amino acid biosynthetic process | 3.06E-07 | 9.22E-06 | 2.69 |
| GO:0019748 | secondary metabolic process | 2.05E-05 | 4.60E-04 | 2.64 |
| GO:0007166 | cell surface receptor signaling pathway | 4.36E-04 | 7.10E-03 | 2.63 |
| GO:0072593 | reactive oxygen species metabolic process | 2.10E-04 | 3.67E-03 | 2.61 |
| GO:0042743 | hydrogen peroxide metabolic process | 3.26E-04 | 5.47E-03 | 2.6 |
| GO:0044255 | cellular lipid metabolic process | 7.69E-16 | 8.38E-14 | 2.59 |
| GO:0005976 | polysaccharide metabolic process | 3.86E-10 | 1.82E-08 | 2.59 |
| GO:0048638 | regulation of developmental growth | 3.76E-04 | 6.25E-03 | 2.57 |
| GO:0006629 | lipid metabolic process | 8.66E-21 | 2.49E-18 | 2.56 |
| GO:0034622 | cellular macromolecular complex assembly | 4.12E-07 | 1.19E-05 | 2.56 |
| GO:0061024 | membrane organization | 1.26E-08 | 5.03E-07 | 2.55 |
| GO:0034660 | ncRNA metabolic process | 1.48E-07 | 4.59E-06 | 2.54 |
| GO:0031328 | positive regulation of cellular biosynthetic process | 3.21E-04 | 5.42E-03 | 2.52 |
| GO:0090407 | organophosphate biosynthetic process | 1.25E-11 | 7.47E-10 | 2.46 |
| GO:0071822 | protein complex subunit organization | 5.83E-07 | 1.66E-05 | 2.45 |
| GO:0034637 | cellular carbohydrate biosynthetic process | 1.21E-06 | 3.21E-05 | 2.45 |
| GO:0044264 | cellular polysaccharide metabolic process | 4.32E-07 | 1.24E-05 | 2.44 |
| GO:0040008 | regulation of growth | 3.54E-04 | 5.91E-03 | 2.43 |
| GO:0032787 | monocarboxylic acid metabolic process | 2.07E-12 | 1.46E-10 | 2.41 |
| GO:0000271 | polysaccharide biosynthetic process | 1.78E-06 | 4.56E-05 | 2.41 |
| GO:0044262 | cellular carbohydrate metabolic process | 1.08E-07 | 3.51E-06 | 2.4 |
| GO:0034470 | ncRNA processing | 3.12E-05 | 6.74E-04 | 2.36 |
| GO:0006753 | nucleoside phosphate metabolic process | 5.85E-08 | 2.03E-06 | 2.34 |
| GO:0009451 | RNA modification | 1.17E-06 | 3.13E-05 | 2.34 |
| GO:0016052 | carbohydrate catabolic process | 9.90E-05 | 1.87E-03 | 2.34 |
| GO:0006520 | cellular amino acid metabolic process | 8.96E-08 | 2.98E-06 | 2.33 |
| GO:0033692 | cellular polysaccharide biosynthetic process | 1.04E-05 | 2.40E-04 | 2.33 |
| GO:0055086 | nucleobase-containing small molecule metabolic process | 3.56E-08 | 1.29E-06 | 2.32 |
| GO:0009117 | nucleotide metabolic process | 1.43E-07 | 4.48E-06 | 2.29 |
| GO:1901607 | alpha-amino acid biosynthetic process | 1.92E-04 | 3.37E-03 | 2.29 |
| GO:0022607 | cellular component assembly | 1.33E-07 | 4.19E-06 | 2.27 |
| GO:0071555 | cell wall organization | 1.13E-05 | 2.58E-04 | 2.25 |
| GO:1901657 | glycosyl compound metabolic process | 5.00E-04 | 8.10E-03 | 2.24 |
| GO:0043436 | oxoacid metabolic process | 9.27E-16 | 9.77E-14 | 2.23 |
| GO:0006082 | organic acid metabolic process | 9.27E-16 | 9.45E-14 | 2.23 |
| GO:0019752 | carboxylic acid metabolic process | 3.52E-15 | 3.48E-13 | 2.23 |
| GO:0065003 | macromolecular complex assembly | 1.03E-05 | 2.40E-04 | 2.22 |
| GO:1901605 | alpha-amino acid metabolic process | 3.52E-05 | 7.32E-04 | 2.22 |
| GO:0009893 | positive regulation of metabolic process | 2.93E-05 | 6.44E-04 | 2.18 |
| GO:0016053 | organic acid biosynthetic process | 7.46E-07 | 2.03E-05 | 2.17 |
| GO:0046394 | carboxylic acid biosynthetic process | 7.46E-07 | 2.01E-05 | 2.17 |
| GO:0016051 | carbohydrate biosynthetic process | 1.69E-06 | 4.37E-05 | 2.17 |
| GO:0071554 | cell wall organization or biogenesis | 9.63E-06 | 2.25E-04 | 2.14 |
| GO:0044711 | single-organism biosynthetic process | 1.49E-21 | 5.88E-19 | 2.12 |
| GO:0045229 | external encapsulating structure organization | 4.88E-05 | 9.69E-04 | 2.09 |

| | | | | |
|---|---|---|---|---|
| GO:0044281 | small molecule metabolic process | 3.18E-17 | 4.19E-15 | 2.04 |
| GO:0016071 | mRNA metabolic process | 1.14E-04 | 2.05E-03 | 2 |
| GO:0006796 | phosphate-containing compound metabolic process | 9.94E-14 | 7.30E-12 | 1.97 |
| GO:0044283 | small molecule biosynthetic process | 6.31E-07 | 1.76E-05 | 1.97 |
| GO:0006793 | phosphorus metabolic process | 8.06E-14 | 6.06E-12 | 1.96 |
| GO:1901564 | organonitrogen compound metabolic process | 3.28E-11 | 1.82E-09 | 1.86 |
| GO:0005975 | carbohydrate metabolic process | 2.06E-07 | 6.31E-06 | 1.73 |
| GO:1901566 | organonitrogen compound biosynthetic process | 2.30E-06 | 5.78E-05 | 1.73 |
| GO:0055114 | oxidation-reduction process | 9.41E-05 | 1.79E-03 | 1.66 |
| GO:0044710 | single-organism metabolic process | 2.88E-17 | 3.96E-15 | 1.6 |
| GO:0071840 | cellular component organization or biogenesis | 1.31E-09 | 5.68E-08 | 1.6 |
| GO:0016043 | cellular component organization | 1.44E-08 | 5.70E-07 | 1.59 |
| GO:1901576 | organic substance biosynthetic process | 1.37E-11 | 8.03E-10 | 1.57 |
| GO:0009058 | biosynthetic process | 1.47E-11 | 8.44E-10 | 1.54 |
| GO:0044249 | cellular biosynthetic process | 2.47E-10 | 1.20E-08 | 1.54 |
| GO:0044767 | single-organism developmental process | 3.87E-05 | 7.94E-04 | 1.53 |
| GO:0032502 | developmental process | 3.22E-05 | 6.84E-04 | 1.51 |
| GO:0044763 | single-organism cellular process | 8.29E-11 | 4.36E-09 | 1.41 |
| GO:1901360 | organic cyclic compound metabolic process | 8.09E-06 | 1.94E-04 | 1.37 |
| GO:0044699 | single-organism process | 1.73E-12 | 1.24E-10 | 1.35 |
| GO:0006725 | cellular aromatic compound metabolic process | 2.93E-05 | 6.39E-04 | 1.35 |
| GO:0006807 | nitrogen compound metabolic process | 5.18E-05 | 1.02E-03 | 1.31 |
| GO:0008152 | metabolic process | 1.05E-11 | 6.40E-10 | 1.28 |
| GO:0044238 | primary metabolic process | 2.36E-08 | 8.77E-07 | 1.28 |
| GO:0071704 | organic substance metabolic process | 1.11E-08 | 4.55E-07 | 1.27 |
| GO:0044237 | cellular metabolic process | 1.32E-06 | 3.48E-05 | 1.24 |
| GO:0009987 | cellular process | 1.82E-06 | 4.63E-05 | 1.19 |
