# Supplementary material for: C3 cotyledons are followed by C4 leaves: intra-individual transcriptome analysis of Salsola soda (Chenopodiaceae)
Source: J Exp Bot. 2016 Sep 22;68(2):161–76. doi: 10.1093/jxb/erw343 (PMC5853821; doi:10.1093/jxb/erw343)
Supplement: Supplementary_Table_S10 [file erw343_suppl_supplementary_table_s10.pdf]

TF family	Annotation (TAIR 9)	Locus	yS mean	Cot mean	1L mean	2L mean	oL mean	Cluster
bHLH	basic helix-loop-helix (bHLH) family protein	AT2G40200	0.07	6.81	2.37	1.28	1.45	1
bZIP	HYH (HY5-HOMOLOG)	AT3G17609	1.25	100.71	9.57	7.54	9.12	1
MADS	SVP (SHORT VEGETATIVE PHASE)	AT2G22540	3.64	21.04	7.77	5.62	5.19	1
MYB	MYB86 (myb domain protein 86)	AT5G26660	0.31	8.96	0.76	0.68	0.46	1
Orphans	zinc finger (B-box type) family protein	AT4G39070	3.35	41.77	7.04	5.77	6.76	1
TRAF	BTB/POZ domain-containing protein	AT1G21780	3.32	31.21	12.46	8.99	9.76	1
AP2-EREBP	EBP (ETHYLENE-RESPONSIVE ELEMENT BINDING PROTEIN)	AT3G16770	149.35	165.44	31.61	18.00	34.34	18
AP2-EREBP	RAP2.6L (related to AP2 6L)	AT5G13330	6.51	11.93	1.79	0.49	0.77	18
AP2-EREBP	RAP2.12	AT1G53910	44.14	150.75	47.37	38.73	72.79	18
AP2-EREBP	RAV1	AT1G13260	6.78	19.07	4.77	3.60	7.14	18
ARF	ARF6 (AUXIN RESPONSE FACTOR 6)	AT1G30330	21.84	42.00	16.97	16.90	19.03	18
ARF	ARF8	AT5G37020	16.37	48.62	11.79	12.34	13.70	18
AUX/IAA	IAA5 (INDOLE-3-ACETIC ACID INDUCIBLE 5)	AT1G15580	6.33	24.08	4.97	3.01	3.37	18
bHLH	basic helix-loop-helix (bHLH) family protein	AT3G21330	1.93	35.58	0.89	0.03	0.32	18
bHLH	FRU (FER-LIKE REGULATOR OF IRON UPTAKE)	AT2G28160	0.48	11.96	0.31	0.23	0.19	18
bZIP	BZIP2 (ARABIDOPSIS THALIANA BASIC LEUCINE-ZIPPER 2)	AT2G18160	2.47	3.79	0.93	0.57	1.08	18
bZIP	HY5 (ELONGATED HYPOCOTYL 5)	AT5G11260	17.98	138.06	17.13	8.62	27.56	18
GRF	GRF1 (GROWTH-REGULATING FACTOR 1)	AT2G22840	6.21	16.48	3.41	2.90	4.46	18
HB	HB20 (ARABIDOPSIS THALIANA HOMEOBOX PROTEIN 20)	AT3G01220	19.28	75.71	6.42	3.30	5.64	18
HB	HB23	AT1G26960	27.87	110.74	9.27	4.17	5.32	18
HB	HDG2 (HOMEODOMAIN GLABROUS 2)	AT1G05230	10.49	12.87	4.94	3.89	6.27	18
HB	PDF2 (PROTODERMAL FACTOR 2)	AT4G04890	15.29	19.48	7.55	5.24	9.37	18
MYB	MYB26	AT3G13890	0.76	1.59	0.11	0.02	0.05	18
MYB	MYB55	AT4G01680	0.39	9.76	0.60	0.46	0.32	18
MYB	MYB61	AT1G09540	0.42	7.40	0.68	0.21	0.16	18
MYB	MYB103	AT1G63910	0.08	1.30	0.10	0.03	0.06	18
Orphans	AHK5 (ARABIDOPSIS HISTIDINE KINASE 5)	AT5G10720	3.29	6.00	1.72	1.29	1.21	18
Orphans	similar to unknown protein [A. thaliana] (TAIR:AT5G53420.1); contains InterPro domain CCT	AT4G27900	14.61	86.12	3.62	0.80	4.42	18
Orphans	STH2 (SALT TOLERANCE HOMOLOG2)	AT1G75540	37.38	84.06	13.88	14.10	24.12	18

SWI/SNF-BAF60b	zinc finger (C3HC4 type RING finger) family protein	AT5G63700	1.55	15.82	1.76	1.38	1.81	18
TRAF	BOP2 (BLADE ON PETIOLE2)	AT2G41370	1.63	19.41	0.30	0.39	1.29	18
WRKY	WRKY57	AT1G69310	0.30	4.60	0.48	0.43	0.81	18
C2C2-GATA	CYTOKININ-RESPONSIVE GATA FACTOR 1	AT4G26150	7.49	6.53	12.96	10.22	10.57	2
C2H2	GLABROUS INFLORESCENCE STEMS 2	AT5G06650	1.63	0.29	1.69	2.50	0.96	2
C2H2	IDD7 (Arabidopsis thaliana Indeterminate(ID)-Domain 7)	AT1G55110	25.46	14.80	29.34	24.34	36.83	2
C2H2	IDD12	AT4G02670	31.84	16.08	29.50	25.38	35.50	2
C2H2	ZFP7 (ZINC FINGER PROTEIN 7)	AT1G24625	1.92	1.19	5.66	3.72	2.59	2
C2H2	ZFP8	AT2G41940	0.26	0.25	1.19	1.52	0.85	2
GNAT	GCN5-related N-acetyltransferase (GNAT), putative	AT5G13780	65.74	35.41	63.64	58.33	53.00	2
GRAS	SHORT ROOT	AT4G37650	2.36	2.16	11.78	8.14	3.20	2
LOB	LBD38 (LOB DOMAIN-CONTAINING PROTEIN 38)	AT3G49940	7.61	4.71	19.04	10.47	7.84	2
LOB	LBD39	AT4G37540	29.03	15.44	62.98	34.29	27.44	2
MADS	AGL104 (AGAMOUS-LIKE 104)	AT1G22130	0.09	0.18	0.96	0.88	0.46	2
Orphans	RESPONSE REGULATOR 3	AT2G41310	8.37	4.71	9.82	14.58	5.31	2
Trihelix	transcription factor	AT3G54390	5.19	3.58	6.56	7.74	4.96	2
AP2-EREBP	AP2 domain-containing transcription factor, putative	AT1G71450	0.03	0.36	0.95	1.63	1.02	3
bHLH	DNA binding / transcription factor	AT4G05170	0.20	1.36	15.70	12.71	9.93	3
bHLH	phytochrome interacting factor 4	AT2G43010	0.03	0.75	1.79	1.77	2.20	3
C2H2	zinc finger (C2H2 type) family protein	AT3G45260	12.58	10.10	21.91	18.44	27.11	3
HB	BELL 1	AT5G41410	0.49	2.42	5.94	5.40	9.60	3
HB	BELL 1-LIKE HOMEODOMAIN 4	AT2G23760	0.47	4.92	17.85	13.13	25.89	3
HB	BELL 1-LIKE HOMEODOMAIN 7	AT2G16400	1.30	11.67	23.89	25.88	46.90	3
HB	KNAT5	AT4G32040	2.27	2.48	5.81	4.86	8.85	3
HB	KNOTTED1-LIKE HOMEOBOX GENE 4	AT5G11060	17.42	20.25	48.89	41.43	74.44	3
MADS	AGL6	AT2G45650	0.06	0.18	1.73	2.78	5.37	3
MADS	AGL14	AT4G11880	0.20	1.55	19.81	25.80	44.65	3
MADS	AGL24	AT4G24540	1.55	10.98	23.20	22.60	33.02	3
MADS	AGL42	AT5G62165	0.22	0.43	1.92	2.60	4.47	3
MADS	AGL72	AT5G51860	0.00	0.03	0.69	1.02	1.54	3
MYB-related	CIRCADIAN CLOCK ASSOCIATED 1	AT2G46830	3.57	9.59	53.11	44.29	40.22	3
NAC	ANAC067 (Arabidopsis NAC domain containing protein 67)	AT4G01520	0.51	0.90	2.82	2.03	5.40	3

NAC	ANAC083	AT5G13180	4.42	11.97	31.98	26.63	52.69	3
Orphans	similar to unnamed protein product [Vitis vinifera] (GB:CAO23408.1); contains InterPro domain WRC	AT2G42040	0.41	0.80	4.08	3.10	2.20	3
TCP	TCP10 (TEOSINTE BRANCHED1, CYCLOIDEA, AND PCF FAMILY 10)	AT2G31070	0.55	2.46	12.73	10.29	8.95	3
WRKY	WRKY DNA-binding protein 41	AT4G11070	0.03	0.09	1.55	0.57	2.80	3
bHLH	basic helix-loop-helix (bHLH) family protein	AT4G01460	1.66	2.67	7.04	5.37	3.77	7
C2C2-CO-like	zinc finger (B-box type) family protein	AT1G73870	0.64	9.74	23.73	17.60	22.05	7
C2C2-YABBY	AFO (ABNORMAL FLORAL ORGANS)	AT2G45190	1.12	10.05	19.43	16.48	11.55	7
C2C2-YABBY	YAB2 (YABBY2)	AT1G08465	0.37	5.64	10.55	9.55	8.44	7
C2C2-YABBY	YAB3 (YABBY3)	AT4G00180	1.01	7.32	19.50	16.06	10.73	7
C2C2-YABBY	YAB5 (YABBY5)	AT2G26580	1.70	43.36	90.39	109.80	87.02	7
C2H2	zinc finger (C2H2 type) family protein	AT1G75710	1.19	12.26	35.07	26.72	20.59	7
C3H	zinc finger (CCCH-type) family protein	AT3G51950	1.95	13.16	25.53	22.23	19.95	7
CCAAT	Heme activator protein (yeast) homolog 5A	AT3G48590	13.66	34.21	55.20	57.94	36.37	7
GNAT	GNAT family protein	AT1G72030	0.06	7.41	11.21	11.38	10.36	7
MYB	MYB60	AT1G08810	1.63	2.00	5.58	3.99	2.39	7
MYB	MYB111	AT5G49330	0.00	0.30	0.91	0.92	0.00	7
Orphans	similar to CIL [Arabidopsis thaliana] (TAIR:AT4G25990.1); contains InterPro domain CCT	AT5G14370	17.72	28.18	41.67	38.23	28.97	7
SET	SET domain-containing protein	AT5G14260	5.78	30.46	48.77	41.91	32.25	7
Sigma70-like	RNA POLYMERASE SIGMA SUBUNIT 2	AT1G08540	20.50	46.60	67.94	60.79	42.46	7
SWI/SNF-BAF60b	SWIB complex BAF60b domain-containing protein	AT2G14880	15.02	54.59	82.67	61.22	46.69	7
TCP	PLASTID TRANSCRIPTION FACTOR 1	AT3G02150	0.03	1.19	4.30	3.30	2.32	7
TCP	TCP family transcription factor, putative	AT2G45680	0.32	0.70	2.68	1.08	0.73	7
TCP	TCP family transcription factor, putative	AT5G51910	0.89	1.83	8.40	3.50	2.18	7
TCP	TCP3	AT1G53230	0.29	3.18	17.54	12.86	7.07	7
TCP	TCP17	AT5G08070	0.09	1.26	5.89	6.21	3.29	7
TCP	TCP24	AT1G30210	0.47	2.48	6.29	6.28	4.71	7
